# Using BpyAla to generate copper artificial metalloenzymes: a catalytic and structural study†

**DOI:** 10.1039/d3cy01648j

**Published:** 2024-01-29

**Authors:** E. Klemencic, R. C. Brewster, H. S. Ali, J. M. Richardson, A. G. Jarvis

**Affiliations:** a EaStCHEM School of Chemistry, University of Edinburgh Joseph Black Building David Brewster Road The King's Buildings Edinburgh EH9 3FJ UK amanda.jarvis@ed.ac.uk; b School of Biological Sciences, University of Edinburgh Swann Building Edinburgh EH9 3BF UK

## Abstract

Artificial metalloenzymes (ArMs) have emerged as a promising avenue in the field of biocatalysis, offering new reactivity. However, their design remains challenging due to the limited understanding of their protein dynamics and how the introduced cofactors alter the protein scaffold structure. Here we present the structures and catalytic activity of novel copper ArMs capable of (*R*)- or (*S*)-stereoselective control, utilizing a steroid carrier protein (SCP) scaffold. To incorporate 2,2′-bipyridine (Bpy) into SCP, two distinct strategies were employed: either Bpy was introduced as an unnatural amino acid (2,2′-bipyridin-5-yl)alanine (BpyAla) using amber stop codon expression or *via* bioconjugation of bromomethyl-Bpy to cysteine residues. The resulting ArMs proved to be effective at catalysing an enantioselective Friedel–Crafts reaction with SCP_Q111BpyAla achieving the best selectivity with an enantioselectivity of 72% *ee* (*S*). Interestingly, despite using the same protein scaffold, different attachment strategies for Bpy at the same residue (Q111) led to a switch in the enantiopreference of the ArM. X-ray crystal structures of SCP_Q111CBpy and SCP_Q111BpyAla ArMs with bound Cu(ii) ions unveiled crucial differences in the orientation of the catalytic centre. Combining structural information, alanine scanning studies, and computational analysis shed light on the distinct active sites of the ArMs, clarifying that these active sites stabilise the nucleophilic substrate on different sides of the electrophile leading to the observed switch in enantioselectivity. This work underscores the importance of integrating structural studies with catalytic screening to unravel the intricacies of ArM behaviour and facilitate their development for targeted applications in biocatalysis.

## Abstract 2

Changing the synthetic strategy to access artificial metalloenzymes containing Cu–bipyridine active sites, changes the enantioselectivity in a Friedel–Crafts alkylation.

## Graphical abstract

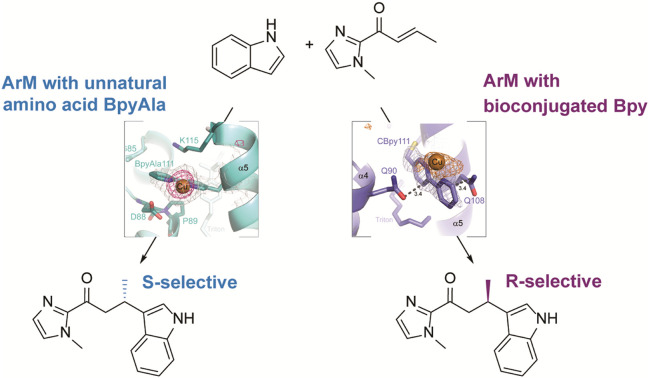

## Introduction

Artificial metalloenzymes (ArMs) represent an avenue to new-to-nature reactions.^1–4^ These systems expand the biocatalytic toolbox by using transition metal complexes tethered to protein scaffolds; notable examples of chemical transformations using ArMs include transfer hydrogenation,^5^ hydroformylation,^6,7^*in vivo* metathesis,^8^ lignin oxidation,^9^ Friedel–Crafts alkylation^10^ and other cross-coupling reactions.^11^

Site-specific coordination of synthetic metal complexes to proteins plays a crucial role in the development of stereoselective artificial metalloenzymes (ArMs), wherein the protein scaffold forms a secondary coordination sphere around the reactive centre providing stereocontrol.^12,13^ To synthesise ArMs, researchers have used many different approaches from simply leveraging native metal-binding activity found in certain proteins^14^ to combining synthetic chemistry and proteins, including using variants of natural cofactors, such as the iron-binding protoporphyrin IX, with alternative transition metals.^15,16^ More synthetic approaches to site-specific incorporation of metal complexes include the utilisation of supramolecular binding, with several different tethered transition metal complex systems explored to date, the most notable being the biotin–streptavidin (Sav) system.^17^ Bioconjugation of metal complexes *via* covalent attachment with unique reactive amino acid residues such as cysteine or azidophenylalanine is another widely used approach to prepare ArMs.^18^*In vivo* ligand incorporation offers an attractive alternative where metal-binding unnatural amino acids such as (2,2′-bipyridin-5-yl)alanine (BpyAla) can be selectively introduced to the protein scaffold directly using genetic code expansion technologies, allowing for more streamlined enzyme engineering.^19,20^

Within these different approaches to ArM design, several scaffolds have been repeatedly used with various attachment strategies for transformation into novel catalysts. These scaffolds have been described as ‘privileged’ scaffolds, in a manner analogous with privileged ligands in homogeneous catalysis. The most prominent examples are the LmrR scaffold, which allows supramolecular, bioconjugation, and incorporation of unnatural amino acids,^21^ and Sav, which has been explored in dative coordination,^22^ alongside the more common supramolecular approach.^17^ Despite these advances, few comparative studies have been conducted which included direct comparisons of the modification strategy for site-selective metal coordination. This makes it difficult to discern the role of protein modification upon ArM reactivity and predict the most effective route to ArM assembly for a desired application.

The Kamer and Jarvis groups have extensively studied the human steroid carrier protein (SCP-2L),^23^ a single-domain 13.5 kDa protein containing a hydrophobic tunnel, as a scaffold for ArM design.^24^ It has been exploited in the design of ArMs for selective hydroformylation using Rh–phosphine organometallic complexes,^6^ which showed high activity and selectivity in the production of long chain linear aldehydes, under aqueous conditions. Moreover, engineering the scaffold for improved thermostability incurred a five-fold increase in TON compared to the wild type.^7^ This protein has also been used in ArMs for the selective oxidation of lignin model compounds,^9^ for enantioselective Cu-catalysed Diels–Alder reactions,^25^ and artificial photoenzyme design.^26^ Other carrier proteins such as adipocyte lipid binding protein (ALBP),^27^ ferric hydroxamate uptake protein component A (FhuA),^28^ and maltose binding protein^29^ have been used in the design of ArMs suggesting that proteins with a carrier function may also serve as privileged scaffolds. In this work, the potential of SCP-2L to serve as a privileged protein scaffold is explored, through studying the copper-catalysed 1,4-addition of indole to enones.

**Fig. 1:**
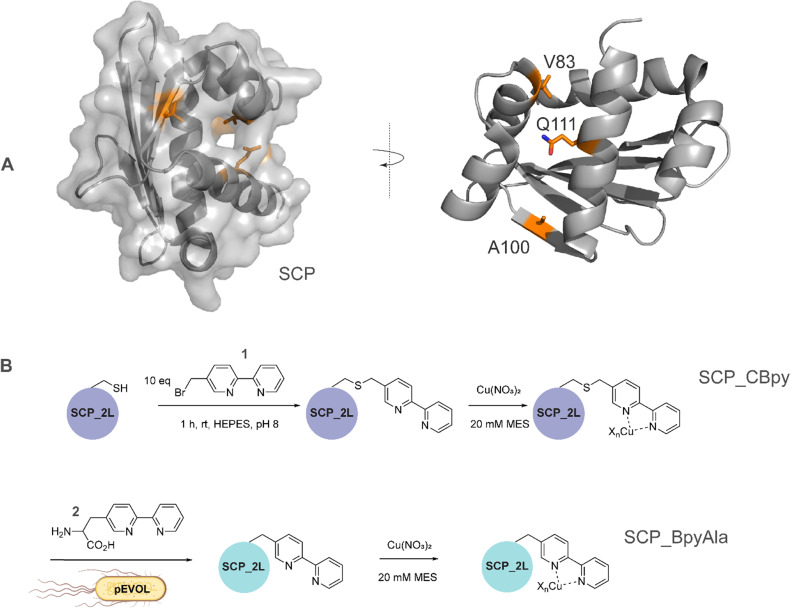
A) SCP-2L and the residues (orange) used for site-selective ligand attachment (PDB 1IKT). B) Comparison of modification approaches for SCP_Cu ArM design: (upper panel) *in vitro* bioconjugation of unique Cys residues with 1 before addition of Cu^2+^ ions in a two-step protocol; (lower panel) one-step *in vivo* site-selective incorporation of the unnatural amino acid BpyAla 2.

Bipyridine (Bpy) is a well-studied ligand for transition metals and is utilised in the coordination sphere of many catalytic complexes.^30^ It can be introduced into a protein *via* the bioconjugation of bromomethylbipyridines at cysteine residues (Fig. 1). Bpy is also one of very few metal ligands that has been incorporated into the genetic code by amber stop codon suppression. Here two methods for site-selective incorporation of bipyridine into the SCP-2L scaffold were compared, and the copper-catalysed 1,4-addition of indole to enones was used to analyse ArM activity. Differences in stereoselectivities were identified with results rationalised using ArM crystal structures and DFT simulations.

## Results and discussion

### Preparation of SCP-Cu artificial metalloenzymes

**Fig. 2:**
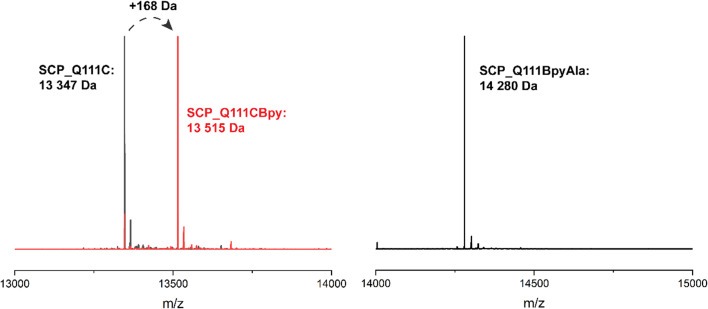
Mass spectra of SCP ArMs. Left panel: mass spectrum of SCP_Q111C (black line: expected mass: 13 347 Da) and bioconjugation products from incorporation of 1 into SCP_Q111C (red line; expected mass after bioconjugation: 13 515 Da, 10% remained unmodified). Right panel: incorporation of BpyAla during translation produced a single species – SCP_Q111BpyAla (expected mass after purification: 14 280 Da. Note: the mass of the BpyAla proteins is not 46 Da lower than the SCP CBpy proteins as might be expected due to the absence of the –CH_2_S– linker, but higher due to the presence of additional amino acids from the TEV site remaining at the C-terminus.).

The steroid carrier protein type 2 like (SCP-2L) domain of the human multifunctional enzyme 2 (MFE-2) has previously been used as a protein scaffold for both regioselective and enantioselective reactions using covalent modification through the introduction of unique cysteine residues.^31^ A number of residues in the scaffold's apolar tunnel (V83, A100, Q111) have previously been identified as being amenable to mutation and modification.^6^ Their location along the apolar tunnel provides an ideal starting point for the introduction of bipyridine into the protein scaffold to create an active site within a protein pocket. Three different steroid carrier proteins, each containing a unique cysteine at position Q111C, A100C or V83C, were prepared as described previously.^6^ Briefly, pEHISTEV plasmids containing the SCP gene inserted after an N-terminal His_6_ tag with TEV cleavage site were transformed into Rosetta DE3 *E. coli* cells. His_6_-tagged proteins were expressed in production broth media and purified by Ni-affinity chromatography with the His_6_ tag subsequently removed using TEV protease; a further purification step by Ni-affinity chromatography gave the final proteins in good yields (20–70 mg L^−1^ of media). Bipyridine was introduced into the protein scaffold through bioconjugation of the cysteine residues with 10 equivalents of 5-bromomethyl-2,2′-bipyridine 1 in HEPES buffer at pH 8 (Fig. 1B). The optimal conversion was approximately 70–85% modified protein, with minimal secondary modification (<5% secondary modification; the remaining mass balance is unreacted protein, see Fig. 2 and ESIa† Fig. S4).

In comparison to bioconjugation, genetic code expansion using stop codon suppression provides a method of directly incorporating Bpy as the amino acid BpyAla 2 during protein expression (Fig. 1B).^32^ The synthesis of BpyAla was optimised to allow BpyAla production on scales of 10 grams without the need for column chromatography (see ESIa† for full details).

The SCP-2L gene was codon-optimised for *E. coli* and prepared by gene synthesis. Amber stop codons were introduced at A100, V83 or Q111 by site-directed mutagenesis. *E. coli* BL21 (DE3) cells were co-transformed with the pEVOL-BpyAla plasmid, which contains the orthogonal *Mj*TyrRS/*Mj*tRNA^Tyr^ genes,^32^ and the pET28 plasmid carrying the SCP gene with a C-terminal TEV cleavable His_6_ tag. The genes were expressed in the presence of 0.5 mM BpyAla^20^ and the resulting proteins purified by Ni-affinity chromatography with the His_6_ tag removed using TEV-protease. Typical yields were 5–15 mg L^−1^ in LB media, representing a decrease of 20–80% compared to the yields of SCP-2L without BpyAla. Proteins containing unnatural amino acids are known to have lower expression yields, due to incomplete stop codon supression giving truncated protein.^19^ Mass spectrometry on the purified proteins confirmed the successful incorporation of BpyAla at residue positions 100, 111, or 83 (Fig. 2 and ESIa† Fig. S2).

To obtain the Cu-metalloproteins, one equivalent of Cu(NO_3_)_2_ was added directly to the Bpy-containing SCP proteins. Copper binding was confirmed by UV-vis spectroscopy which clearly showed a red shift in the π–π* transition of the Bpy ligand in the presence of Cu(ii), consistent with previous reports on copper binding to Bpy-containing proteins.^20^ Titration experiments confirmed that, at the concentrations of interest (20–100 μM), Cu(ii) bound to the Bpy-containing SCP proteins with ∼1 : 1 stoichiometry (see ESIa,† Fig. S5 to S10). Copper binding was also confirmed by ICP-MS analysis (see ESIa†).

### Structural analysis

**Fig. 3:**
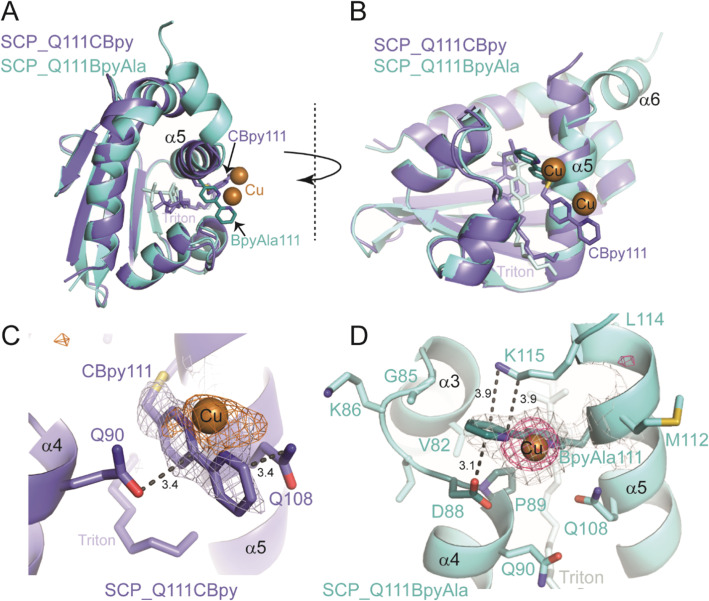
Crystal structures of Cu(ii)-bound SCP_Q111CBpy and SCP_Q111BpyAla. A) Top view of superimposed structures of SCP_Q111CBpy (purple) and SCP_Q111BpyAla (cyan) looking down the tunnel, with Triton bound. The atoms of CBpy, BpyAla and Triton are shown as sticks. Orange spheres represent the Cu(ii) ions. B) Side-view of the superimposed structures. C) Close-up view of CBpy111 in SCP_Q111CBpy structure. The anomalous difference map (contoured at 3*σ*) is shown as an orange mesh. D) Close-up view of BpyAla111 in the SCP_Q111BpyAla structure. A Cu omit map (contoured at 3.6*σ*) is shown as a pink mesh. In C and D the grey mesh represents the 2*F*_o_–*F*_c_ electron density map (contoured at 1*σ*).

Whilst, the UV-vis spectroscopy and ICP-MS studies showed copper binding to the ArMs (see ESIa†), they give no information about the precise environment of the copper. We therefore looked to X-ray crystallography to obtain structural understanding of the newly created active sites within the metalloproteins. Crystals of SCP_Q111CBpy and SCP_Q111BpyAla were soaked in solutions of Cu(ii) ions and their structures determined by X-ray crystallography (Fig. 3A and B). Triton-X-100 was needed for crystallisation, similar to wt SCP-2L (PDB ID: 1IKT),^23^ the apolar tunnel is occupied by Triton X-100 in the structures of Cu(ii)-bound SCP_Q111CBpy and SCP_Q111BpyAla (Fig. 3).

The structure of Cu(ii)-bound SCP_Q111CBpy was determined to 1.52 Å resolution, and clearly confirmed the incorporation of 2,2′-bipyridine at C111, which is positioned at the centre of the C-terminal helix, α5. The bipyridine rings are sandwiched in a parallel fashion between the amide side-chain atoms of Q108 (one turn up on α-helix 5) and Q90 on α-helix 4 (Fig. 3C). The outer and inner pyridine rings are 3.4 Å from Q108 and Q90 respectively, a distance that favours formation of stabilising aromatic π–amino electrostatic interactions. α-Helix 5 is slightly shifted relative to its position in the wt SCP_2L structure (PDB ID 1IKT) (Fig. S14 in ESIa†) and is more flexible than the remainder of the structure, with B-factors ranging from 52–96 Å^2^ from the N- to the C-terminus of the helix (N103-L120), compared to 75 Å^2^ for the protein overall. The B-factor of the Bpy moiety is 92 Å^2^, suggesting flexibility in its position. Additional electron density was observed in the 2*F*_o_–*F*_c_ map around the solvent-exposed face of the Bpy moiety, and a single peak in an anomalous difference map confirmed this is a bound Cu(ii) ion (Fig. 3C). The Cu(ii) atom lies in the same plane as the two pyridine rings and is 2 Å from the two pyridine ring nitrogens, and thus we assume that the Cu(ii) ion adopts an octahedral coordination geometry. The high B-factor (108 Å) of the Cu(ii) is consistent with its position in the flexible α-helix 5. Only one water molecule could be modelled around the Cu(ii) ion, but the positions of Q108 and Q90 suggest that their amide side-chain atoms may play a role in coordinating the other (unmodelled) waters in the coordination sphere.

The structure of Cu(ii)-bound SCP_Q111BpyAla was determined to 2.51 Å resolution (Fig. 3B and D) and revealed a different orientation of the bipyridine moiety compared to that seen in the Cu(ii)-bound SCP_Q111CBpy structure. Despite the lower resolution, the electron density for the BpyAla bipyridine rings, α-helix 5 and the additional C-terminal amino acids (120–128) was very clear, indicative of a more rigid conformation for these parts of the protein. The co-planar BpyAla bipyridine rings are wedged into a shallow pocket formed by the turn between G85 and P89 at the edge of the apolar tunnel. This conformation is stabilised by van der Waals interactions between C1 of BpyAla and both G85 Hα (3.35 Å) and D88 Hα (3.36 Å), C2 of BpyAla and Hδ of P89 (2.87 Å: 3.36 Å to the Cδ) and C3 of BpyAla and Hγ of P89 (2.74 Å: 3.26 Å to the Cγ) (Fig. 3D). In addition, the side-chains of D88 and K115 (one turn down on α-helix 5) lie on either face of the bipyridine and may stabilise the BpyAla conformation by aromatic–π electrostatic interactions.

The 2*F*_o_–*F*_c_ electron density map showed a single intense peak adjacent to the BpyAla bipyridine corresponding to a Cu(ii) ion coordinated to N1 and N2 of BpyAla (Cu–N bond distance of 2 Å) and four water molecules in an octahedral arrangement (Fig. S15 in ESIa†). The B-factors for the Bpy moiety and the Cu(ii) ion (43 Å^2^ and 57.91 Å^2^, respectively) were lower in this structure compared to the bipyridine rings in the Cu(ii)-bound Q111CBipy structure, indicating that the catalytic environment is more rigid.

Taken together the structural analyses confirm the incorporation of Bpy at position 111 and the binding of Cu(ii) to the Bpy moieties in both SCP ArMs. They also revealed marked differences in the environments of the Bpy and bound Cu(ii) ions in the SCP_Q111BpyAla and SCP_Q111CBpy structures. Moreover, the Bpy moiety at position 111 is more rigid when incorporated as the unnatural amino acid BpyAla than *via* bioconjugation at a cysteine.

### Catalytic screening

The catalytic activities of Bpy-containing SCP metalloenzymes were evaluated in the Cu-catalysed Friedel–Crafts alkylation of 5-methoxy-1*H*-indole 3 with 1-(1-methyl-1*H*-imidazol-2-yl)but-2-en-1-one 4, resulting in the product 5, the benchmark reaction for Cu(ii) catalysis (Table 1).^33^ Reactions in the absence of Cu showed minimal activity even at room temperature, verifying the role of copper at the catalytic centre. Enantioselective product formation was only observed in the presence of the Bpy-containing SCP-metalloenzymes.

**Table 1:** Copper catalysed enantioselective Friedel–Crafts alkylation using bipyridine containing SCP proteins

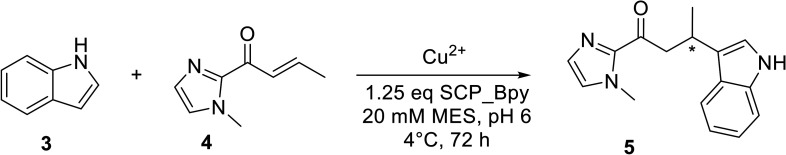			
Entry	Ligand/protein	Yield of 5^a^ (%)	e.r.^b^ (*R* : *S*)
1	None	76 (±6)	50 : 50 (±0)
2	BpyAla(rac)	50 (±2)	50 : 50 (±0)
3	wt SCP_2L	43 (±5)	50 : 50 (±0)
4^c^	SCP_A100CBpy	42 (±1)	66 : 34 (±4)
5^c^	SCP_V83CBpy	28 (±1)	52 : 48 (±2)
6^d^	SCP_Q111CBpy	25 (±2)	64 : 36 (±4)
7	SCP_A100BpyAla	45 (±3)	51 : 49 (±0)
8	SCP_V83BpyAla	45 (±8)	63 : 37 (±1)
9	SCP_Q111BpyAla	42 (±7)	20 : 80 (±1)
10	SCP_Q111CBpy + Triton	30 (±8)	57 : 43 (±0)
11	SCP_Q111BpyAla + Triton	32 (±3)	23 : 77 (±3)
12	SCP_A100BpyAla + Triton	21 (±3)	52 : 48 (±1)
13	SCP_V83BpyAla + Triton	16 (±5)	57 : 43 (±3)
14^e^	SCP_A100CBpy without Cu	6.1	n.d.
15^e^	SCP_A100BpyAla without Cu	1.9	n.d.

a Yield obtained by HPLC using 2-phenylquinoline as internal standard.

b e.r. determined using chiral HPLC. n.d. not determined.

c Cu(NO_3_)_2_ 72 μM.

d Cu(NO_3_)_2_ 63 μM.

e rt reaction. *S* and *R* were assigned using chiral HPLC.^34^

SCP ArMs with BpyAla showed a higher yield (42–45%) compared to the equivalent Cys-coupled Bpy SCP ArMs. Among the novel ArMs, SCP_Q111BpyAla displayed the greatest enantioselectivity with an e.r. of 20 : 80 (±1), favouring the *S* enantiomer, and a yield of 42% (Table 1, entry 9). The BpyAla moiety in this SCP ArM is situated at the centre of one side of the tunnel, in the middle of α-helix 5 (Fig. 1A and 3A). By contrast, the ArMs with BpyAla situated at either end of the tunnel showed either no enantioselectivity in the case of SCP_A100BpyAla (Table 1, entry 7), or lower enantioselectivity with a preference for the *R* enantiomer (Table 1, entry 8). We hypothesise that the lower selectivity at the entries to the tunnel could be due to increased flexibility and space leading to less defined binding pocket, compared to position Q111 in the centre of the tunnel. While enantioselectivity towards the *R* enantiomer was observed with all three Cys mutants, it was limited with the best result observed for SCP_A100CBpy which gave a 66 : 34 ratio (Table 1, entries 4–6).

Both enantiomers of 5 can be obtained in the Friedel–Crafts alkylation using SCP-ArMs with Bpy attached to residue 111, but by using different attachment strategies (Table 1, entries 6 and 9). The crystal structures of SCP_Q111BpyAla and SCP_Q111CBpy provide a structural indication for the observed differences in the enantioselectivities of these ArMs [almost 2× higher e.e., from 64 : 36 (*R*) for SCP_Q111CBpy to 20 : 80 (*S*) for SCP_Q111BpyAla]. The longer linker to the protein backbone in SCP_Q111CBpy, which has an additional –CH_2_–S– compared to SCP_Q111BpyAla, allows it to extend out from α-helix 5 and adopt a more exposed position on the surface of the protein, compared to the BpyAla which is closer to the scaffold protein. In addition, the longer linker confers greater flexibility (as reflected in the B-factors), explaining the lower enantiomeric excess. Alignment of the Cu(ii)-bound SCP_Q111BpyAla and SCP_Q111CBpy structures (Fig. 3A and B) highlights the different positions and opposite orientations of the Bpy moieties, despite being incorporated at the same amino acid.

### Computational modelling of metalloenzyme-catalysed Friedel–Crafts alkylation

In order to gain more information on how the ArMs control enantioselectivity, computational modelling was used to probe the stereochemical outcomes for the Cu-catalysed Friedel–Crafts reaction. The crystal structure coordinates were used for the computations. As these included Triton-X100, the ArM-catalysed Friedel–Crafts reactions were repeated in the presence of 2 eq. Triton-X100 to ascertain if this was likely to change the catalysis and thus if the crystal structures are a reasonable structural starting point. In the case of SCP_Q111BpyAla and SCP_Q111CBpy, the same enantioselectivities were observed as reactions without Triton-X100, with only a slight decrease in yield suggesting that the structures are a valid model to use to rationalise the differences in enantioselectivity. However, for SCP_A100BpyAla and SCP_V83BpyAla there was a significant decrease in activity, which could be caused by Triton-X100 blocking the availability of Cu-Bpy to the substrates as it binds within the protein tunnel (Table 1, entries 10–13).^7^

The first step of the reaction is the conjugate addition of indole 3 to enone 4 to form intermediate (**Int1**), *via* a transition state (**TS1**) (Scheme S1 ESIb†). The second step is the product formation by the protonation reaction *via* a second transition state (**TS2**). To investigate the enantioselectivity of the Friedel–Craft alkylation, we looked to prior work using cluster models for metalloenzyme-catalyzed reactions.^35,36^ The same QM-cluster model technique along with density functional theory (DFT) methodology was utilised to study our reaction. Three active site models were created for analysis (Fig. S1 ESIb†). Model A represents a reaction catalysed by Cu(ii)-2,2′-bipyridine, in the absence of protein, whereas models B and C represent reactions catalysed by Cu(ii)-bound SCP_Q111BpyAla and SCP_Q111CBpy, respectively. The computational analysis on Model A (Fig. S11 ESIb†) showed that the conjugate coupling step is both the rate-determining step as well as the enantioselective step, and the formation of the keto product is most likely. This agrees well with the experimental results (Table 2) as well as previous studies,^37^ therefore only the first step was modelled going forwards. To create models B and C, the indole 3 and enone 4 substrates, along with Cu(ii) ions, were incorporated into the crystal structures of Cu(ii)-bound SCP_Q111BpyAla and SCP_Q111CBpy, respectively. The PDB files of the apo SCP_ArMs were prepared as described in the ESIb,† and 3 and 4 were docked using AutoDock Vina,^37^ with the lowest energy docked poses used for molecular dynamics (MD) simulations (see ESIb† for detailed methods and results).

**Table 2:** Enantioselectivity in Friedel-crafts reactions catalysed by SCP_Q111BpyAla mutant ArMs

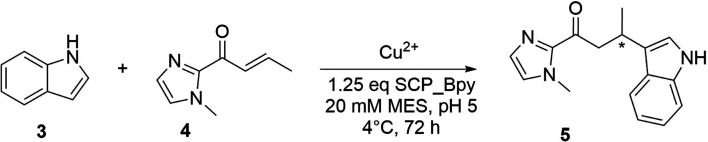			
Entry	Mutant of SCP_Q111BpyAla	Yield of 5^a^ (%)	e.r.^b^ (*R* : *S*)
1	None	43 (±2)	14 : 86 (±1)
2	Q108A	38 (±7)	28 : 72 (±0)
3	V82A	33 (±2)	24 : 76 (±1)
4	F34A	21 (±1)	31 : 69 (±0)
5	M112A	33 (±4)	19 : 81 (±0)
6	K115A	41 (±5)	27 : 73 (±1)
7	D88A	54 (±10)	18 : 82 (±1)

a Yield obtained by HPLC using 2-phenylquinoline as internal standard; standard deviations are shown in brackets.

b e.r. determined using chiral HPLC.

**Fig. 4:**
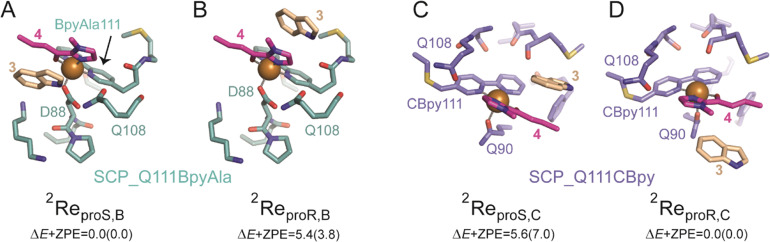
Optimized geometries of SCP_Q11BpyAla catalysed reactions (Model B): A) ^2^Re_pro*S*,B_ and B) ^2^Re_pro*R*,B_, and SCO_Q111CBpy catalysed reactions (Model C): C) ^2^Re_pro*S*,C_ and D) ^2^Re_pro*R*,C_. The pro*S* and pro*R* subscripts indicate the substrate-binding configuration and the superscript 2 represents the doublet spin state. Relative energies (in kcal mol^−1^) are UB3LYP/BS2//UB3LYP/BS1 values with zero-point energy (ZPE) included. Orange spheres represent the Cu(ii) ions.

For SCP_Q111BpyAla catalysed reactions (Model B), the reactant complexes in two conformations: pro*S* and pro*R* were selected and their geometries optimized to give **Re**_**pro*S*,B**_ and **Re**_**pro*R*,B**_ as shown in Fig. 4A and B. The **Re**_**pro*S*,B**_ structure is lower in energy by 5.4 kcal mol^−1^ (Δ*E* + ZPE) than **Re**_**pro*R*,B**_ and therefore **Re**_**pro*S*,B**_ represents the more favourable substrate binding orientation.

**Fig. 5:**
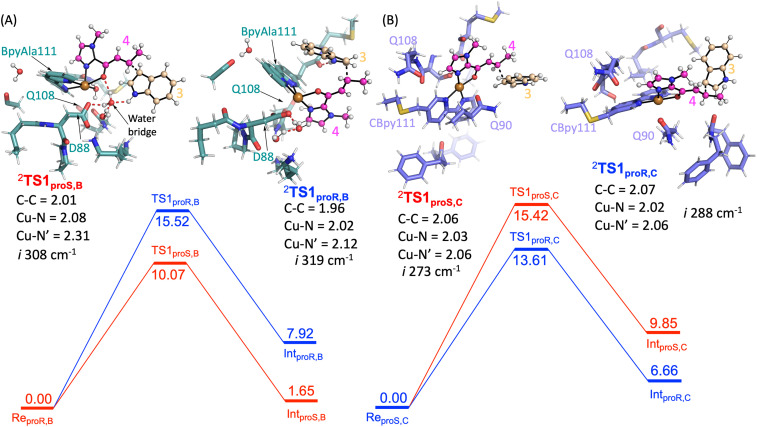
Potential energy landscape for the Friedel–Crafts alkylation by A) SCP_Q111BpyAla and B) SCP_Q111CBpy. Energy values are in kcal mol^−1^. The optimized geometric structures of the transitions are shown on the left and right of free energy landscape with their important bond lengths in Å. The imaginary frequency represents the stretching vibration of C–C bonds between 3 and 4 for each transition state.

Next, the Friedel–Craft (FC) alkylation reaction catalysed by SCP_Q111BpyAla, using **Re**_**pro*S*,B**_ and **Re**_**pro*R*,B**_ reactants as the starting structures, was calculated. Firstly, the conjugate addition of indole 3 to enone 4, where substrate 3 configuration is either pro*S* or pro*R* was tested. The C–C coupling transition states (**TS1**_**pro*S*,B**_ and **TS1**_**pro*R*,B**_) of 3 with 4 leads to an intermediate for each configuration: **Int1**_**pro*S*,B**_ and **Int1**_**pro*R*,B**_ respectively. The calculated potential energy landscape for the conjugate coupling reaction for Model B is represented, along with the optimized geometric structures of these transition states, in Fig. 5A. The energy barrier for **TS1**_**pro*S*,B**_ (10.1 kcal mol^−1^) is lower than **TS1**_**pro*R*,B**_ (15.5 kcal mol^−1^) (values relative to the stable configuration **Re**_**Pro*S*,B**_). The transition states were characterized by an imaginary frequency *i*308 cm^−1^ and *i*319 cm^−1^ for **TS1**_**pro*S*,B**_ and **TS1**_**pro*R*,B**_ respectively. Furthermore, in transition state **TS1**_**pro*S*,B**_, indole 3 has strong water bridge interactions with D88 that make it more stable than the **TS1**_**pro*R*,B**_, which has a week hydrogen bonding interaction with M112. Subsequently, the transition states are relaxed to their respective intermediates (**Int1**_**pro*S*,B**_ and **Int1**_**pro*R*,B**_), which are characterized by lower relative energy values: 1.65 and 7.92 kcal mol^−1^, respectively. Thus, the pro*S* configuration pathway is energetically favoured over the pro*R* configuration pathway. Taken together these analyses explain the preferred enantioselectivity of SCP_Q111BpyAla (e.r. 14 : 86, *R* : *S*) that we observed experimentally (Table 2).

In a similar manner, the FC alkylation reaction mechanism catalysed by SCP_Q111CBpy (Model C) was explored. The reactant complexes in both pro*S* and pro*R* configurations were selected as the starting structures. The optimized geometries of these reactant complexes **Re**_**pro*S*,C**_ and **Re**_**pro*R*,C**_ are represented in Fig. 4C and D. The free energy of the **Re**_**pro*S*,C**_ structure is higher than the **Re**_**pro*R*,C**_ by 5.6 kcal mol^−1^ (Δ*E* + ZPE), indicating that the reactant complex of model **C** in pro*R* configuration shows the strongest substrate-bound pose and hence signifies the favoured reactant orientation.

Finally, the FC reaction pathway of Cu(ii)-bound SCP_Q111CBpy was explored, using **Re**_**pro*S*,C**_ and **Re**_**pro*R*,C**_ reactants as the starting structures. The calculated potential energy landscape for the conjugate coupling reaction for model **C** is represented, along with the optimized geometric structures of these transition states, in Fig. 5B. The lowest energy barrier (13.6 kcal mol^−1^) was obtained for the pro*R* C–C coupling transition state (**TS1**_**pro*R*,C**_) while the energy barrier for the pro*S* transition state (**TS1**_**pro*S*,C**_) was higher (15.4 kcal mol^−1^). The transition states are characterized by the presence of an imaginary frequencies *i*288 cm^−1^ and *i*273 cm^−1^ for **TS1**_**pro*R*,C**_ and **TS1**_**pro*S*,C**_ respectively. Furthermore, in the **TS1**_**pro*R*,C**_ transition state a hydrophobic interaction from M105 was observed with 3, while no such interaction of 3 was observed in **TS1**_**pro*S*,C**_. The system then relaxed to the low energy state intermediates **Int1**_**pro*R*,C**_ and **Int1**_**pro*S*,C**_, which are characterized in the local minima state by the presence of all real frequency values indicating stable structures. The **Int1**_**pro*R*,C**_ has lower energy (6.7 kcal mol^−1^) than the **Int1**_**pro*S*,C**_ configuration (9.8 kcal mol^−1^), which favours the pro*R* configuration pathway over the pro*S* pathway. This is consistent with the experimentally observed preference to produce the *R* enantiomer by SCP_Q111CBpy (Table 1).

Overall, the substrates' activation in FC alkylation mechanism with SCP_Q111BpyAla favours the formation of pro*S* product over pro*R* while with SCP_Q111CBpy favours pro*R* product formation over pro*S*. However, the products distribution was achieved *via* a competitive pathway, so a mixture of both enantiomers of the product is predicted, which agrees well with our experimental results. The larger difference in the relative energies for the SCP_Q111BpyAla TS intermediates matches the experimental observation of improved enantioselectivity when using SCP_Q111BpyAla as the catalyst over the use of SCP_Q111CBpy.

### Engineering SCP_Q111BpyAla

Taking the most promising ArM (SCP_Q111BpyAla) forwards, we chose to use a structure-based alanine scanning approach to see if any of the residues identified from either the computational or structural work impacted activity or selectivity.^39^ Each amino acid was substituted individually with alanine *via* site-directed mutagenesis, and the yields and enantioselectivities in Friedel–Crafts reactions catalysed by these mutant SCP ArMs were determined (Table 2). These reactions were carried out at pH 5, as slightly improved enantiomeric ratios were observed compared to those performed at pH 6 (Table 2 entry 1 *vs.*Table 1 entry 9) matching previous observations by the Roelfes group.^40^

None of the mutations screened showed substantial variations (*i.e.* a complete drop in reactivity or selectivity). Indeed, the mutation with the biggest difference was F34A which was chosen due to its location nearby and within the protein hydrophobic pocket (Table 2, entry 4). SCP_Q111BpyAla F34A exhibited substantial precipitation suggesting that disruption to the protein core reduced stability, leading to the observed low activity and selectivity. A similar rationale was also used for the choice of V82A which gave lower activity and selectivity but not to the same extent (Table 2, entry 3 and 4). The crystal structure suggested that K115 and D88 may play a role in stabilising the bipyridine position within the protein through aromatic–π electrostatic interactions. Mutating D88 to alanine gave no meaningful change in enantioselectivity (Table 2, entry 7), whilst K115A showed a reduction in enantioselectivity (Table 2, entry 6). The more flexible nature of the lysine side chain means it is less clear if this can be attributed to structural changes as opposed to some role with substrate binding. The computational work revealed no interaction of the substrates with K115. In contrast, a close contact between the side chain of D88 and the copper atom was observed in the modelled transition states and D88 was shown to participate in water bonding networks with the substrates. Both these interactions would be disrupted on mutation of D88 to alanine, suggesting that either these interactions are of minor importance in catalysis or alternative residues such as Q108 could replace the hydrogen bonding interactions with water. Whilst Q108A was shown to reduce the enantioselectivity obtained with SCP-Q111BpyAla experimentally (Table 2, entry 2), no clear role was observed in the computational work.

**Fig. 6:**
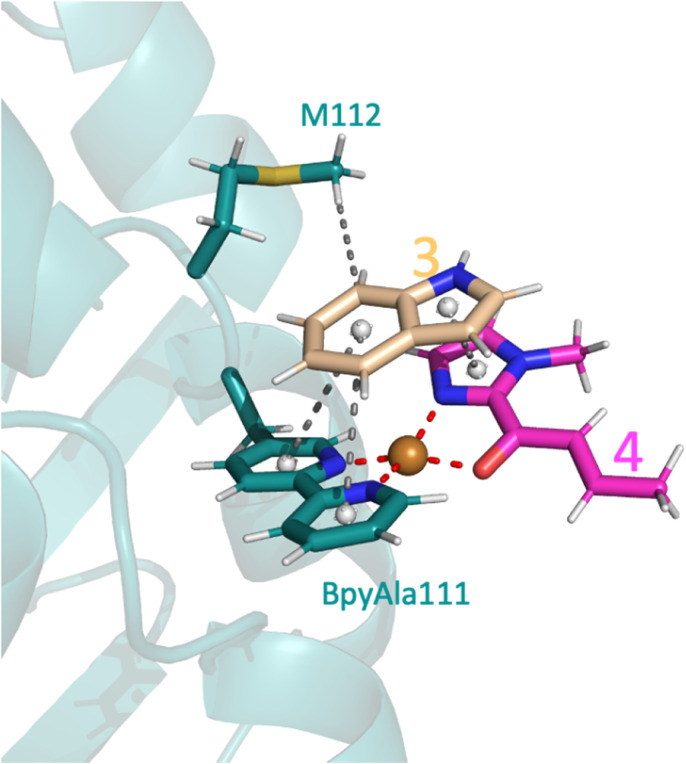
Docking pose of 3 and 4 with the SCP_Q111BpyAla ArM, highlighting the key interactions of 3 including one hydrophobic interaction with M112 and three π–π interactions, two with BpyAla111 and one with 4 (represent by grey spheres).

The only amino acid shown to make direct contact with the substrate during the course of the reaction was M112: the methyl makes a weak hydrophobic interaction with 3 during substrate binding in the pro*S* pathway (Fig. 6), whilst the amide backbone of M112 makes a weak hydrogen bond with 3 in the pro*R* transition state (Fig. 5A). Mutating M112 to alanine gave a small reduction in enantioselectivity (Table 2, entry 5). Whilst alanine can also make hydrophobic interactions, its reduced chain length would preclude interactions with 3 in this instance and thus M112 could be helping to stabilise the substrates to a small extent. No difference would be expected for the pro*R* pathway as alanine can still participate in amide backbone bonding.

The lack of side chain interactions with the substrates and no clear results from the alanine scanning suggests that a rational approach to designing the active site *via* mutagenesis may not lead to an improved enzyme. Indeed, the exposed nature of the active site on the side of the protein suggests that extensive backbone engineering to build up the protein bulk around the substrates may lead to more promising candidates for enantioselective catalysis.

## Conclusions

Using two different approaches to forming artificial metalloenzymes, a library of successful catalysts for the Cu-catalysed Friedel–Crafts alkylation were obtained. Moderate activities and good enantiomeric ratios were observed with SCP_Q111BpyAla giving the highest e.r. of 14 : 86 in favour of the *S* enantiomer. In general, lower enantioselectivities were observed for the cysteine-modified catalysts and this is attributed to increased flexibility around the active site due to the additional S–CH_2_ linker. Changing from genetic incorporation of BpyAla at Q111 to bioconjugation of bipyridine through a cysteine, resulted in an unexpected change in the major isomer observed from *S* to *R*, albeit with a reduction in enantiomeric excess. This result is intriguing as a major challenge in enzyme catalysis is accessing the opposite product enantiomer. Crystallographic and computational studies confirm that changes in the structure of the active site led to the stabilisation of indole 3 on different sides of the bound enone 4, with the enantiomeric ratios observed correlating with the difference in the calculated transition state energies for the pro*R* and pro*S* SCP_Q111BpyAla and SCP_Q111CBpy Cu catalyst models. Based on these findings, future work should prioritize determining the most suitable modification method for the desired application of the ArM before proceeding with reaction screening and optimization, as subtle differences in protein structure can substantially change the stabilised transition state.

The crystal structure obtained of SCP-Q111BpyAla_Cu is a rare example of a protein structure containing an unnatural amino acid. Our work shows that increasing our understanding of artificial metalloenzyme structures facilitates their development as catalysts, and moving forwards will be vital to enable future *de novo* design of metalloproteins.

## Author contributions

AGJ conceived, supervised, and obtained funding for the project. All authors contributed to data analysis and preparing the manuscript. JMR obtained and analysed the X-ray crystal diffraction data, and uploaded the data to the PDB. EK and RCB designed the experiments and collected the data for the protein production, modification, and catalysis. HSA carried out the computational aspects of the project.

## Conflicts of interest

There are no conflicts to declare.

## Supplementary Material

CY-014-D3CY01648J-s001

CY-014-D3CY01648J-s002
