# Supplementary material for: Using BpyAla to generate copper artificial metalloenzymes: a catalytic and structural study
Source: Catal Sci Technol. 2024 Jan 29;14(6):1622–32. doi: 10.1039/d3cy01648j (PMC10946309; doi:10.1039/d3cy01648j)
Supplement: CY-014-D3CY01648J-s001 [file CY-014-D3CY01648J-s001.pdf]

Electronic Supplementary Material (ESI) for Catalysis Science & Technology.
This journal is © The Royal Society of Chemistry 2024

Supporting Information A

Using BpyAla to generate Copper Artificial Metalloenzymes: a catalytic and structural study

E. Klemencic[1], R. C. Brewster[1], H. S. Ali[1,2], J. M. Richardson[3], A. G. Jarvis[1]*
[1]School of Chemistry, Joseph Black Building, Kings Buildings, University of Edinburgh, David Brewster Road, Edinburgh, EH9 3FJ. [2] Current address: Chemistry Research Laboratory, Department of Chemistry and the INEOS Oxford Institute for Antimicrobial Research, University of Oxford, 12 Mansfield Road, Oxford OX1 3TA, UK. [3] School of Biological Sciences, Swann Building, University of Edinburgh, Edinburgh, EH9 3BF.
*Corresponding author: amanda.jarvis@ed.ac.uk

1 General Remarks 3
2 Molecular Biology 3
2.1 SCP_2L Cysteine mutants 4
2.2 Gene optimization and SCP_2L TAG mutants 5
2.3 Site-directed mutagenesis 5
3 Protein expression and purification 6
3.1 SDS PAGE of protein purification 8
3.2 ESI-mass spectra of SCP mutants 9
4 Scanning of the Bioconjugation Conditions 10
5 Bioconjugation of Cys Mutants 10
6 Characterization of Cu(II) Binding to Conjugated Cys Mutants and TAG Mutants 12
6.2 ICP analysis 15
7 Friedel-Crafts Alkylation 16
7.1 pH effect on enantioselecitivity 18
8 Alanine scan 18
8. 1 Protein Sequences of SCP_Q111BpyAla Ala mutants 19
9 Size exclusion chromatography of SCP_Q111BpyAla 20
10 Crystallization 20
10. 1 X-ray crystal structure determination and refinement 21
11 Synthesis of BpyAla 24
1-(2-pyrdylacetyl)pyridinium iodide, S1 24
5-methyl-2,2’-bipyridine, S2 25
5-bromomethyl-2,2-bipyridine, S3 25
Diethyl 2-(2,2´-bipyridin-5-ylmethyl)-2-acetamidomalonate, S4 26
(2,2´-bipyridin-5-yl)alanine·3HCl, 2 27

1 General Remarks

Commercially available chemicals used in the study were purchased from commercial suppliers (Sigma-Aldrich, Fluorochem).

NMR spectra were recorded on a Bruker Avance III 500 or 400 MHz spectrometer at 300 K. Chemical shifts are reported in parts per million (ppm) and referenced to the residual solvent peaks: $CDCl_3$ (^{1}H: δ 7.26 ppm), D_2O (1H: δ 4.79 ppm).Coupling constants (J) are reported in Hertz (Hz) and were calculated using MestReNova (version 14.2.0) and rounded to the nearest 0.1 Hz. The following abbreviations (and their combinations) are used to label the multiplicities: s (singlet), d (doublet), t (triplet), q (quartet) and m (multiplet) as observed in the spectra. The molecular weight of the protein mutants was determined using electrospray ionization mass spectroscopy (ESI-MS) analysis on a Waters Synapt G2 with Waters Acquity I-Class UPLC (LC-MS). ICP-MS data was collected on a Agilent 7500 ce. Enantiomeric ratio of the reaction were analysed using HPLC (Chiralpak AD-H, 250 x 4.6 mm) at a flow rate of 1 ml/min using n-hexane:iPrOH 85:15 as a solvent and the products were detected at wavelength of 280 nm. Chemically competent *E. coli* DH5α cells were used for cloning, *E. coli* BL21 and Rosetta 2 DE3 cells for protein expression. Primers for site-directed mutagenesis were purchased from Sigma Aldrich, USA and IDT, USA. PCR Master Mix and DpnI were purchased from NEB, USA. GeneJet Plasmid Minikit for plasmid extraction was purchased from ThermoFisher Scientific, USA. HisTrap HP Ni-charged immobilized metal affinity column (IMAC) was purchased from GE Healthcare, USA. The mass of proteins was calculated using the Expasy ProtParam tool (http://web.expasy.org/protparam/).

2 Molecular Biology

All the described research has been performed using SCP_2L as a protein scaffold. The metal binding moiety was introduced to the protein scaffold by either bioconjugation of cysteine mutants with the Cys codon at A100C, V83C, Q111C; or by genetic code expansion using amber stop codon suppression: TAG mutants with A100TAG, V83TAG, and Q111TAG. The sequences for TAG mutants were codon-optimized for expression in *E. coli*.

2.1 SCP_2L Cysteine mutants[1]

The Cys mutants have an N-terminal His-tag. The portion removed by TEV protease is highlighted in red, and the TEV protease recognition site highlighted in italics.

DNA sequence of SCP_A100C

ATGTCGTACTACCATCACCATCACCATCACGATTACGATATCCCAACGACCGAAAACCTGTATTTTCA
GGGCGCCATGGAGGGAGGGAAGCTTCAGAGTACCTTTGTATTTGAGGAAATAGGACGCCGCCTAAAGG
ATATTGGGCCTGAGGTGGTGAAGAAAGTAAATGCTGTATTTGAGTGGCATATAACCAAAGGCGGAAAT
ATTGGGGCTAAGTGGACTATTGACCTGAAAAGTGGTTCTGGAAAAGTGTACCAAGGCCCTGCAAAAGG
TGCTGCTGATACAACAATCATACTTTCAGATGAAGATTTCATGGAGGTGGTCCTGGGCAAGCTTGACC
CTCAGAAGGCATTCTTTAGTGGCAGGCTGAAG**TGC**AGAGGGAACATCATGCTGAGCCAGAAACTTCAG
ATGATTCTTAAAGACTATGCCAAGCTCTGA

Protein sequence of SCP_A100C

MSYYHHHHHHDYDIPTT***ENLYFQG***AMEGGKLQSTFVFEEIGRRLKDIGPEVVKKVNAVFEWHITKGGN
IGAKWTIDLKSGSGKVYQGPAKGAADTTIILSDEDFMEVVLGKLDPQKAFFSGRLK**C**RGNIMLSQKLQ
MILKDYAKL-

DNA sequence of SCP_V83C

ATGTCGTACTACCATCACCATCACCATCACGATTACGATATCCCAACGACCGAAAACCTGTATTTTCA
GGGCGCCATGGAGGGAGGGAAGCTTCAGAGTACCTTTGTATTTGAGGAAATAGGACGCCGCCTAAAGG
ATATTGGGCCTGAGGTGGTGAAGAAAGTAAATGCTGTATTTGAGTGGCATATAACCAAAGGCGGAAAT
ATTGGGGCTAAGTGGACTATTGACCTGAAAAGTGGTTCTGGAAAAGTGTACCAAGGCCCTGCAAAAGG
TGCTGCTGATACAACAATCATACTTTCAGATGAAGATTTCATGGAGGTGTGCCTGGGCAAGCTTGACC
CTCAGAAGGCATTCTTTAGTGGCAGGCTGAAGGCCAGAGGGAACATCATGCTGAGCCAGAAACTTCAG
ATGATTCTTAAAGACTATGCCAAGCTCTGA

Protein sequence of SCP_V83C

MSYYHHHHHHDYDIPTT***ENLYFQG***AMEGGKLQSTFVFEEIGRRLKDIGPEVVKKVNAVFEWHITKGGN
IGAKWTIDLKSGSGKVYQGPAKGAADTTIILSDEDFMEVCLGKLDPQKAFFSGRLKARGNIMLSQKLQ
MILKDYAKL-

DNA sequence of SCP_Q111C

ATGTCGTACTACCATCACCATCACCATCACGATTACGATATCCCAACGACCGAAAACCTGTATTTTCA
GGGCGCCATGGAGGGAGGGAAGCTTCAGAGTACCTTTGTATTTGAGGAAATAGGACGCCGCCTAAAGG
ATATTGGGCCTGAGGTGGTGAAGAAAGTAAATGCTGTATTTGAGTGGCATATAACCAAAGGCGGAAAT
ATTGGGGCTAAGTGGACTATTGACCTGAAAAGTGGTTCTGGAAAAGTGTACCAAGGCCCTGCAAAAGG
TGCTGCTGATACAACAATCATACTTTCAGATGAAGATTTCATGGAGGTGGTCCTGGGCAAGCTTGACC
CTCAGAAGGCATTCTTTAGTGGCAGGCTGAAGGCCAGAGGGAACATCATGCTGAGCCAGAAACTTTGC
ATGATTCTTAAAGACTATGCCAAGCTCTGA

Protein sequence of SCP_Q111C

MSYYHHHHHHDYDIPTT***ENLYFQG***AMEGGKLQSTFVFEEIGRRLKDIGPEVVKKVNAVFEWHITKGGN
IGAKWTIDLKSGSGKVYQGPAKGAADTTIILSDEDFMEVVLGKLDPQKAFFSGRLKARGNIMLSQKLC
MILKDYAKL-

2.2 Gene optimization and SCP_2L TAG mutants

The SCP_A100C gene was synthesized at Genescript and included a C-terminal TEV site and His_6-tag. The codons were optimized to E. coli codon bias. The gene was cloned into the vector pET28. Due to the change in site of the His_6 tag and TEV protease recognition site from the N to the C termini of the protein the sequence varies slightly between the cysteine and TAG genes

Optimized DNA sequence of C-ter His SCP_A100C

ATGGAGGGTGGCAAGCTGCAAAGCACCTTCGTGTTCGAGGAGATCGGTCGTCGCCTGAAAGACATCGG
CCCGGAAGTGGTTAAGAAAGTTAACGCGGTGTTCGAGTGGCACATCACCAAGGGTGGCAACATTGGTG
CGAAATGGACCATCGACCTGAAGAGCGGTAGCGGCAAAGTTTATCAAGGTCCGGCGAAGGGTGCGGCG
GATACCACCATCATTCTGAGCGACGAGGATTTTATGGAAGTGGTTCTGGGCAAGCTGGACCCGCAAAA
AGCGTTCTTTAGCGGTCGTCTGAAATGCCGTGGCAACATTATGCTGAGCCAAAAACTGCAAATGATTC
TGAAGGATTATGCGAAGCTGGGATCCGAAAACCTGTATTTTCAGGGCCTCGAGCACCACCACCACCAC
CACTGA

Protein sequence C-ter His SCP_A100C

MEGGKLQSTFVFEEIGRRLKDIGPEVVKKVNAVFEWHITKGGNIGAKWTIDLKSGSGKVYQGPAKGAA
DTTIILSDEDFMEVVLGKLDPQKAFFSGRLKARGNIMLSQKLQMILKDYAKLGS***ENLYFQG***LEHHHHH
H-

2.3 Site-directed mutagenesis

The 'wild-type' SCP containing no cysteine and a C-terminal His_6 tag, C-ter His SCP_C100A, was prepared by site directed mutagenesis. The mutant A100TAG was prepared using site-directed mutagenesis directly from the *E.coli* optimised C-ter His SCP_A100C gene, whilst the V83TAG, and Q111TAG mutants of SCP_2L were prepared from C-ter His SCP_C100A. The primers used for mutagenesis are summarized in **Table S1**.

Table S1: Primers used to make A100TAG, V83TAG, Q111TAG and C100A mutants.

Entry	Primer	Sequence
**1**	SCP_**A100TAG_Fw**	TCTGAAATAGCGTGGCAACATTATGCTGAGCCAAAAACTG
**2**	SCP_**A100TAG_Rv**	TTGCCACGCTATTTCAGACGACCGCTAAAGAACGCTTTT
**3**	SCP_**V83TAG_Fw**	GGAAGTGTAGCTGGGCAAGCTGGACCCGCAAAAAGC
**4**	SCP_**V83TAG_Rv**	GCCCAGCTACACTTCCATAAAATCCTCGTCGCTCAGAA
**5**	SCP_**Q111TAG_Fw**	CAAAAACTGTAGATGATTCTGAAGGATTATGCGAAGCTGG
**6**	SCP_**Q111TAG_Rv**	GAATCATCTACAGTTTTTGGCTCAGCATAATGTTGCCACG
**7**	SCP_**C100A_Fw**	TCTGAAA*GCA*CGTGGCAACATTATGCTGAGCCAAAAACTG
**8**	SCP_**C100A_Rv**	TTGCCACG*TGC*TTTCAGACGACCGCTAAAGAACGCTTTT

TAG denotes the position for incorporation of unnatural amino acid.

The PCR protocol for site-saturated mutagenesis was designed according to Hautings [2] method with two Ram cycles. The PCR reaction mixture was prepared: PCR Master Mix (1x), primers (100 nM each), template plasmid DNA (total mass of 40 ng), and H_2O to the final volume of 30 µL. The PCR program that was followed has two Ram cycles: initial denaturation at 94 °C for 7 min. Ram1: (12 cycles) starts with initial denaturation at 94 °C for 1 min, followed by annealing at 55 °C for 1 min, and elongation at 72 °C for 10 min. Ram2 (3 cycles) starts with initial denaturation at 95 °C for 1 min, annealing of the primers at 51°C for 1 min, and annealing at 72°C for 10 min. The PCR program ends with 10 min at 72 °C and a hold at 4 °C. PCR product was incubated overnight with 10 units of DpnI. *E. coli* DH5α were transformed and plated, a single colony of freshly transformed cells was cultured overnight in 5 ml LB media containing 50 µg/ml of kanamycin at 37 °C, 200 rpm. The plasmid DNA was isolated using plasmid extraction kit and sent for sequencing with 20 µL of the plasmid (concentration 30 ng/µL) to Dundee DNA Sequencing and Services, using facility provided T7 primer.

3 Protein expression and purification

Cysteine mutants of the SCP-2L scaffold were expressed following the protocol in [1], repeated here for clarity. For the expression of A100C, V83C, Q111C, chemically competent *E. coli* Rosetta DE3 cells were transformed with pEHISTEV::dΔhΔSCP-2L plasmid and plated overnight. A single colony of freshly transformed cells was cultured overnight in 50 ml LB media containing 50 µg/ml of kanamycin and 34 µg/ml of chloramphenicol at 37 °C, 200 rpm. Starter cultures (20 ml) were used for inoculation of 1000 ml of PB media (Production Broth medium; containing 20 g/L tryptone, 10 g/L yeast extract, 5 g/L dextrose, 5 g/L NaCl, 8.7 g/L K_2HPO_4, pH 7.0) supplemented with 50 µg/ml of kanamycin and 34 µg/ml of chloramphenicol and grown at 37 °C, 200 rpm to an OD_{600nm} ~ 0.6 (~ 2.5 h). Protein expression was induced with the addition of IPTG to a final concentration of 0.2 mM. The induced cultures were incubated for 18 h at 16 °C, 200 rpm.

For the expression of TAG mutants, chemically competent *E.coli* BL21 cells containing pEVOL-PylRSAlaBpy/tRNA^{CUA} were transformed with pET28_optimised_ΔSCP-2L plasmid plated overnight.

A single colony of freshly transformed cells was cultured overnight in 50 ml LB media containing 50 µg/ml of kanamycin and 34 µg/ml of chloramphenicol at 37 °C, 200 rpm. Starter cultures (20 ml) were used for inoculation of 1000 ml media supplemented with 50 µg/ml of kanamycin and 34 µg/ml of chloramphenicol and grown at 37 °C, 200 rpm to an OD_{600nm} ~ 0.6 (~ 2 h). Prior to induction of protein expression, racemic BpyAla was added with a final concentration of 0.5 mM and the temperature lowered to 30 °C. Protein expression was induced with the addition of IPTG with a final concentration of 0.5 mM and L-arabinose with a final concentration of 0.2% w/v. The induced cultures were incubated overnight 14 h at 30 °C, 200 rpm.

The cells of both induced cultures were subsequently harvested by centrifugation (4200 rpm for 15 min at 4 °C). Pelleted cells were resuspended in 20 ml PBS and pelleted by centrifugation (4200 rpm for 15 min at 4 °C), the buffer was discarded, and the cell pellets were frozen at -20 °C.

The purification protocol has been described in [1] and repeated here for clarity. After defrosting the cell pellets, 20 ml of lysis buffer (50 mM Tris-HCl, 20 mM imidazole, 150 mM NaCl, 0.5 mM benzamidine, pH 8) was added to resuspend the cells from 1 L of culture. Then 20 mg of lysozyme, 1 mg of DNase I, and 1 ml of 1 M $MgCl_2$ were added to the lysate and incubated for 1 h. The lysate was subjected to sonication (1 min, 90 % power with 5 s pulses). Cell lysates were cleared by centrifugation (4200 rpm for 1 h at 4 °C) and supernatant, filtered with 0.45 µm filter (Millipore, Merck), was subjected to IMAC 5 mL HisTrap HP column at a constant flow-rate of 5 ml/min equilibrated with 5 cv of wash buffer (30 mM Tris-HCl, 150 mM NaCl, pH 8 containing 20 mM imidazole). The column was first washed with 5-10 cv of wash buffer. The His-tagged variants were eluted with 6 cv of 50% elution buffer (wash buffer containing 330 mM imidazole). Eluted proteins were dialyzed against 5 L of buffer solution (wash buffer with 10 mM imidazole) at 4 °C overnight. To remove the His-tag, 0.014 equivalents of TEV-protease and final concentrations of 1 mM DTT and 0.5 mM EDTA were added and incubated for 6 h at room temperature. After TEV cleavage, the protein solution was subjected to IMAC 5 ml HisTrap HP column equilibrated with wash buffer at a constant flow of 5 ml/min. The flow-through that contained SCP-2L variants with His-tag cleaved was collected and analysed by SDS PAGE (RunBlue 4-12% Bis-Tris Gel, Expedeon; NuPAGE MES SDS Running Buffer, Life Technologies; Invitrogen Mark12™ unstained standard). The protein concentrations were determined using A_{280} and the extinction coefficients corrected for each mutant for the presence of BpyAla (ε=14000**)** (**Table S2**), the concentration of modified Cys mutants and TAG mutants was determined using the Mw and extinction coefficient.

Table S2: Extinction coefficients of the mutants.

Protein mutant	Extinction coefficient (ε) [M^{-1} cm^{-1}] at 280 nm
**A100C**	13,980
**Q111C**	13,980
**V83C**	13,980
**A100CBpy**	28,680
**Q111CBpy**	28,680
**V83CBpy**	28,680
**A100BpyAla**	30,170
**V83BpyAla**	30,170
**Q111BpyAla**	30,170

3.1 SDS PAGE of protein purification

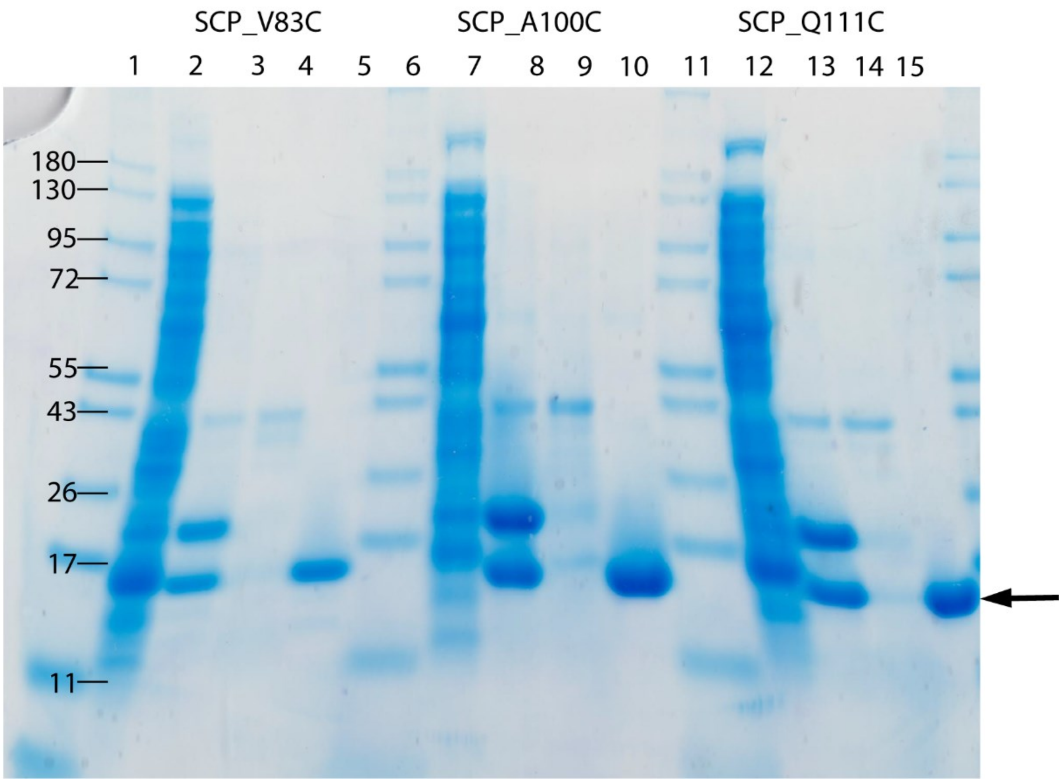

Figure S1: SDS PAGE purity analysis of SCP_2L purifications. **SCP_V83C**: **1** Ladder, **2** Flow-through of the first Ni-column purification, **3** After first Ni-column purification and dialysis, **4** Flowthrough of the second Ni-column purification, **5** Pure protein after second Ni-column purification, **SCP_Q111C**: **6** Ladder, **7** Flow-through of the first Ni-column purification, **8** After first

Ni-column purification and dialysis, **9** Flowthrough of the second Ni-column purification, **10** Pure protein after second Ni-column purification, **SCP_A100C**: **11** Ladder, **12** Flow-through of the first Ni-column purification, **13** After first Ni-column purification and dialysis, **14** Flowthrough of the second Ni-column purification, **15** Pure protein after second Ni-column purification.

3.2 ESI-mass spectra of SCP mutants

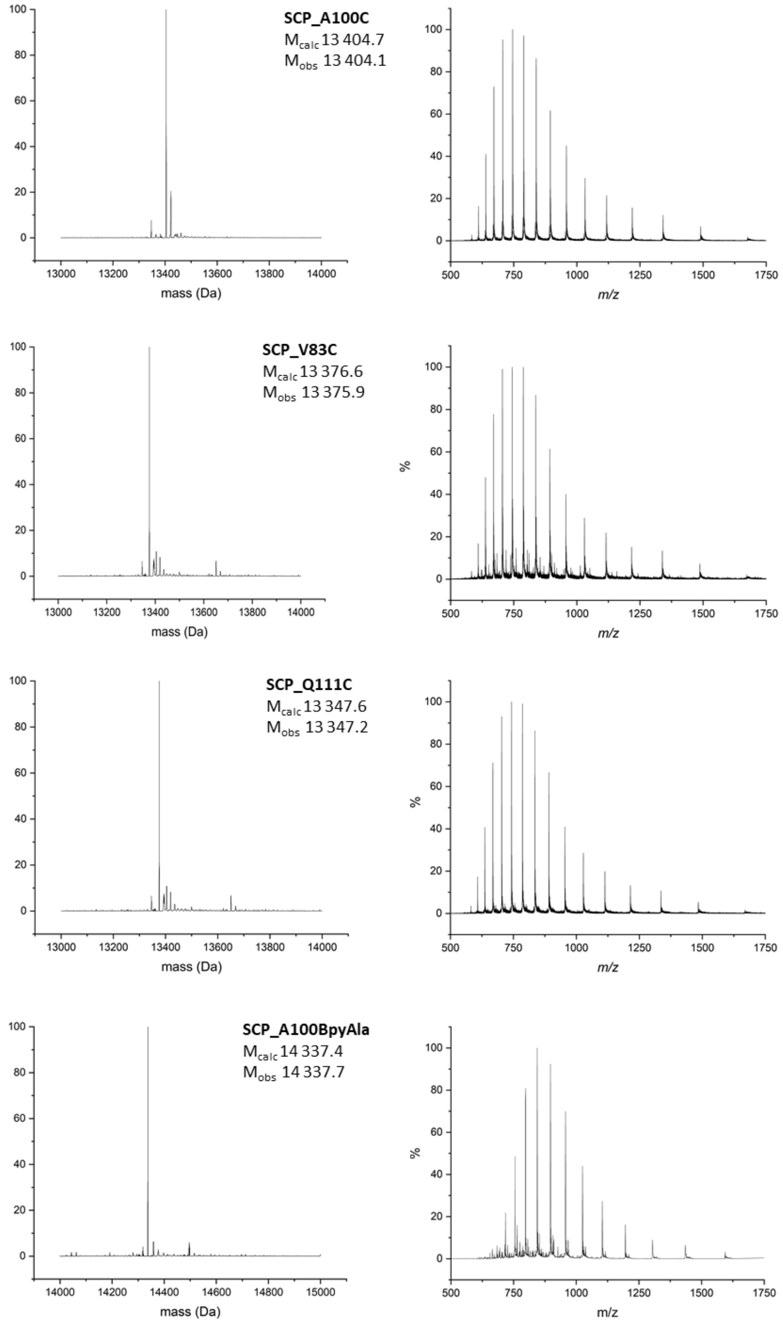

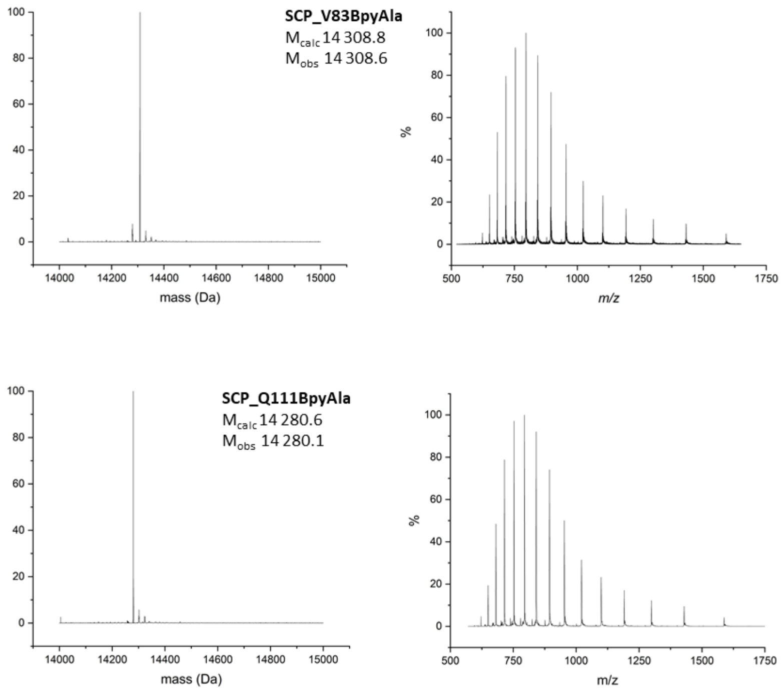

Figure S2: Deconvoluted and raw spectra, high-resolution mass spectroscopy for all protein mutants after purification used in this study. Comparison of calculated (M_{calc}) and observed (M_{obs}) masses confirms the identity of each variant and successful incorporation of Cys and BpyAla. For the analysis of data we used MassLynx software.

4 Scanning of the Bioconjugation Conditions

Purified protein samples of Cys mutants (following 2.2.2) were buffer-exchanged to HEPES (0.1 M, 50 mM NaCl, pH 8.5) with Amicon 10kDa Ultra Centrifugal Filters (Merck, USA). For the screening, reaction volumes of 25 µL with the protein concentration of 71.5 µM were used. The following sets of conditions were screened: pH 8 and 8.5; 5 and 10 equivalents of Br-Bpy (10 mM in DMSO/H_2O). The bioconjugation reaction was left for 1 h at room temperature and was stopped with 10-fold dilution with H_2O. The optimal conditions were pH 8.5 and 10 equivalents of Br-Bpy **1**. The percentage of incorporation was determined using ESI-MS, comparing % abundance of modified and unmodified protein on the most abundant charged state.

5 Bioconjugation of Cys Mutants

Purified protein samples of Cys mutants were buffer-exchanged into HEPES (0.1 M, 50 mM NaCl, pH 8.5) and diluted to the concentration of 100 µM. To the protein solution, 10 equivalents of the Br-Bpy complex were added and the bioconjugation reaction took place for 1 h at 25 °C. The reaction was stopped with buffer exchange into MES buffer (20 mM, 50 mM NaCl, pH 6) using Amicon 10kDa Ultra Centrifugation Filters (Merck, USA). The bioconjugation of Cys mutants was confirmed using ESI-MS analysis. The protein identity further analysed with SDS PAGE.

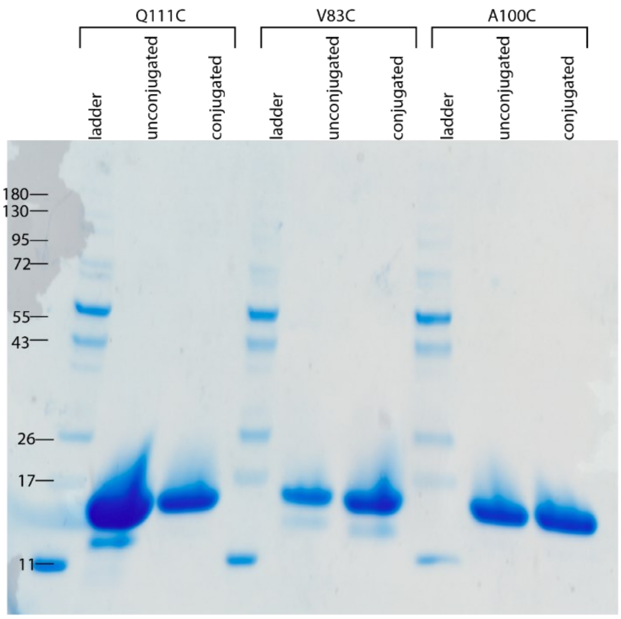

Figure S3: SDS PAGE analysis of the Cys mutants, non-modified and modified (conjugated with Br-Bpy) (Invitrogen Mark12^{TM} unstained standard).

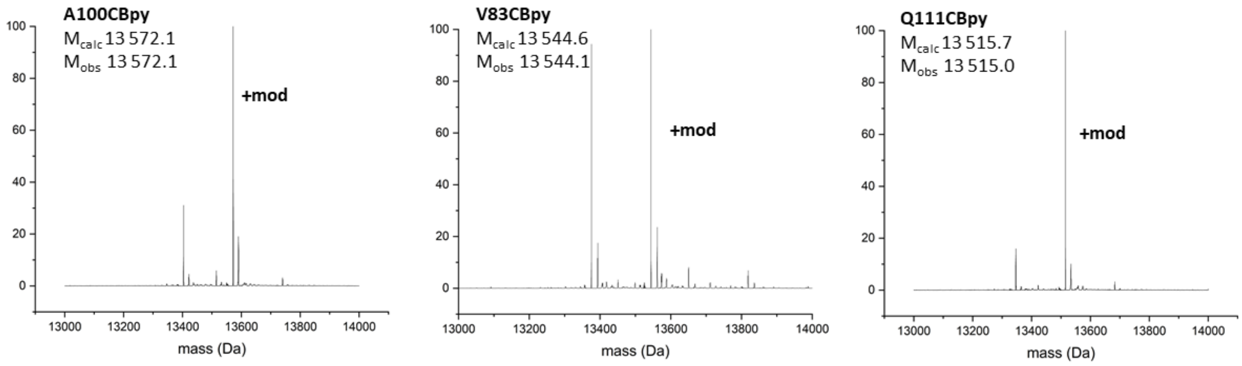

Figure S4: Deconvoluted, high-resolution mass spectroscopy for all protein mutants after modification with Br-Bpy used in this study. Comparison of calculated (M_{calc}) and observed (M_{obs}) masses confirms the identity of each variant and successful Cys modification.

6 Characterization of Cu(II) Binding to Conjugated Cys Mutants and TAG Mutants

6.1 UV-Vis

Absorption spectra (250-450 nm) were recorded for metal titration against proteins containing the Bpy metal-binding moiety (1.5 mL reaction with the protein concentration of 20 µM in MES buffer) and 10 mM stock solution of Cu(II) in dH_2O at room temperature. Prior to addition of Cu(II) ions, protein samples were filtered (0.22 µm) and cleared by centrifugation (9,000× g, 10 min). The protein spectra without Cu(II) ions were measured as a reference. The 1.5 mL protein solution was titrated with stepwise increase of 5 µM of $Cu(NO_3)_2$ (0.75 µL), the solution was incubated for 10 min at room temperature before the absorbance scan. The concentration of $Cu(NO_3)_2$ was increased until no differences in the absorption spectra were observed between two subsequent concentrations.

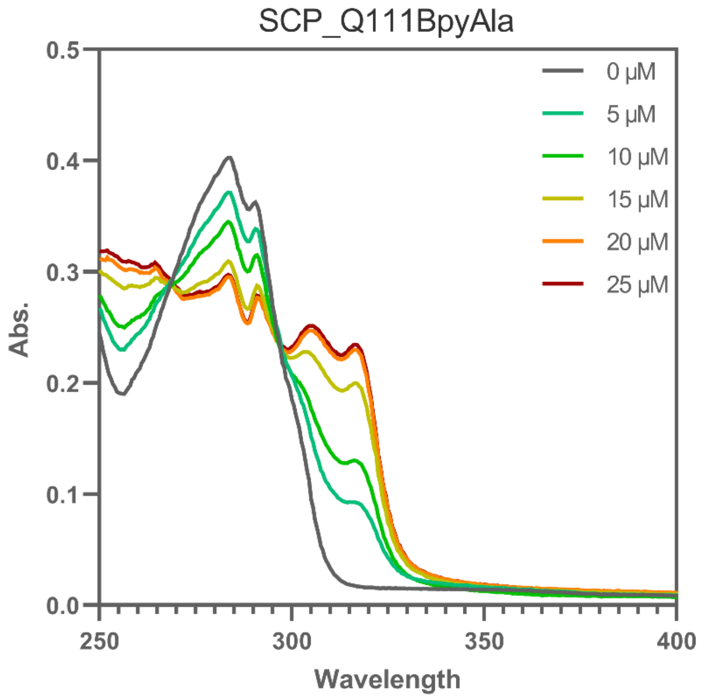

Figure S5: Absorbance scan of SCP_Q111BpyAla (20 µM) titration with $Cu(NO_3)_2$.

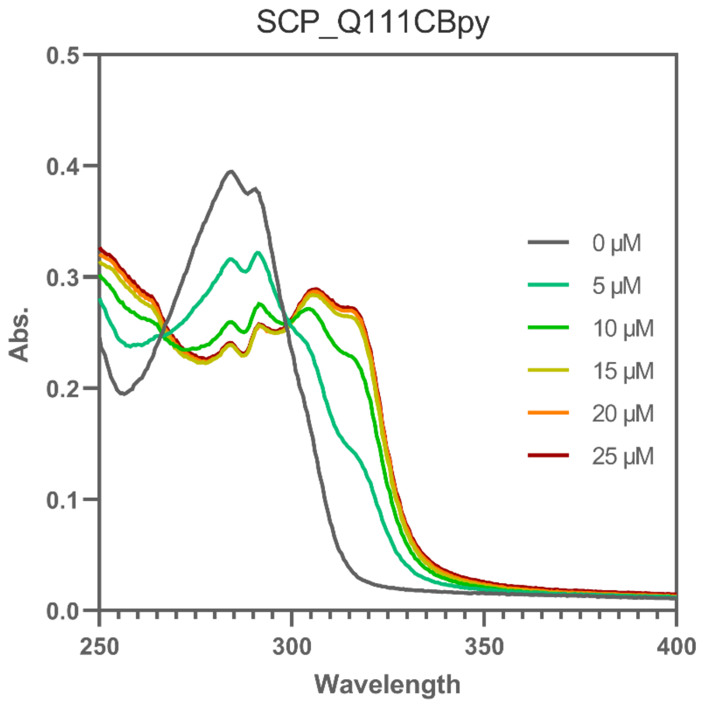

Figure S6: Absorbance scan of SCP_Q111CBpy(20 µM) titration with $Cu(NO_3)_2$.

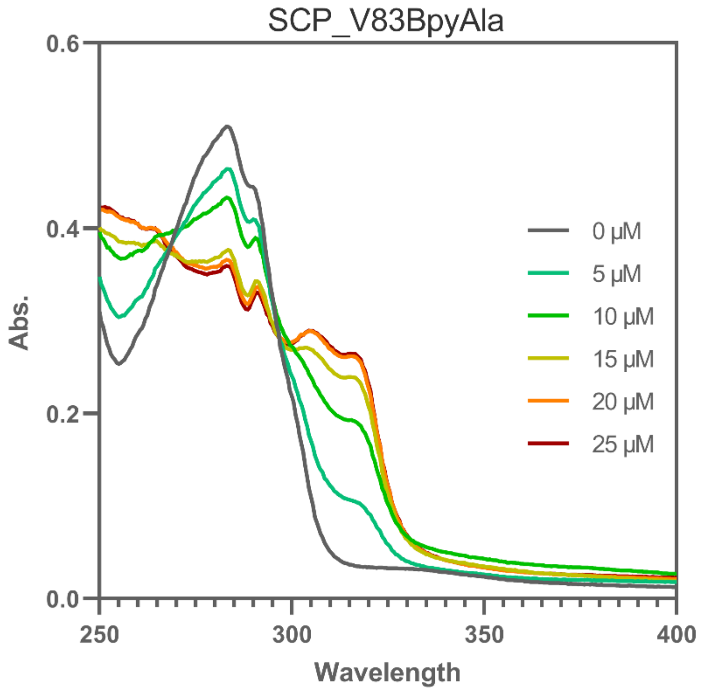

Figure S7: Absorbance scan of SCP_V83BpyAla (20 µM) titration with $Cu(NO_3)_2$.

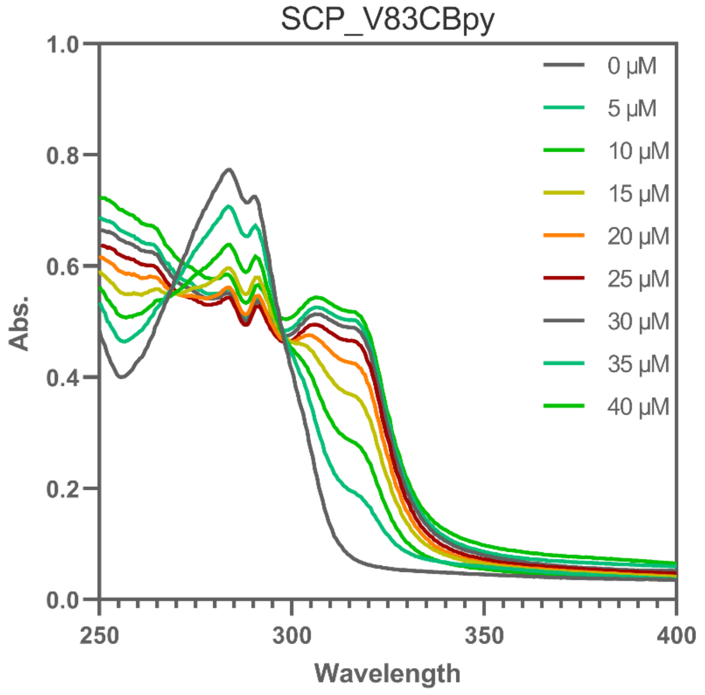

Figure S8: Absorbance scan of SCP_V83CBpy(20 μM) titration with $Cu(NO_3)_2$.

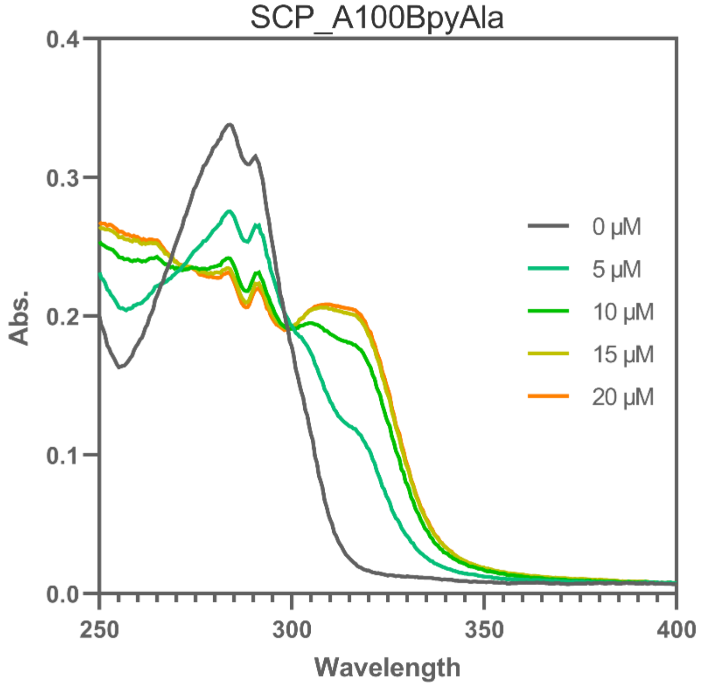

Figure S9: Absorbance scan of SCP_A100BpyAla (20 μM) titration with $Cu(NO_3)_2$.

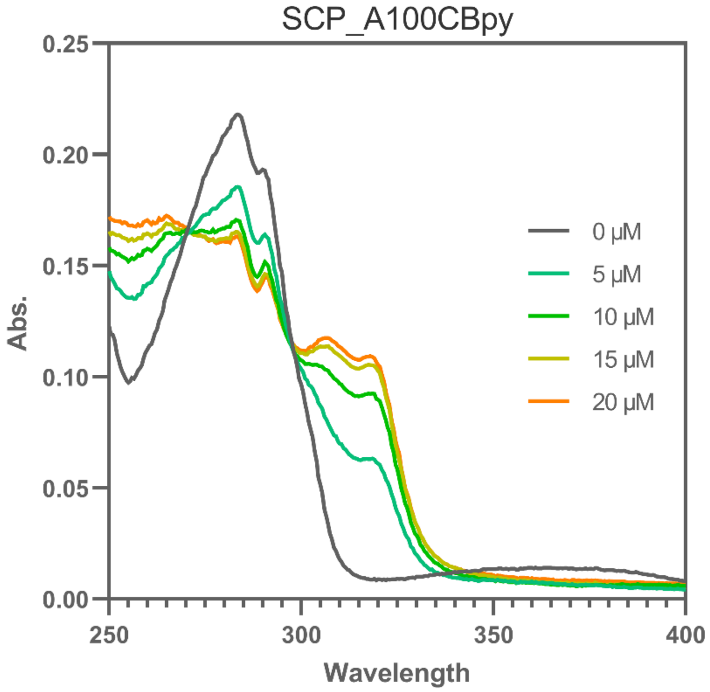

Figure S10: Absorbance scan of SCP_A100CBpy (20 µM) titration with $Cu(NO_3)_2$.

6.2 ICP analysis

ICP analysis was used to accurately determine the copper concentrations in samples to determine the stoichiometry of Cu binding to the Bpy moiety in the protein. SCP_Bpy conjugates in MES buffer (20 µM, determined using A_{280} and the extinction coefficients given in Table S3) were incubated with one equivalent of metal salt in order to form the 1:1 complex SCP_2L:Cu^{2+} and then buffer exchanged three-times to remove any residual copper using Amicon 10kDa Ultra Centrifucation filters (Merck), never exceeding the initial volume. Duplicate samples for ICP analysis were prepared as following: to 100 µL of the resulting metalloproteins soultion in a 15 ml falcon tube was added nitric acid (100 µL, 69% Aristar for trace analysis) and heated at 70 °C for 5-6 h. It was diluted to 3 ml to give concentration of less than 5% of nitric acid.

Trace metal analysis was performed by the microanalysis service at the University of Edinburgh on an Agilent 7500ce (with octopole reaction system), employing an RF forward power of 1540 W and reflected power of 1 W, with argon gas flows of 0.81 L min^{-1} and 0.20 L min^{-1} for carrier and makeup flows, respectively. Sample solutions were taken up into the Micro mist nebuliser by peristaltic pump at a rate of approximately 1.2 mL min-1. Skimmer and sample cones were made of nickel. The instrument was operated in spectrum multi-tune acquisition mode and three replicate runs per sample were employed.

Table S3: ICP analysis of Cu binding to SCP_Q111BpyAla

	Expected concentration of Cu (in µM)	Concentration of Cu (in µM)
Coupling buffer (MES)	0	0.41
20 µM Cu in coupling buffer	20	20.26
Cu 1:1 **Q111BpyAla**	20	13.49

7 Friedel-Crafts Alkylation

The biocatalysis was carried out as described in [3]. The biocatalytic reaction was prepared by combining $Cu(NO_3)_2{\cdot}3H_2O$ (90 µM in H_2O) in coupling MES buffer with 1.25 equivalents of SCP-2L containing Bpy (conjugated Cys mutants or TAG mutants) (112.5 µM) with a final volume of 280 µL. To this 10 µL of a fresh stock solution of **3** (indole, 70 mM in MeCN, final concentration of 2.5 mM) and **4** (1-(1-methyl-1H-imidazol-2-yl)but-2-en-1-one, 28 mM in MeCN/H_2O final concentration of 1 mM). The reaction was incubated at 4°C for 72 h, after which 10 µL of 2-phenyl quinoline(4.9 mM in 1:1 IPA:hexane) was added. The product was extracted using 3 x 1 mL diethyl ether, the combined organic layers dried over Na_2SO_4 and evaporated under reduced pressure. The product was dissolved in propan-2-ol (150 µL) and the yield and enantiomeric excess (e.e.) were determined using HPLC.

Yields of the catalytic reactions are based on peak areas 280 nm using 2-phenylquinoline as internal standard.

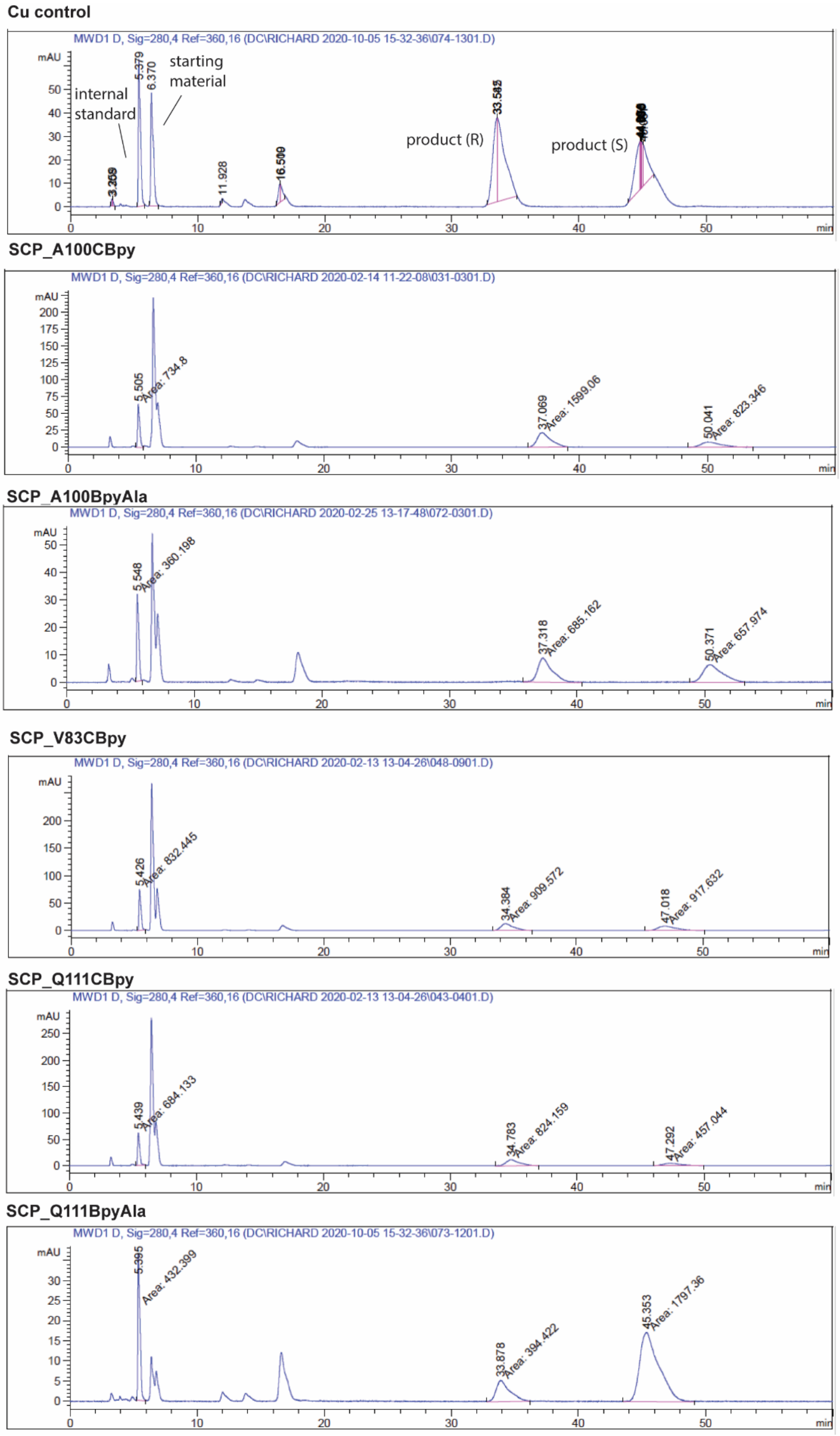

Figure S11: HPLC traces.

7.1 pH effect on enantioselecitivity

Table S4: pH effect on enantioselectivity and yield of F-C reaction.

Entry	Catalyst	Buffer	yield of 5 (%)[a]	e.r.[b] (R:S)
1	$Cu(NO_3)_2$ (control)	MES, pH 6	76	50:50
2	$Cu(NO_3)_2$	MOPS, pH 7	49	50:50
3	$Cu(NO_3)_2$	MES, pH 5	98	50:50
4	SCP_Q111BpyAla (control)	MES, pH 6	42 (±7)	20:80 (±1)
5	SCP_Q111BpyAla	MOPS, pH 7	17 (±1)	23:77 (±3)
6	SCP_Q111BpyAla	MES, pH 5	55 (±9)	17:83 (±1)

8 Alanine scan

The mutations were prepared using site-directed mutagenesis as described (***2.3 Site directed mutagenesis***).

Table S5: Primers used to make SCP_Q111BpyAla Alanine mutants.

Entry	Primer	Sequence
**1**	Q108A **_Fw**	TGCTGAGCGCGAAACTGTAGATGATTCTGAAGGATTATGC
**2**	Q108A**_Rv**	CAGTTTCGCGCTCAGCATAATGTTGCCACGTGCTTTCAG
**3**	M112A**_Fw**	AACTGTAGGCGATTCTGAAGGATTATGCGAAGCTGGGATC
**4**	M112A**_Rv**	TTCAGAATCGCCTACAGTTTTTGGCTCAGCATAATGTTGCC
**5**	D88A**_Fw**	AAGCTGGCGCCGCAAAAAGCGTTCTTTAGCGGTCGTC
**6**	D88A**_Rv**	TTTTGCGGCGCCAGCTTGCCCAGAACCACTTCCATAAA
**7**	Q90A**_Fw**	GGACCCGGCGAAAGCGTTCTTTAGCGGTCGTCTGAAAG
**8**	Q90A**_Rv**	CGCTTTCGCCGGGTCCAGCTTGCCCAGAACCACTTC
**9**	F34A**_Fw**	GCGGTGGCGGAGTGGCACATCACCAAGGGTGGCAA
**10**	F34A**_Rv**	CCACTCCGCCACCGCGTTAACTTTCTTAACCACTTCCG
**11**	V82A**_Fw**	TTTATGGAAGCAGTTCTGGGCAAGCTGGACCCGCAAAA
**12**	V82A**_Rv**	CAGAACTGCTTCCATAAAATCCTCGTCGCTCAGAATGAT
**13**	K115A**_Fw**	GCATAATCTGCCAGAATCATCTACAGTTTTTGGCTCAGCAT
**14**	K115A**_Rv**	TGATTCTGGCAGATTATGCGAAGCTGGGATCCGAAAACC
**15**	L114A**_Fw**	AGATGATTGCGAAGGATTATGCGAAGCTGGGATCCGAAAA
**16**	L114A**_Rv**	ATAATCCTTCGCAATCATCTACAGTTTTTGGCTCAGCATAATG

The following mutants were expressed and purified: Q111Bpy (for the control), Q111BpyAla_F34A, Q111BpyAla_L114A, Q111BpyAla_M112A, Q111BpyAla_Q108A, Q111BpyAla_D88A, Q111BpyAla_Q90A, Q111BpyAla_V82A, Q111BpyAla_K115A as described in ***3 Protein expression and purification***.

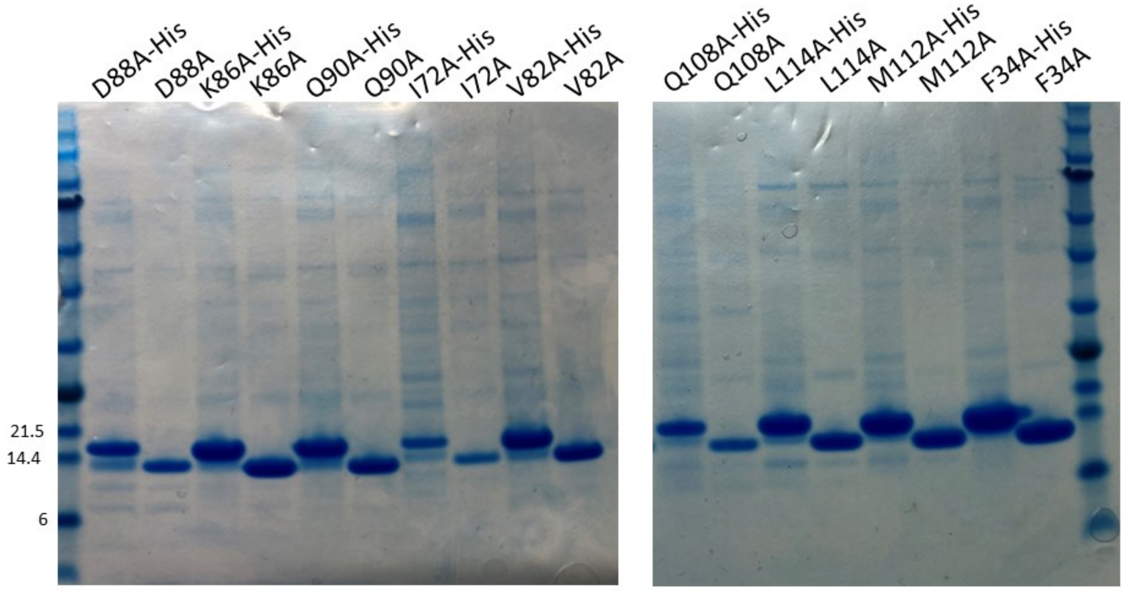

Figure S12: SDS PAGE analysis.

8. 1 Protein Sequences of SCP_Q111BpyAla Ala mutants

Q111BpyAla = X

>SCP_Q111BpyAla

MEGGKLQSTFVFEEIGRRLKDIGPEVVKKVNAVFEWHITKGGNIGAKWTIDLKSGSGKVYQG
PAKGAADTTIILSDEDFMEVVLGKLDPQKAFFSGRLKARGNIMLSQKLXMILKDYAKLGSEN
LYFQ

>SCP_Q111Bpy_D88A

MEGGKLQSTFVFEEIGRRLKDIGPEVVKKVNAVFEWHITKGGNIGAKWTIDLKSGSGKVYQG
PAKGAADTTIILSDEDFMEVVLGKLAPQKAFFSGRLKARGNIMLSQKLXMILKDYAKLGSEN
LYFQ

>SCP_Q111Bpy_F34A

MEGGKLQSTFVFEEIGRRLKDIGPEVVKKVNAVAEWHITKGGNIGAKWTIDLKSGSGKVYQ
GPAKGAADTTIILSDEDFMEVVLGKLDPQKAFFSGRLKARGNIMLSQKLXMILKDYAKLGSE
NLYFQ

>SCP_Q111Bpy_L114A

MEGGKLQSTFVFEEIGRRLKDIGPEVVKKVNAVFEWHITKGGNIGAKWTIDLKSGSGKVYQG
PAKGAADTTIILSDEDFMEVVLGKLDPQKAFFSGRLKARGNIMLSQKLXMIAKDYAKLGSEN
LYFQ

>SCP_Q111Bpy_V82A

MEGGKLQSTFVFEEIGRRLKDIGPEVVKKVNAVFEWHITKGGNIGAKWTIDLKSGSGKVYQG
PAKGAADTTIILSDEDFMEAVLGKLDPQKAFFSGRLKARGNIMLSQKLXMILKDYAKLGSEN
LYFQ

>SCP_Q111Bpy_I72A
MEGGKLQSTFVFEEIGRRLKDIGPEVVKKVNAVFEWHITKGGNIGAKWTIDLKSGSGKVYQG
PAKGAADTTAILSDEDFMEVVLGKLDPQKAFFSGRLKARGNIMLSQKLXMILKDYAKLGSE
NLYFQ
>SCP_Q111Bpy_M112A
MEGGKLQSTFVFEEIGRRLKDIGPEVVKKVNAVFEWHITKGGNIGAKWTIDLKSGSGKVYQG
PAKGAADTTIILSDEDFMEVVLGKLDPQKAFFSGRLKARGNIMLSQKLXMILKDYAKLGSEN
LYFQ
>SCP_Q111Bpy_Q90A
MEGGKLQSTFVFEEIGRRLKDIGPEVVKKVNAVFEWHITKGGNIGAKWTIDLKSGSGKVYQG
PAKGAADTTIILSDEDFMEVVLGKLDPAKAFFSGRLKARGNIMLSQKLXMILKDYAKLGSEN
LYFQ
>SCP_Q108A
MEGGKLQSTFVFEEIGRRLKDIGPEVVKKVNAVFEWHITKGGNIGAKWTIDLKSGSGKVYQG
PAKGAADTTIILSDEDFMEVVLGKLDPQKAFFSGRLKARGNIMLSAKLXMILKDYAKLGSEN
LYFQ
>SCP_K115A
MEGGKLQSTFVFEEIGRRLKDIGPEVVKKVNAVFEWHITKGGNIGAKWTIDLKSGSGKVYQG
PAKGAADTTIILSDEDFMEVVLGKLDPQKAFFSGRLKARGNIMLSQKLXMILADYAKLGSEN
LYFQ

9 Size exclusion chromatography of SCP_Q111BpyAla

Prior to the crystallization screen the proteins underwent a polishing purification step using gel filtration chromatography. The protein sample was concentrated to a volume of ~ 1 ml. The concentrated sample was loaded onto a HiPrep Superdex S 75 HR column (Vt ~ 120 ml, 2.6 x 60 cm) (GE Healthcare, Uppsala, Sweden) pre-equilibrated with size-exclusion buffer (20 mM PIPES, 1 mM EDTA, 1 mM sodium azide, 150 mM NaCl, pH 7.5).

10 Crystallization

Prior to crystallisation, proteins were concentrated by centrifugal filtration in buffer containing 20 mM PIPES, 1 mM EDTA, 1 mM sodium azide, 150 mM NaCl, pH 7.5, 0.15 mM Triton X-100 to 5 mg/ml (SCP_Q111CBpy; 120 amino acids) and 5 mg/ml (SCP_Q111BpyAla; 128 amino acids). Crystals of SCP_Q111Cbpy were grown by hanging-drop vapour diffusion at 4 °C using the same precipitant used to grow crystals of the wt SCP_2L [16]: 2.3M ammonium sulphate, 100 mM citric acid pH 5.9 and 200 mM NaCl. Drops contained 1 μL of SCP_Q111CBpy protein and 1 μL of precipitant solution. Crystals with approximate dimensions 0.05 mm x 0.05 x 0.05 mm grew within 14 days. SCP_Q111BpyAla did not spontaneously crystallise in these or similar conditions, so a few SCP_Q111CBpy crystals were

crushed and extracted to form a stock seed solution for streak seeding of SCP_Q111BpyAla-containing drops using a cat's whisker. After streak seeding, crystals of SCP_Q111BpyAla appeared after 30 days. To incorporate Cu(II) ions, crystals were transferred to a drop containing precipitant plus 10 mM $Cu(NO_3)_2$ and incubated over precipitant for 3 days. Then, prior to diffraction, crystals were briefly transferred to a cryoprotectant solution containing precipitant and 25% (w/v) sucrose and flash frozen in liquid nitrogen.

The modified Q111CBpy was crystalized as described above. The Cu(II) binding to the protein crystal was achieved by soaking the crystal in 10 mM $Cu(NO_3)_2$ solution for ~3 days. Sucrose was left to diffuse for 30 min before the crystal was flash frozen at 100 K.

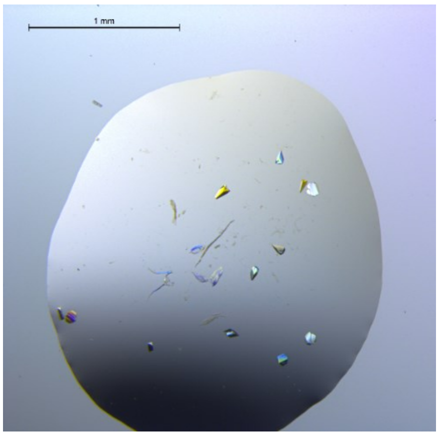

Figure S13: SCP_Q111CBpy crystals

10. 1 X-ray crystal structure determination and refinement

X-ray diffraction data were collected on beam line I04 or I03 at the Diamond Light Source. The wavelength of the X-rays was 1.378 Å and 0.9795 Å for copper-soaked crystals of SCP_Q111CBpy and SCP_Q111BpyAla, respectively. Copper-soaked SCP_Q111CBpy crystals displayed orthorhombic (P212121) symmetry, with one protein molecule in the asymmetric unit, and diffracted X-rays to a maximum resolution of 1.52 Å. The presence of copper in the crystals was confirmed by a fluorescence scan around the Cu-K edge. By contrast, the copper-soaked SCP_Q111BpyAla crystals formed in the P21 space group (with two SCP molecules in the asymmetric unit) and diffracted X-rays to 2.52 Å. X-ray diffraction data were processed with xia2 or autoPROC and the statistics are shown in Table 2. Initial phases were determined by molecular replacement, using the structure of wt SCP_2L (PDB ID: 1IKT) as the search model in PHASER. The structures was refined with phenix.refine and Coot. The refinement statistics are shown in Table 2. All structural diagrams were prepared using PyMOL (http://www.pymol.org/) and Adobe Illustrator.

Table S6 X-ray diffraction Data collection and refinement statistics.

Statistics for the highest-resolution shell are shown in parentheses

Crystal	SCP_Q111CBpy_Cu	SCP_Q111Bipy_Cu
PDB ID	8AF3	8AF2
Beamline	I03	I04
Wavelength (Å)	1.378	0.9795
Resolution (Å)	39.68 - 1.52 (1.574 - 1.52)	62.206-2.514 (2.604 - 2.514)
Space group	$P\ 2_1\ 2_1\ 2_1$	$P1\ 2_1\ 1$
Unit cell dimensions (a,b,c, α,β,γ)	35.16 51.05 63.06<br>90 90 90	34.51 50.52 62.21<br>90.00 90.73 90.00
Total reflections	198755(3469)	38673 (1912)
Unique reflections	17614 (1005)	7367 (716)
Multiplicity	11.3 (5.3)	5.2 (5.2)
Completeness (%)	97.14 (78.79)	99.4 (99.5)
Mean I/sigma(I)	26.4 (1.0)	6.6 (1.1)
Wilson B-factor	32.77	47.02
R-merge		0.182 (1.503)
R-meas	0.044 (1.711)	0.228 (1.88)
CC1/2	1.0 (0.4)	0.996 (0.352)
Reflections used in refinement	17566 (1389)	7365 (717)
Reflections used for R-free	847 (74)	333 (34)
R-work	0.229 (0.406)	0.234 (0.330)
R-free	0.238 (0.457)	0.304 (0.432)
Number of non-hydrogen atoms	1035	2052
macromolecules	902	1944
ligands	80	195
solvent	53	21
Protein residues	116	1-128
RMS(bonds)	0.019	0.004
RMS(angles)	2.05	0.58
Ramachandran favoured (%)	97.3	94.87
Ramachandran allowed (%)	2.3	5.13
Ramachandran outliers (%)	0	0
Rotamer outliers (%)	0	1.49
Clashscore	3.34	12.36

Average B-factor (Å^2)	75.34	50.4
macromolecules	71.63	49.95
ligands	125.07	59.67
solvent	63.43	53.30
Anomalous completeness	96.5 (70.9)	
Anom multiplicity	6.1 (2.8)	
cc (anom)	0.4	
f'	-8.2	
f''	3.4	

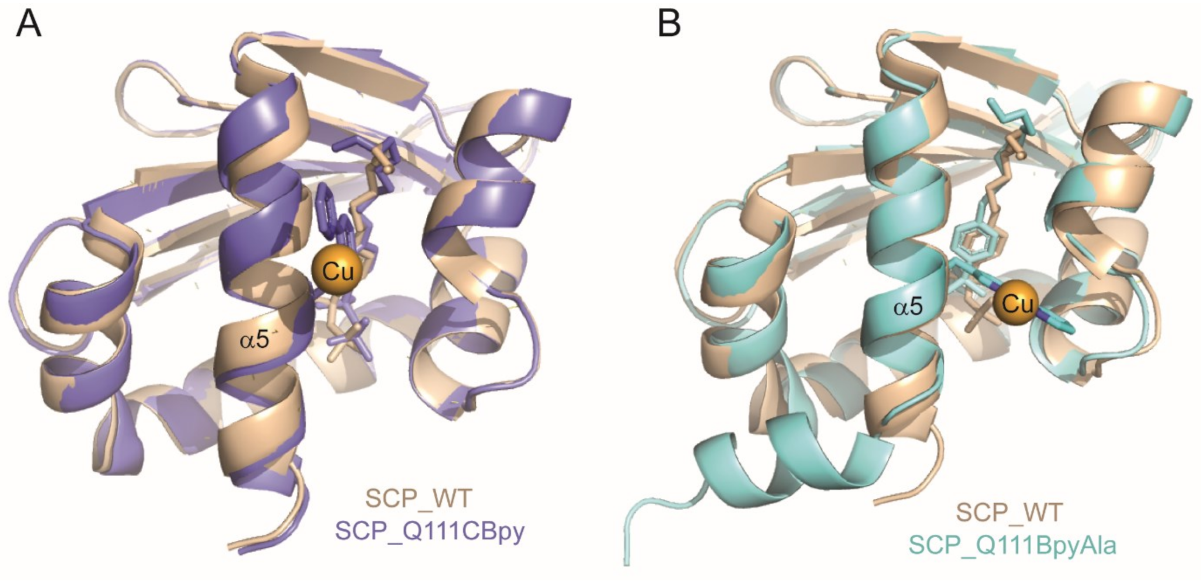

Figure S14: Overlay of the structures of wild-type SCP (PDB ID: 1IKT) with the structures of (A) SCP_Q111CBpy (PDB ID: 8AF3) and (B) SCP_Q111BpyAla PDB ID: 8AF2).

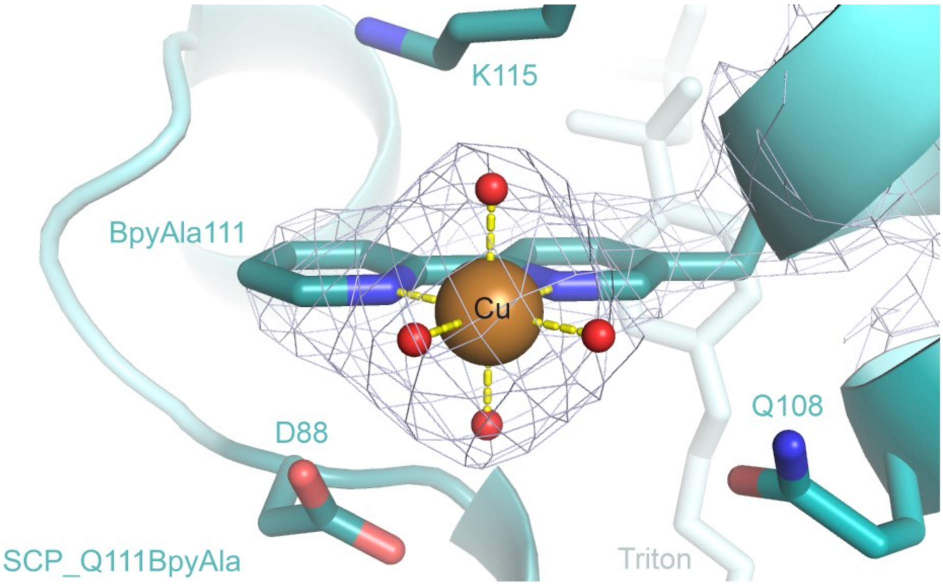

Figure S15: Close-up view of Cu(II) bound to SCP_Q111BpyAla, showing octahedral coordination of the four water molecules (red spheres) and the N1 and N2 of BpyAla111. The grey mesh represents the $2F_o$-F_c electron density map (contoured at 1σ).

11 Synthesis of BpyAla

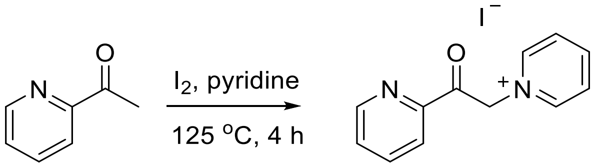

1-(2-pyrdylacetyl)pyridinium iodide, S1

Anhydrous pyridine (400 mL) was added to iodine (75.6 g, 300 mmol) under Ar atmosphere. Acetyl pyridine (33.6 mL, 300 mmol) was added, the solution was heated to 125 ºC for 4 h. The heating was then turned off and the solution was stirred at r.t. as it cooled overnight. The suspension was filtered under vacuum,washed with EtOH (100 mL, then 50 mL) and dried over vacuum giving the crude product **S1** as a black solid (120.8 g, qt. yield, **Mp** 196-197 °C); Analytical data in accordance with literature [4]. **^{1}H NMR** (500 MHz, DMSO-*d6*) δ 9.02 (2H, dt, *J* = 5.4, 1.7, 1.2 Hz), 8.88 (1H, dt, *J* = 4.7, 1.3 Hz), 8.74 (1H, tt, *J* = 7.9, 1.4 Hz), 8.29 (2H, dd, *J* = 7.9, 6.5 Hz), 8.15 (1H, td, *J* = 7.7, 1.7 Hz), 8.08 (1H, dt, *J* = 7.9, 1.2 Hz), 7.84 (1H, ddd, *J* = 7.5, 4.7, 1.3 Hz), 6.52 (2H, s). **^{13}C NMR** (126 MHz, DMSO-*d6*) δ 191.46, 150.45, 149.55, 146.35, 146.29, 138.14, 129.13, 127.71, 122.03, 66.64.

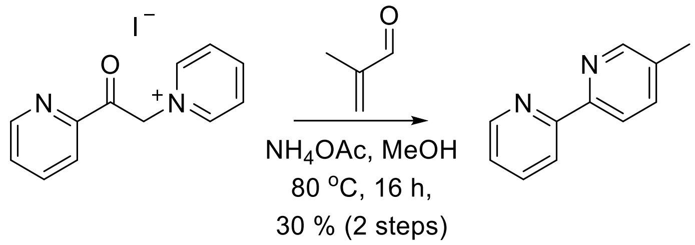

5-methyl-2,2'-bipyridine*, *S2

S1 (115 g, 300 mmol assuming qt. yield from 1st step) was dissolved in MeOH (500 mL) and NH_4OAc (44.6g, 577.5 mmol) and heated to 80 °C. Methacrolein (17.5 ml, 212 mmol) was added via syringe and the reaction heated at 80 °C for 16 h. The solution was allowed to cool the stirring was stopped for the precipitate to settle. The solvent was decanted into a new flask and concentrated to approximately ¼ of the volume). H_2O (200 mL) was added and the solution transferred to a separating funnel. The aqueous phase was extracted with n-hexane (4 x 200 mL) and the combined organics were dried over MgSO4 and concentrated giving **S2** as a yellow oil (15 g, 30% yield over 2 steps). Analytical data in accordance with literature [5]. **^{1}H NMR** δ (500 MHz, $CDCl_3$) 8.78 (1H, dd, J = 4.7, 1.6 Hz), 8.62 (1H, d, J = 2.6 Hz), 8.54 (1H, dd, J = 8.0, 1.3 Hz), 8.45 (1H, d, J = 8.1 Hz), 7.85 (1H, td, J = 7.6, 1.7 Hz), 7.66 (1H, dd, J = 8.2, 2.4 Hz), 7.30-7.43 (1H, m), 2.40 (3H, s). **^{13}C NMR** (126 MHz, $CDCl_3$) δ 156.44, 153.78, 149.77, 149.25, 137.59, 136.99, 133.54, 123.50, 120.92, 120.73, 18.48. **HRMS** $C_{11}H_{10}N_2$ $[M+H]^+$ m/z calculated for $C_{11}H_{11}N_2$ 171.0922, found 171.0851.

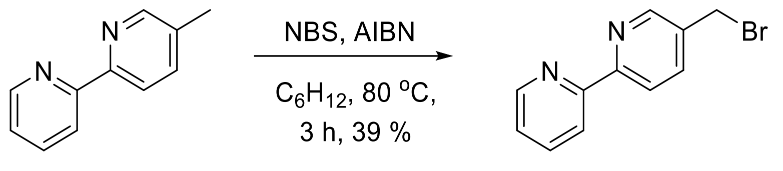

5-bromomethyl-2,2-bipyridine, S3

Cyclohexane (400 mL) was added to 5-methyl-2,2-bipyridine **S2** (15 g, 88.2 mmol) and degassed with Ar. NBS (29.9 g, 168 mmol, 1.75 eq, recrystalised from MeCN) was added. The solution heated to 80 °C with vigorous stirring (1000 rpm). AIBN (790 mg, 4.8 mmol, 0.05 eq) was added portionwise over about 20 minutes and heated for 3 h further causing a black precipitate to form. The solution was allowed to cool slightly and the solution was decanted away from the black precipitate, the precipitate was rinsed with 2x30 mL hot cyclohexane and then concentrated on the rotovap to give a yellow/brown oil. The flask was put in a ice bath whit stirring and cold hexane (50 ml) was added in one portion . The pale yellow precipitate was collected by vacuum filtration, washed with cold hexane (2 x 20 mL) and dried over vacuum giving the product **S3** as a pale yellow solid (12.9 g, 59 % yield). Analytical data in accordance with literature [6]. **^{1}H NMR** δ (500 MHz, $CDCl_3$) 8.70 (1H, s), 8.42 (1H, br d, J = 8.2 Hz),

7.88 (1H, dd, *J* = 8.2, 2.4 Hz), 7.85 (1H, td, *J* = 7.7, 1.9 Hz), 7.34 (1H, ddd, *J* = 7.5, 4.8, 1.2 Hz), 4.56 (2H, s). **^{13}C NMR** (126 MHz, $CDCl_3$) δ 156.15, 155.63, 149.46, 149.35, 137.75, 137.20, 133.82, 124.12, 121.43, 121.21, 29.76. **HRMS** $C_{11}H_9BrN_2$ $[M+H]^+$ m/z calculated for $C_{11}H_{10}BrN_2$ 249.0027, found 248.9833.

Diethyl 2-(2,2′-bipyridin-5-ylmethyl)-2-acetamidomalonate, S4

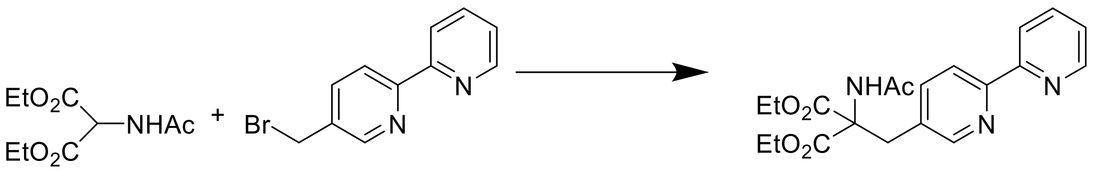

To a 2-neck 250 mL flask containing NaH (2.2 g, 55 mmol, 60% in mineral oil) under argon at 0 ºC was added anhydrous DMF (10 mL). Diethylacetamidomalonate (10.8 g, 50 mmol, 1 eq.) was dissolved in anhydrous DMF (20 mL) and added to the NaH solution dropwise with stirring. As the addition neared completion, the solution became viscous and stirring slowed. The cooling bath was removed causing the solution to become fluid once more. Addition of the malonate was continued ensuring the temperature of the reaction did not go above 20 ºC. After addition was complete the solution was stirred at r.t. for 30 min.

The solution was cooled back to 0 ºC on ice. Bromomethy-2,2-bipyridine **S3** (12.35 g, 50 mmol) was dissolved in DMF (10 mL) and added dropwise to the malonate solution. After addition the reaction was stirred at r.t. for 5 h. The solution was cooled back to 0 ºC on ice and water (150 mL) was added with vigorous stirring. The precipitate that formed was collected by vacuum filtration. The solid was taken up into $CHCl_3$ (50 mL) and stirred with activated charcoal (approx. 2.5 g). The solution was filtered through celite and concentrated under vacuum oven giving **S4** as a white solid (11.6 g, 60% yield). Analytical data in accordance with literature [5]. **^{1}H-NMR** δ (400 MHz, $CDCl_3$): 8.70 (1H, ddd, *J* = 4.9, 1.8, 0.9 Hz), 8.40-8.34 (3H, m), 7.85 (1H, td, *J* = 7.8, 1.8 Hz), 7.51 (1H, dd, *J* = 8.1, 2.3 Hz), 7.34 (1H, ddd, *J* = 7.5, 4.9, 1.2 Hz), 6.64 (1H, s), 4.19-4.21 (4H, m), 3.73 (2H, s), 2.06 (3H, s),1.30 (6H, t, *J* = 8.0 Hz). **^{13}C NMR** (126 MHz, $CDCl_3$) δ 169.53, 167.31, 150.28, 149.25, 138.65, 137.28, 131.39, 123.96, 121.33, 120.96, 67.17, 63.12, 35.20, 23.25, 14.20. **HRMS** $C_{20}H_{23}N_3O_5$ $[M+H]^+$ m/z calculated for $C_{20}H_{24}N_3O_5$ 386.1716, found 386.1220.

(2,2′-bipyridin-5-yl)alanine·3HCl, 2

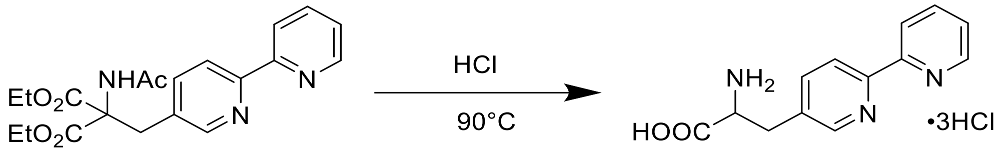

Compound **S4** (11.6 g, 30 mmol) was heated to 90 °C in HCl (100 mL, 12 N aq.) for 16 h. The solvent was removed *in vacuo* giving BpyAla **2** as a white solid (10.4 g obtained, qt. yield). Analytical data in accordance with literature [6]. **^{1}H-NMR** δ (500 MHz, D_2O) 8.73 (1H, d, J = 5.6 Hz) 8.68 (1H, s), 8.52 (1H, t, J = 7.9 Hz), 8.46 (1H, d, J = 8.1 Hz), 8.23 (1H, d, J = 8.2 Hz), 8.08 (1H, dd, J = 1.6 Hz), 7.93 (1H, t, J = 6.6 Hz), 4.40 (1H, t, J = 6.8 Hz), 3.38 (2H, dd, J = 14.8, 6.8 Hz). **^{13}C NMR** (126 MHz, Methanol-d_4) δ 170.53, 150.48, 149.03, 147.38, 146.81, 145.30, 142.98, 136.63, 128.43, 125.26, 124.25, 54.25, 34.20. **HRMS** $C_{13}H_{13}N_3O_2$ $[M+H]^+$ m/z calculated for $C_{13}H_{14}N_3O_2$ 244.1086, found 244.0860

Literature

[1] A. G. Jarvis *et al.*, "Enzyme Activity by Design: An Artificial Rhodium Hydroformylase for Linear Aldehydes," *Angew. Chemie*, vol. 129, no. 44, pp. 13784–13788, Oct. 2017.

[2] H. Liu and J. H. Naismith, "An efficient one-step site-directed deletion, insertion, single and multiple-site plasmid mutagenesis protocol," *BMC Biotechnol.*, vol. 8, no. 1, p. 91, Dec. 2008.

[3] I. Drienovská, A. Rioz-Martínez, A. Draksharapu, and G. Roelfes, "Novel artificial metalloenzymes by in vivo incorporation of metal-binding unnatural amino acids," *Chem. Sci.*, vol. 6, no. 1, pp. 770–776, Dec. 2015.

[4] T. Koshiyama *et al.*, "pH-Dependent ion permeability control of a modified amphotericin B channel through metal complexation," *Chem. Commun.*, vol. 57, no. 23, pp. 2895–2898, Mar. 2021.

[5] S. L. Hyun and P. G. Schultz, "Biosynthesis of a site-specific DNA cleaving protein," *J. Am. Chem. Soc.*, vol. 130, no. 40, pp. 13194–13195, Oct. 2008.

[6] B. Imperiali, T. J. Prins, and S. L. Fisher, "Chemoenzymatic Synthesis of 2-Amino-3-(2,2′-bipyridinyl)propanoic Acids," *J. Org. Chem.*, vol. 58, no. 6, pp. 1613–1616, 1993.
