# Supplementary material for: Using BpyAla to generate copper artificial metalloenzymes: a catalytic and structural study
Source: Catal Sci Technol. 2024 Jan 29;14(6):1622–32. doi: 10.1039/d3cy01648j (PMC10946309; doi:10.1039/d3cy01648j)
Supplement: CY-014-D3CY01648J-s002 [file CY-014-D3CY01648J-s002.pdf]

Electronic Supplementary Material (ESI) for Catalysis Science & Technology.
This journal is © The Royal Society of Chemistry 2024

Supporting Information B

Using BpyAla to generate Copper Artificial Metalloenzymes: a catalytic and structural study

E. Klemencic[1], R. C. Brewster[1], H. S. Ali[1,2], J. M. Richardson[3], A. G. Jarvis[1]*
[1]School of Chemistry, Joseph Black Building, Kings Buildings, University of Edinburgh, David Brewster Road, Edinburgh, EH9 3FJ. [2] Current address: Chemistry Research Laboratory, Department of Chemistry and the INEOS Oxford Institute for Antimicrobial Research, University of Oxford, 12 Mansfield Road, Oxford OX1 3TA, UK. [3] School of Biological Sciences, Swann Building, University of Edinburgh, Edinburgh, EH9 3BF.
*Corresponding author: amanda.jarvis@ed.ac.uk

1. Computational Studies.

1.1 System Set-up

The X-ray structures of Cu(II)-bound SCP-Q111CBpy and SCP-Q111BpyAla were considered as host proteins. Missing hydrogen atoms were added using Chimera[1] at pH 7.4 while the crystallographic water molecules bound to Cu(II) were manually removed. The molecular structures of compound **3** (5-methoxy-1H-indole) and compound **4** (1-(1-methyl-1H-imidazol-2-yl)but-2-en-1-one) were considered as substrates and saved in the PDB file using Avogadro.[2] The molecular docking of the substrates to each protein was carried out using AutoDock Vina and evaluated according to the lowest binding scores. The mutation study of different amino acids was constructed using the Dunbrack backbone-dependent rotamer library[4,5] within Chimera.[1]

1.2 Molecular Dynamics Simulation.

To prepare the system for initial Molecular Dynamics (MD) simulations the X-ray crystal structures of SCP_Q111CBpy and SCP_Q111BpyAla with Cu(II) ion and Triton-X-100 were considered (apo state) (PDB IDs 8AF3 and 8AF2 respectively). In another set of MD simulations the lowest Vina docking score poses of the substrate **3** and substrate **4** bound to SCP_Q111CBpy and SCP_Q111BpyAla were used. MD simulations were prepared using xleap available in AMBER Tools 18.[6] The force field parameters for the unnatural amino acids cystine bipyridine and bipyridine alanine were generated using RESP charge fitting[7] in Antechamber[8], while for other amino acids the ff14SB[9] force field parameters were used. The parameters for square planar copper ion (Cu (II)) either with water, as in case of apo state, or with substrate, were generated using AMBER's Metal Center Parameter Builder (MCPB).[10] To generate charges, the ChgModB method was used at the UB3LYP/6-311G* level of theory, using the MCPB.py v3.0 script.[10] Further force field parameters were generated using the RESP[7] and Seminario method.[11] Furthermore, for both substrates the second generation General Amber Force Field (GAFF2)[12] with AM1-BCC charges, generated using the Antechamber[8] and Parmchk2 within AMBER 18 were used. Finally, the protein and substrate complexes were solvated in a cubic box of TIP3P[13] water molecules which were further neutralized by adding Na+ ions, whose parameters were taken from the Joung and Cheatham.[14]

All MD simulation were carried out by the Particle Mesh Ewald Molecular Dynamics (PMEMD) module of AMBER 18. The systems were initially minimized using 2000 steps of steepest decent minimization with 2.0 kcal mol^{-1} as a restraints potential on heavy atoms of protein. After that these were heated for 100 ps from 0 to 298.15 K temperature using the NVT ensemble and a Langevin thermostate[15] with collision frequency of 5 ps^{-1}, while the equilibration of the system was done at 1 ns using the NPT simulation with pressure coupling using the Berendsen barostate[16] while gradually removing the restraining. Furthermore, 200 ns NPT MD simulations have been carried out using 2 fs time step, SHAKE protocol on hydrogen atoms and 10 Å non-bonded cut-off with periodic boundary conditions. In total four 200 ns MD simulations as a triplicate were performed: the SCP_Q111CBpy and SCP_Q111BpyAla apo enzyme, the substrate bound complex of SCP_Q111CBpy and the substrate bound complex of SCP_Q111BpyAla. The data was stored every 1 ps and visualized with CPPTRAJ and PYTRAJ in AMBER[17], Chimera[1] and VMD.[18]

1.3 DFT Cluster Models.

Cluster models focused on the substrate binding environment including the second coordination sphere and considered all atoms in the quantum mechanical approach.[19-20] Three cluster models (**A, B** and **C)** were created based on the final equilibrated structure from 200 ns MD simulations as shown in Figure S16. We truncated the active site models by considering the amino acid residues which are in close proximity of the substrate and determine the substrate binding and positioning, particularly all polar hydrogen containing amino acids which are close to metal and substrate. Model **A** (the minimal cluster model) consisted of 2,2-bipyridine attached to Cu(II) and substrates (**3** and **4**), and had 58 atoms in total. Thereafter, large cluster models **B** and **C** were created that contains the environment around the copper and substrates, and all the hydrogen binding around them. Thus, the model **B** consist of one short chains amino acids, L_{87}-D_{88} around the substrates and a polar amino acid K_{91} and some other amino acids including G_{85}, Q_{107} and M_{112} attached to $BpyAla_{111}$. Furthermore, five water molecules and both substrates **3** and **4** were included in the active site model **B**. While the model **C** which were created from the MD equilibrated structural coordinates of SCP_Q111CBpy. Thus, the. Model **C** consist of amino acids including the Q_{90}, S_{107}, Q_{108} and two short chains of amino acids F_{93}-F_{94} and I_{104}-M_{105} which surrounds the substrates. Furthermore, an unnatural amino acid $CBpy_{111}$ and both substrates (**3** and **4**) were the part of this active site model. Overall, the model **B** and **C** consists of 180 and 183 atoms respectively and a charge of +2. The extract of both these models (**A, B** and **C**) are shown in Figure S1.

All the quantum mechanical calculations discussed in this work were carried out using Gaussian-09 software package.[21] Following our previous experience with cluster models of reactions catalyzed by metalloenzymes,[22,23] we applied unrestricted B3LYP hybrid functional of density functional theory (DFT) in combination with 6-31G* on all atoms except cupper atom and for cupper LANL2DZ (with effective core potential) basis sets:BS1.[24-26] To find out the local minima the geometry optimizations and the frequency calculations at 298.15 K temperature and 1 atm pressure were carried out using BS1 level of DFT method, and were confirmed by visualizing the all positive eigenvalues in frequency calculations. While all the transitions state structures were searched with Berny algorithm,[27] and were confirmed by visualizing and animating the single negative eigenvalues of frequency calculations. In order to correct the energetics and consider solvent effect, single point energy calculations were performed using the higher level of basis set: BS2: UB3LYP-D3/6-311+G*/LACV3P+ (with effective core potential) on cupper. Furthermore, these single point energy calculations also have continuum polarized conductor implicit solvent model (CPCM) with dielectric constant ($\varepsilon = 5.7$) mimicking chlorobenzene[27] and Grimme dispersion correction (D3).[28] All the energy values and structural coordinates are in the electronic supplementary information.

2. Results and discussions

To gain further insight into the novel ArMs, triplicates of 200 ns molecular dynamics (MD) simulation for the SCP_Q111CBpy and SCP_Q111BpyAla were carried out. The structural effects of these ArMs were probed using root means square deviation (RMSD), root mean square fluctuation (RMSF) and radius of gyration. RMSD is frequently used to confirm the stability and convergence of the computer-generated models while the RMSF is commonly used to study the thermal fluctuations of amino acids/atoms during MD simulation of enzyme structures. The RMSD profiles for all the alpha carbon (C_α) atoms for SCP_Q111CBpy and SCP_Q111BpyAla in their apo forms (without substrates) are shown in Figure S2 and Figure S3, respectively. To study whether the structures of both these ArMs converged and remained stable during the course of MD simulation, we analyzed the RMSD and RMSF in the apo state and with substrates bound (Figures S2-S7). The apo and substrate-bound forms of both enzymes converged well and remained stable during 200 ns MD simulations with minimal overall deviations (Figure S2-S5). However, to some extent the SCP_Q111CBpy structure is more flexible than SCP_Q111BpyAla in the substrate-bound form. Figures S6 and S7 indicate

that the thermal fluctuations of amino acids in both enzymes in both apo and substrate-bound form are similar. However, in the substrate-bound form of SCP_Q111CBpy residues-wise fluctuations are slightly higher, particularly in a small loop of amino acids Val_{79}-Leu_{80}-Gly_{81}-Lys_{82} in the center of tunnel. The radius of gyration (RG), which relates to the compactness of the SCP_Q111CBpy and SCP_Q111BpyAla structures, was analysed during the course of MD simulations (Figures S8 and S9, respectively). The results revealed that both crystal structures were stable and remained compact during the simulation. The average RG for the SCP_Q111CBpy structure (13.9 Å) is slightly higher than that of SCP_Q111BpyAla (13.5 Å), indicating that SCP_Q111CBpy structure is less compact. Overall, all MD simulations parameters (RMSD, RMSF and RG) show that the cysteine-linked bipyridine ArM structure (SCP_Q111CBpy) is more flexible than the alanine-linked bipyridine (SCP_Q111BpyAla).

3. Catalysis

We explored the Friedel-Craft alkylation reaction of 1*H*-indole **3** with 1-(1-methyl-1*H*-imidazol-2-yl)but-2-en-1-one **4** to give the 3-(1*H*-indol-3-yl)-1-(1-methyl-1*H*-imidazol-2-yl)butan-1-one product **5** as a benchmark reaction catalyzed by Cu(II). We started computational modeling to gain insight into the enantioselective nature of Friedel-Craft (FC) alkylation using the density functional theory (DFT) method. We created three models (**A**, **B** and **C**) for DFT calculations. In model **A** the reaction is catalyzed by Cu(II)-2,2'-bipyridine, and can be divided into two steps typically found in FC alkylation[30,31] (Scheme S1). The first step is the conjugate addition of indole **3** to enone **4** to form intermediate (**Int1**) via transition state (**TS1**) and the second step is the product formation by the protonation reaction via second transition state (**TS2**). The first step is an enantioselective step which leads to two isomer intermediates: $\mathbf{Int1_{proS,A}}$ and $\mathbf{Int1_{proR,A}}$ via the $\mathbf{TS1_{proS,A}}$ and $\mathbf{TS1_{proR,A}}$ transition states. The reaction energy barrier to obtain $\mathbf{Int1_{proS,A}}$ is 7.3 kcal mol^{-1}, while for $\mathbf{Int1_{proR,A}}$ it is 7.6 kcalmol^{-1}. The second step is the transfer of a proton from the conjugated carbon of enone **4** to carbonyl oxygen of **4** leading to the enol product via the $\mathbf{TS2_{enol}}$ or beta carbon of **4** via the $\mathbf{TS2_{keto}}$, leading to the keto product. The formation of enol products from both intermediate enantiomers is highly unlikely because of the very high energy barriers of 28.5 kcal mol^{-1} and 29.0 kcal mol^{-1} for $\mathbf{TS2_{enol,proS,A}}$ and $\mathbf{TS2_{enol,proR,A}}$ respectively. By contrast, there is a lower energy barrier for the formation of the keto products $\mathbf{Prod_{keto,ProS,A}}$ and $\mathbf{Prod_{keto,ProR,A}}$**:** 5.9 kcal mol^{-1} and 6.5 kcal mol^{-1}, respectively. The complete reaction free-energy profile calculated with unrestricted B3LYP-D3/BS2 level of DFT method is presented in Figure S11 and the

optimized geometric structures of transition states and intermediates with the key distances are presented in Figure S12-S15. Overall, our computational analysis shows that the conjugate coupling step is the rate determining step as well as the enantioselective step and the formation of keto product is most likely. This agrees well with our experimental results as well as previous studies[31] so we decided to focus on the first step only for the rest of our work. To enhance the enantioselectivity we catalyzed the FC reaction with newly synthesized artificial metalloenzymes and presented in Table 1.

4. References:

1. Pettersen, E. F.; Goddard, T. D.; Huang, C. C.; Couch, G. S.; Greenblatt, D. M.; Meng, E. C.; Ferrin, T. E., UCSF Chimera—A visualization system for exploratory research and analysis. *Journal of Computational Chemistry* **2004,** *25*, 1605-1612.
2. Hanwell, M. D.; Curtis, D. E.; Lonie, D. C.; Vandermeersch, T.; Zurek, E.; Hutchison, G. R., Avogadro: an advanced semantic chemical editor, visualization, and analysis platform. *Journal of Cheminformatics* **2012,** *4*, 17.
3. Trott, O.; Olson, A. J., AutoDock Vina: improving the speed and accuracy of docking with a new scoring function, efficient optimization, and multithreading. *Journal of computational chemistry* **2010,** *31*, 455-461.
4. Dunbrack, R. L.; Karplus, M., Backbone-dependent Rotamer Library for Proteins Application to Side-chain Prediction. *Journal of Molecular Biology* **1993,** *230*, 543-574.
5. Dunbrack, R. L., Rotamer Libraries in the 21st Century. *Current Opinion in Structural Biology* **2002,** *12*, 431-440.
6. D.A. Case, I.Y. Ben-Shalom, S.R. Brozell, D.S. Cerutti, T.E. Cheatham, III, V.W.D. Cruzeiro, T.A. Darden, R.E. Duke, D. Ghoreishi, M.K. Gilson, H. Gohlke, A.W. Goetz, D. Greene, R Harris, N. Homeyer, Y. Huang, S. Izadi, A. Kovalenko, T. Kurtzman, T.S. Lee, S. LeGrand, P. Li, C. Lin, J. Liu, T. Luchko, R. Luo, D.J. Mermelstein, K.M. Merz, Y. Miao, G. Monard, C. Nguyen, H. Nguyen, I. Omelyan, A. Onufriev, F. Pan, R. Qi, D.R. Roe, A. Roitberg, C. Sagui, S. Schott-Verdugo, J. Shen, C.L. Simmerling, J. Smith, R. Salomon- Ferrer, J. Swails, R.C. Walker, J. Wang, H. Wei, R.M. Wolf, X. Wu, L. Xiao, D.M. York and P.A. Kollman (2018), AMBER 2018, University of California, San Francisco.
7. Bayly, C. I.; Cieplak, P.; Cornell, W.; Kollman, P. A., A well-behaved electrostatic potential based method using charge restraints for deriving atomic charges: the RESP model. *The Journal of Physical Chemistry* **1993,** *97*, 10269-10280.
8. Wang, J.; Wang, W.; Kollman, P. A.; Case, D. A., Automatic atom type and bond type perception in molecular mechanical calculations. *Journal of Molecular Graphics and Modelling* **2006,** *25*, 247-260.
9. Maier, J. A.; Martinez, C.; Kasavajhala, K.; Wickstrom, L.; Hauser, K. E.; Simmerling, C., ff14SB: Improving the Accuracy of Protein Side Chain and

Backbone Parameters from ff99SB. *Journal of Chemical Theory and Computation* **2015,** *11*, 3696-3713.
10. Li, P.; Merz, K. M., MCPB.py: A Python Based Metal Center Parameter Builder. *Journal of Chemical Information and Modeling* **2016,** *56*, 599-604.
11. Seminario, J. M., Calculation of intramolecular force fields from second-derivative tensors. *International Journal of Quantum Chemistry* **1996,** *60*, 1271-1277.
12. Träg, J.; Zahn, D., Improved GAFF2 parameters for fluorinated alkanes and mixed hydro- and fluorocarbons. *Journal of Molecular Modeling* **2019,** *25*, 39.
13. Jorgensen, W. L.; Chandrasekhar, J.; Madura, J. D.; Impey, R. W.; Klein, M. L., Comparison of simple potential functions for simulating liquid water. *The Journal of Chemical Physics* **1983,** *79*, 926-935.
14. Joung, I. S.; Cheatham, T. E., Determination of Alkali and Halide Monovalent Ion Parameters for Use in Explicitly Solvated Biomolecular Simulations. *The Journal of Physical Chemistry B* **2008,** *112*, 9020-9041.
15. Loncharich, R. J.; Brooks, B. R.; Pastor, R. W., Langevin dynamics of peptides: the frictional dependence of isomerization rates of N-acetylalanyl-N'-methylamide. *Biopolymers* **1992,** *32*, 523-35.
16. Lin, Y.; Pan, D.; Li, J.; Zhang, L.; Shao, X., Application of Berendsen barostat in dissipative particle dynamics for nonequilibrium dynamic simulation. *The Journal of Chemical Physics* **2017,** *146*, 124108.
17. Roe, D. R.; Cheatham, T. E., PTRAJ and CPPTRAJ: Software for Processing and Analysis of Molecular Dynamics Trajectory Data. *Journal of Chemical Theory and Computation* **2013,** *9*, 3084-3095.
18. Humphrey, W.; Dalke, A.; Schulten, K., VMD: Visual molecular dynamics. *Journal of Molecular Graphics* **1996,** *14*, 33-38.
19. Blomberg, M. R. A.; Borowski, T.; Himo, F.; Liao, R.-Z.; Siegbahn, P. E. M., Quantum Chemical Studies of Mechanisms for Metalloenzymes. *Chemical Reviews* **2014,** *114*, 3601-3658.
20. de Visser, S. P., Mechanistic Insight on the Activity and Substrate Selectivity of Nonheme Iron Dioxygenases. *Chem Rec* **2018,** *18*, 1501-1516.
21. *Gaussian*-09, Revision D.01, Frisch, M. J.; Trucks, G. W.; Schlegel, H. B.; Scuseria, G. E.; Robb, M. A.; Cheeseman, J. R.; Scalmani, G.; Barone, V.; Mennucci, B.; Petersson, G. A.; Nakatsuji, H.; Caricato, M.; Li, X.; Hratchian, H. P.; Izmaylov, A. F.; Bloino, J.; Zheng, G.; Sonnenberg, J. L.; Hada, M.; Ehara, M.; Toyota, K.; Fukuda, R.; Hasegawa, J.; Ishida, M.; Nakajima, T.; Honda, Y.; Kitao, O.; Nakai, H.; Vreven, T.; Montgomery Jr, J. A.; Peralta, J. E.; Ogliaro, F.; Bearpark, M.; Heyd, J. J.; Brothers, E.; Kudin, K. N.; Staroverov, V. N.; Keith, T.; Kobayashi, R.; Normand, J.; Raghavachari, K.; Rendell, A.; Burant, J. C.; Iyengar, S. S.; Tomasi, J.; Cossi, M.; Rega, N.;. Millam, J. M.; Klene, M.; Knox, J. E.; Cross, J. B.; Bakken, V.; Adamo, C.; Jaramillo, J.; Gomperts, R.; Stratmann, R. E.; Yazyev, O.; Austin, A. J.; Cammi, R.; Pomelli, C.; Ochterski, J. W.; Martin, R. L.; Morokuma, K.; Zakrzewski, V. G.; Voth, G. A.; Salvador, P.; Dannenberg, J. J.; Dapprich, S.; Daniels, A. D.; Farkas, O.; Foresman, J. B.; Ortiz, J. V.; Cioslowski, J.; Fox, D. J. Gaussian, Inc., Wallingford CT, 2013.

22. Louka, S.; Barry, S. M.; Heyes, D. J.; Mubarak, M. Q. E.; Ali, H. S.; Alkhalaf, L. M.; Munro, A. W.; Scrutton, N. S.; Challis, G. L.; de Visser, S. P., Catalytic Mechanism of Aromatic Nitration by Cytochrome P450 TxtE: Involvement of a Ferric-Peroxynitrite Intermediate. *Journal of the American Chemical Society* **2020,** *142*, 15764-15779.
23. Ali, H. S.; Henchman, R. H.; de Visser, S. P., Lignin Biodegradation by a Cytochrome P450 Enzyme: A Computational Study into Syringol Activation by GcoA. *Chemistry – A European Journal* **2020,** *26*, 13093-13102.
24. Becke, A. D., Density-functional thermochemistry. III. The role of exact exchange. *The Journal of Chemical Physics* **1993,** *98*, 5648-5652.
25. Hay, P. J.; Wadt, W. R., Ab initio effective core potentials for molecular calculations. Potentials for the transition metal atoms Sc to Hg. *The Journal of Chemical Physics* **1985,** *82*, 270-283.
26. Hehre, W. J.; Ditchfield, R.; Pople, J. A., Self—Consistent Molecular Orbital Methods. XII. Further Extensions of Gaussian—Type Basis Sets for Use in Molecular Orbital Studies of Organic Molecules. *The Journal of Chemical Physics* **1972,** *56*, 2257-2261.
27. Peng, C.; Bernhard Schlegel, H., Combining Synchronous Transit and Quasi-Newton Methods to Find Transition States. *Israel Journal of Chemistry* **1993,** *33*, 449-454.
28. Cossi, M.; Scalmani, G.; Rega, N.; Barone, V., New developments in the polarizable continuum model for quantum mechanical and classical calculations on molecules in solution. *The Journal of Chemical Physics* **2002,** *117*, 43-54.
29. Grimme, S.; Antony, J.; Ehrlich, S.; Krieg, H., A consistent and accurate ab initio parametrization of density functional dispersion correction (DFT-D) for the 94 elements H-Pu. *The Journal of Chemical Physics* **2010,** *132*, 154104.
30. Wu, H.; Zhao, T.; Hu, X., Friedel-Crafts Reaction of N,N-Dimethylaniline with Alkenes Catalyzed by Cyclic Diaminocarbene-Gold(I) Complex. *Scientific Reports* **2018,** *8* (1), 11449.
31. Villarino, L.; Chordia, S.; Alonso-Cotchico, L.; Reddem, E.; Zhou, Z.; Thunnissen, A. M. W. H.; Maréchal, J.-D.; Roelfes, G., Cofactor Binding Dynamics Influence the Catalytic Activity and Selectivity of an Artificial Metalloenzyme. *ACS Catalysis* **2020,** *10* (20), 11783-11790.

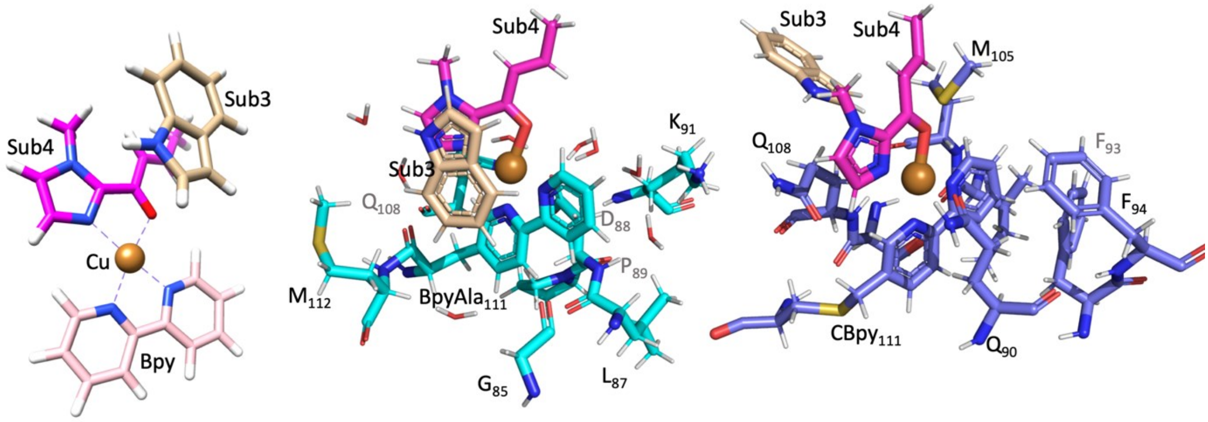

Figure S1. The active-site models bound to substrate 3 and substrate 4. The large cluster models had 180 (model B) and 183 (model C) atoms in total, including Cu(II), substrates (3 and 4) and the first and second coordination sphere amino acids, representing the protein environment.

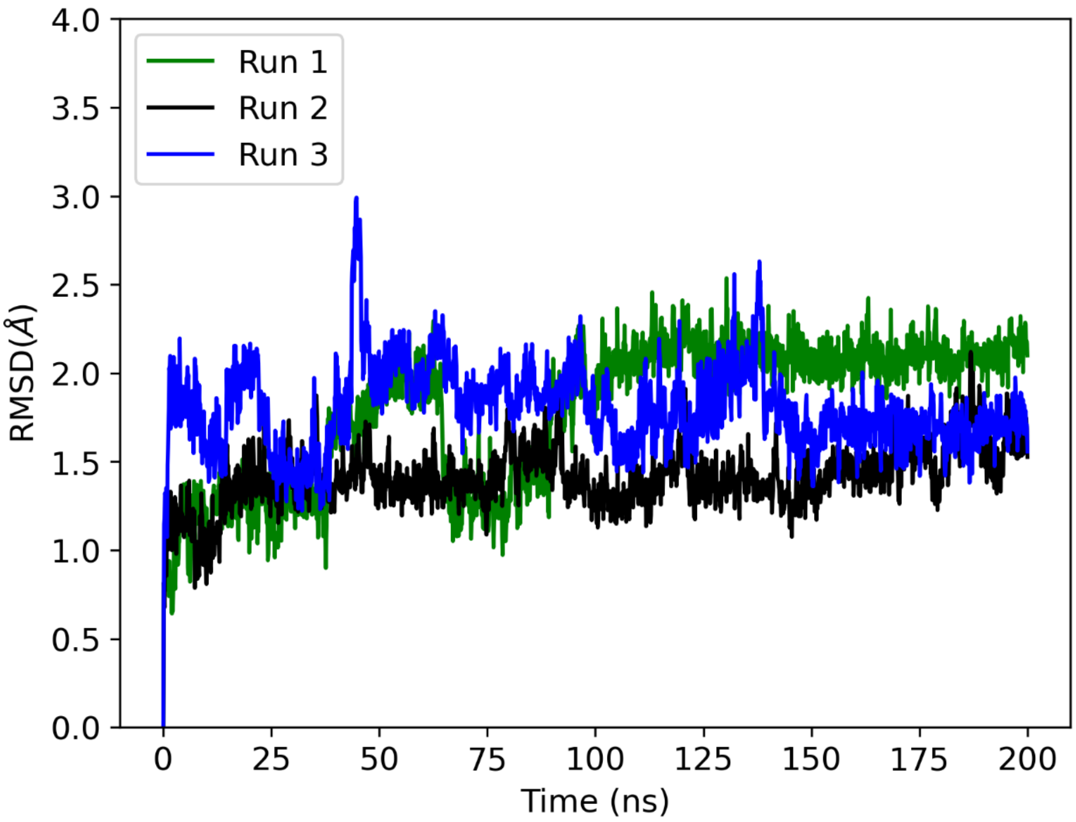

Figure S2: RMSD of C-alpha atoms for three individual MD trajectories and for an average trajectory for the 200 ns simulation for SCP-Q111CBpy.

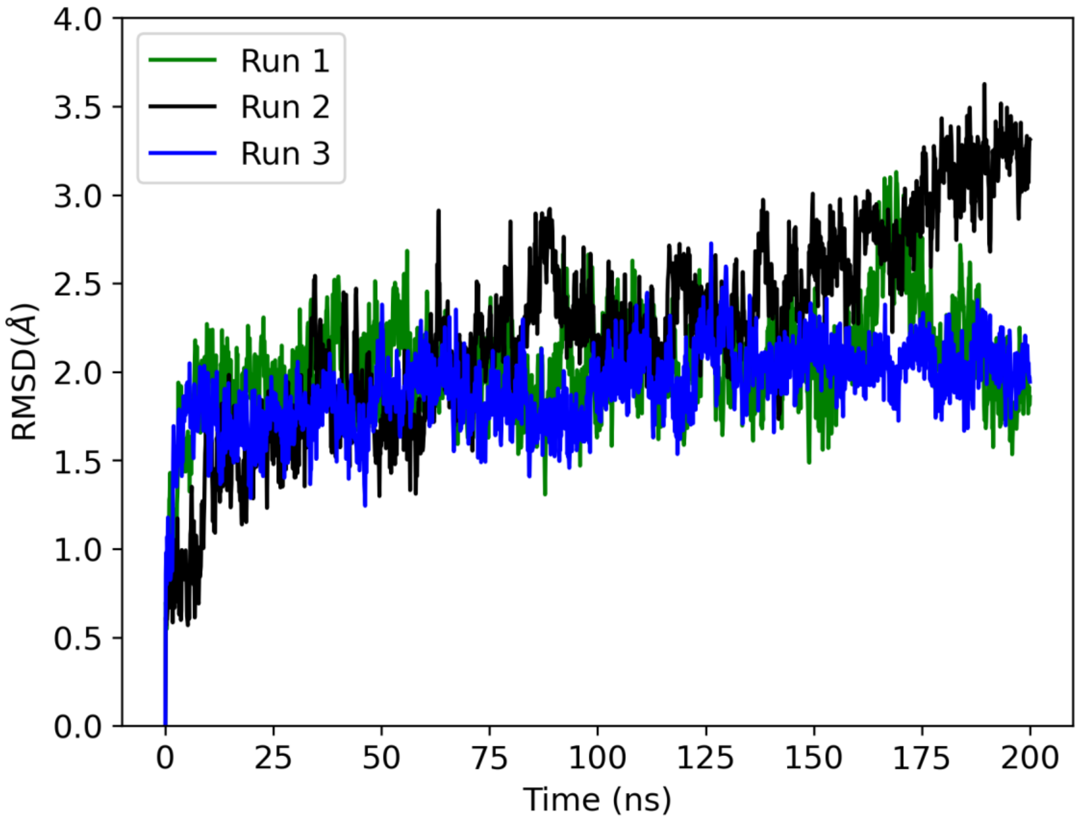

MD

Figure S3: RMSD of C-alpha atoms for four individual MD trajectories and for an average trajectory for the 200 ns MD simulation for SCP-Q111Bpyala.

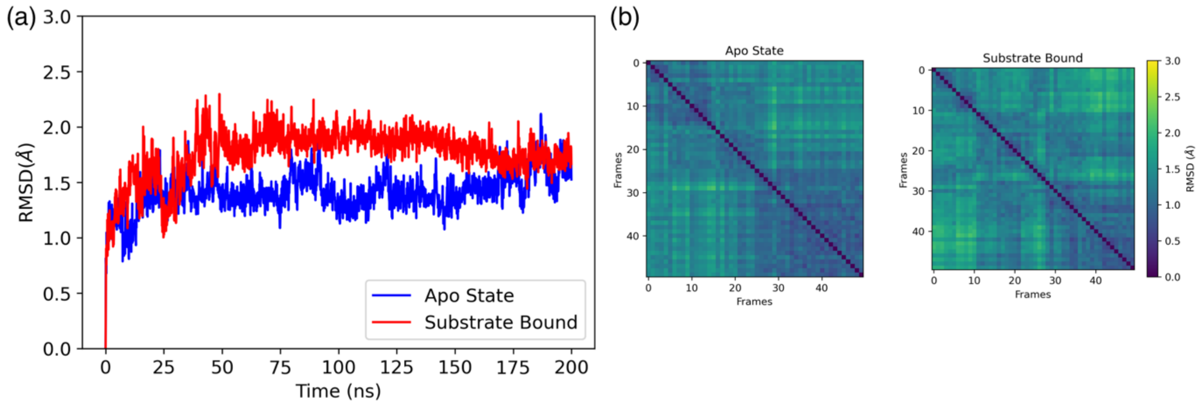

Figure S4: Convergence analysis used in this study to assess the stability/flexibility of the SCP-Q111CBpy for 200 ns of the MD trajectories: (a) Backbone RMSD (b) all frame to frame RMSD.

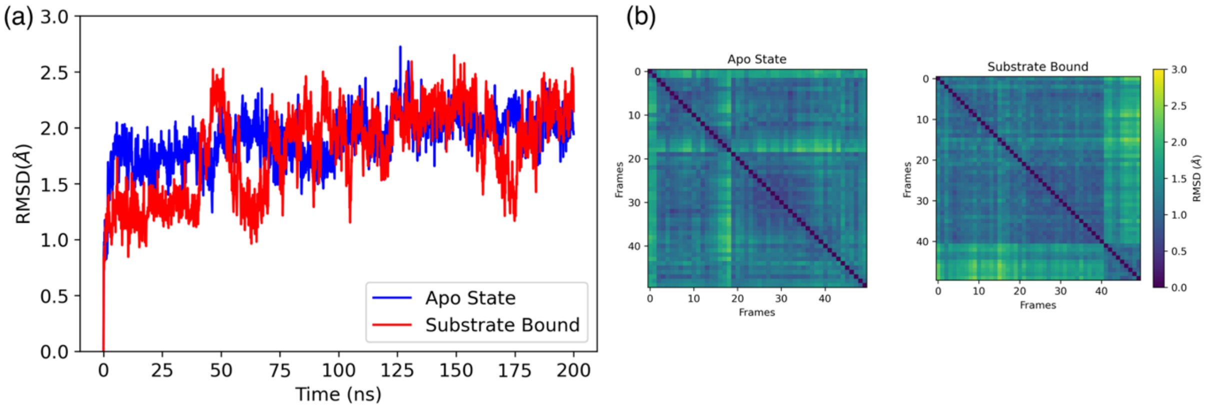

Figure S5: Convergence analysis used in this study to assess the stability/flexibility of the SCP-Q111BpyAla for 200 ns of the MD trajectories: (a) Backbone RMSD (b) all frame to frame RMSD.

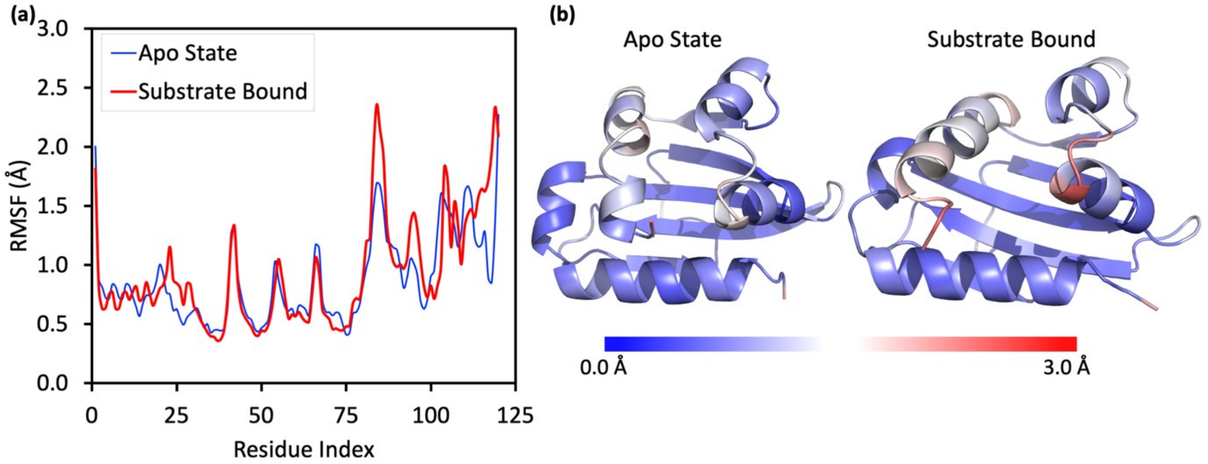

Figure S6: Convergence analysis used in this study to assess the stability/flexibility of the SCP-Q111CBpy for 200 ns of the MD trajectories: (a) RMSF and (b) RMSF for Apo State (left) and Substrate Bound (right) structures.

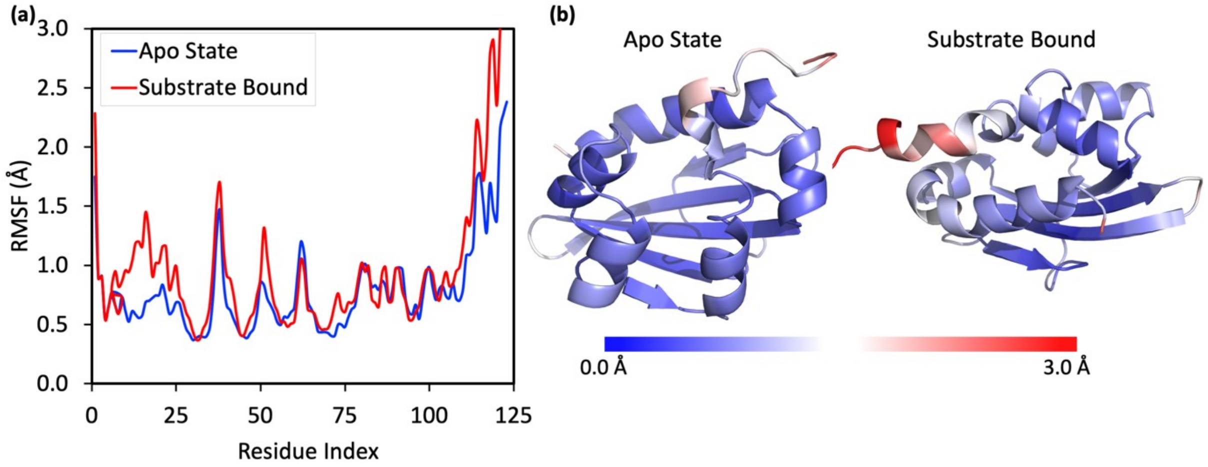

Figure S7: Convergence analysis used in this study to assess the stability/flexibility of the SCP-Q111BpyAla for 200 ns of the MD trajectories: (a) RMSF and (b) RMSF for Apo State (left) and Substrate Bound (right) structures.

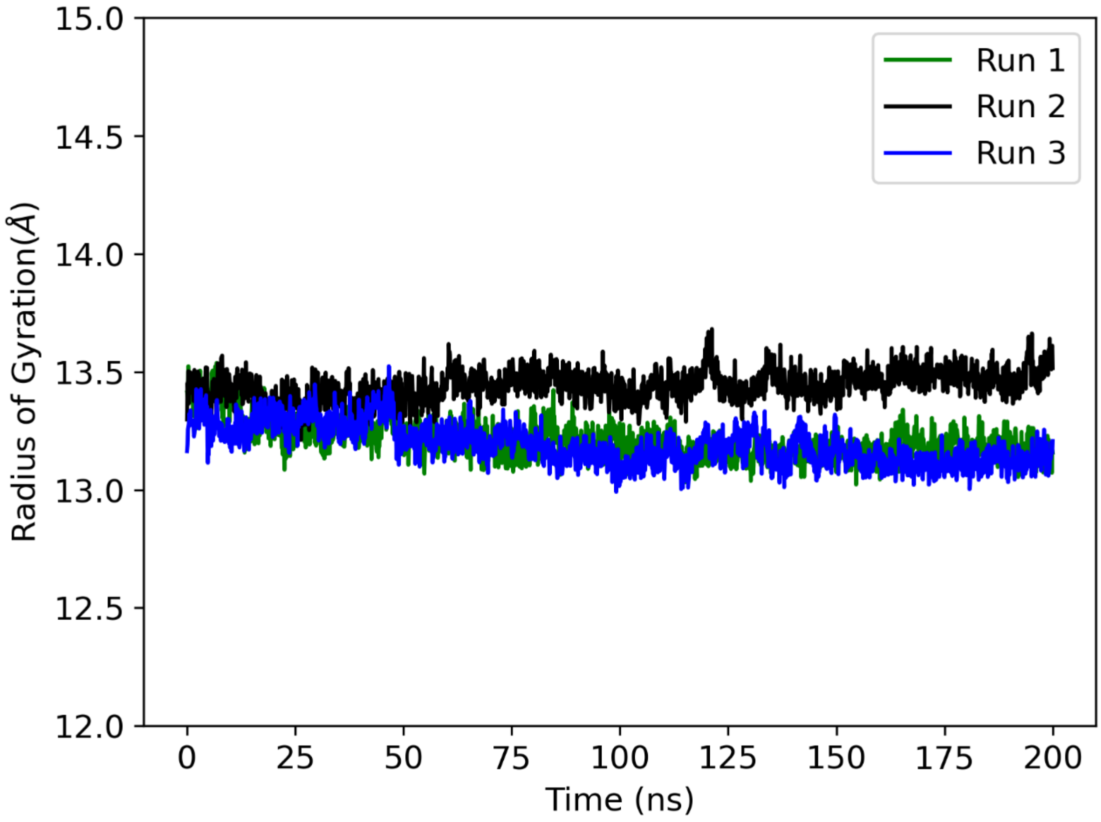

Figure S8: Radius of gyration for four individual MD trajectories and for an average trajectory for the 200 ns MD for SCP-Q111CBpy.

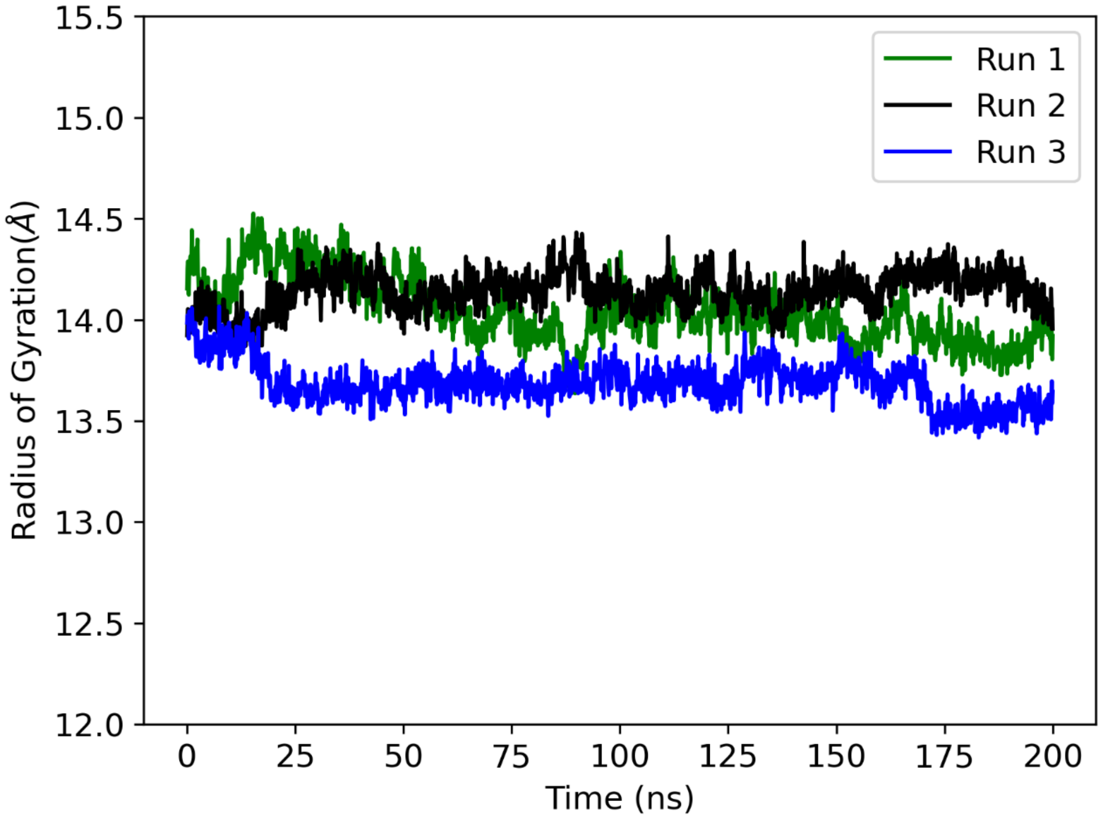

Figure S9: Radius of gyration for four individual MD trajectories and for an average trajectory for the 200 ns MD for SCP-Q111BpyAla.

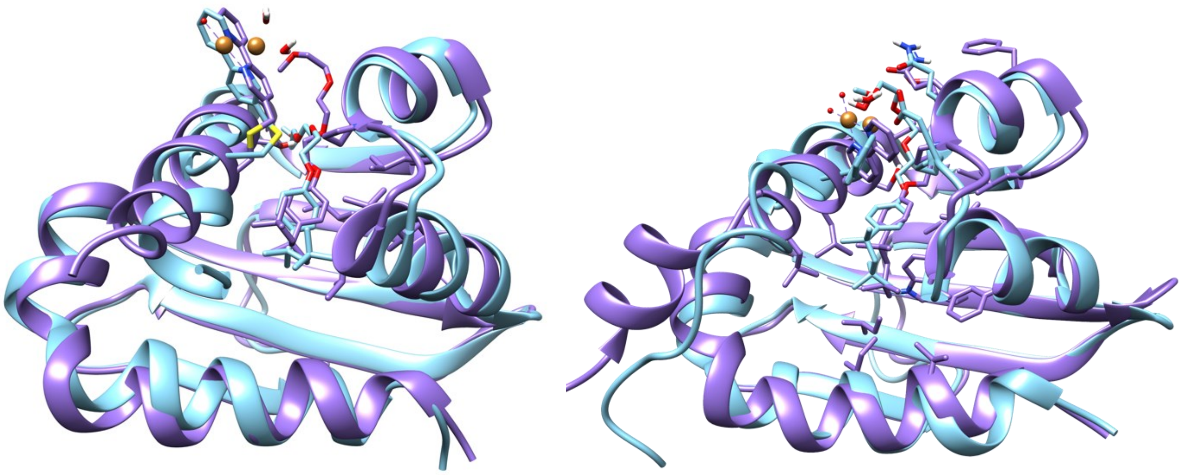

Figure S10: Overlay of X-ray crystal (in purple) and 200 ns MD equilibrated (in light blue) for SCP-Q111CBpy (left) and SCP-Q111BpyAla (right).

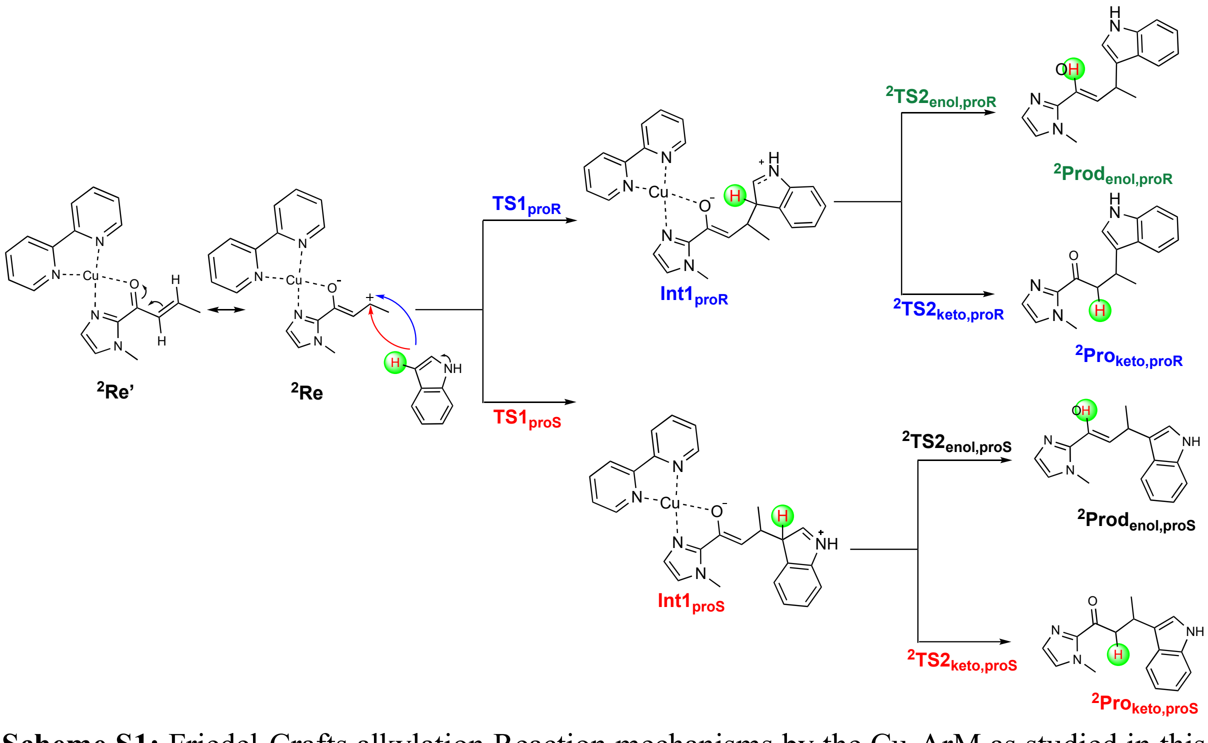

Scheme S1: Friedel-Crafts alkylation Reaction mechanisms by the Cu-ArM as studied in this work.

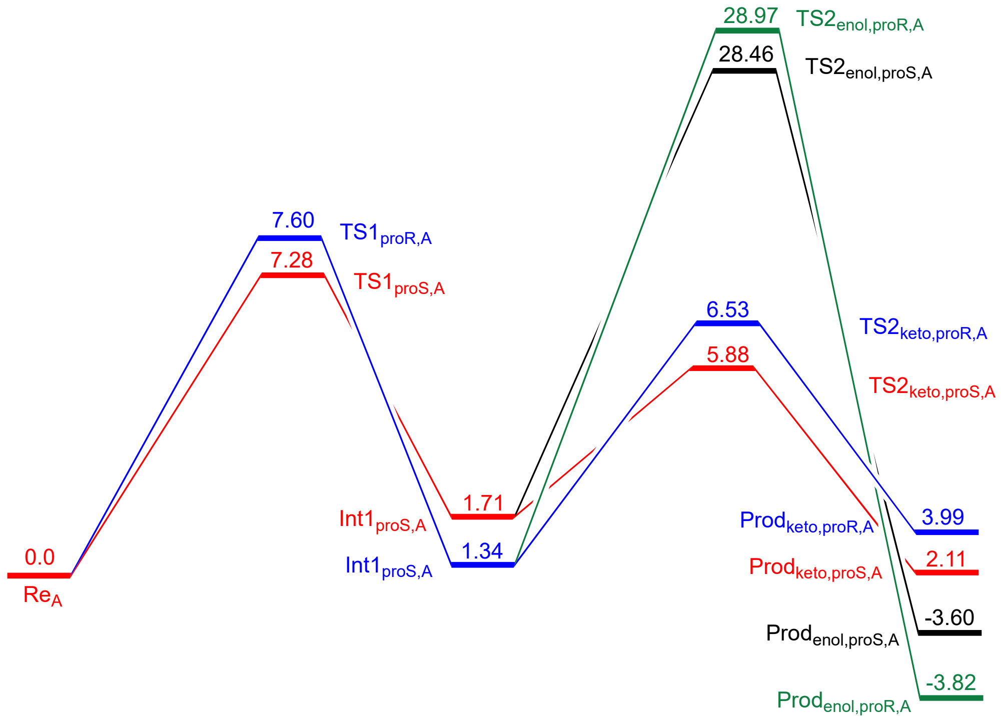

Figure S11: Potential energy landscape for the Friedel-Crafts alkylation in Gas phase at UB3LYP-D3/6-31G* level of Density Functional Theory (DFT). Energy values are in kcal/mol.

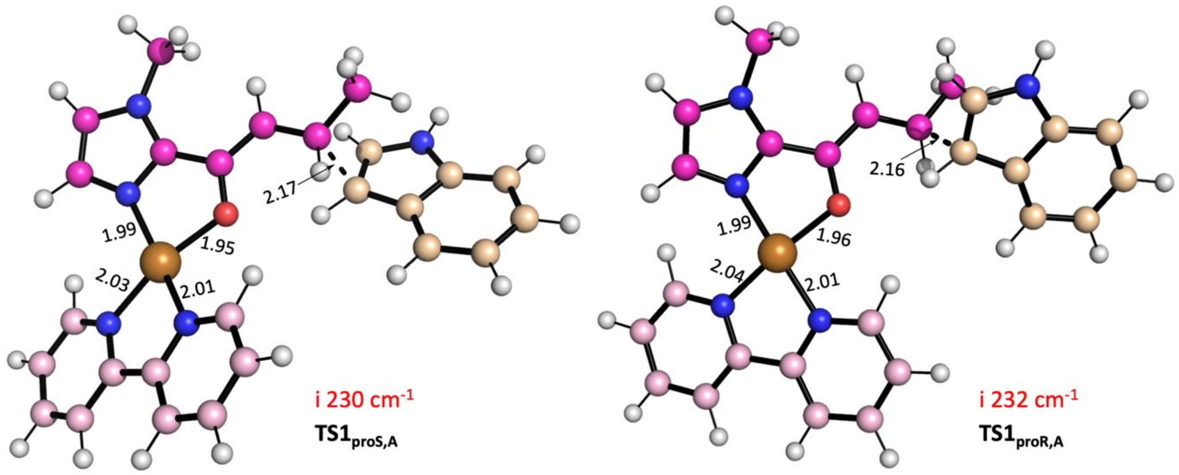

Figure S12: UB3LYP/BS1 optimized geometries of $TS1_{proS,A}$ (left side) and $TS1_{proR,A}$ (right side) structures as obtained in Gaussian 09. Bond lengths are in angstroms and the imaginary frequencies are in cm^{-1}.

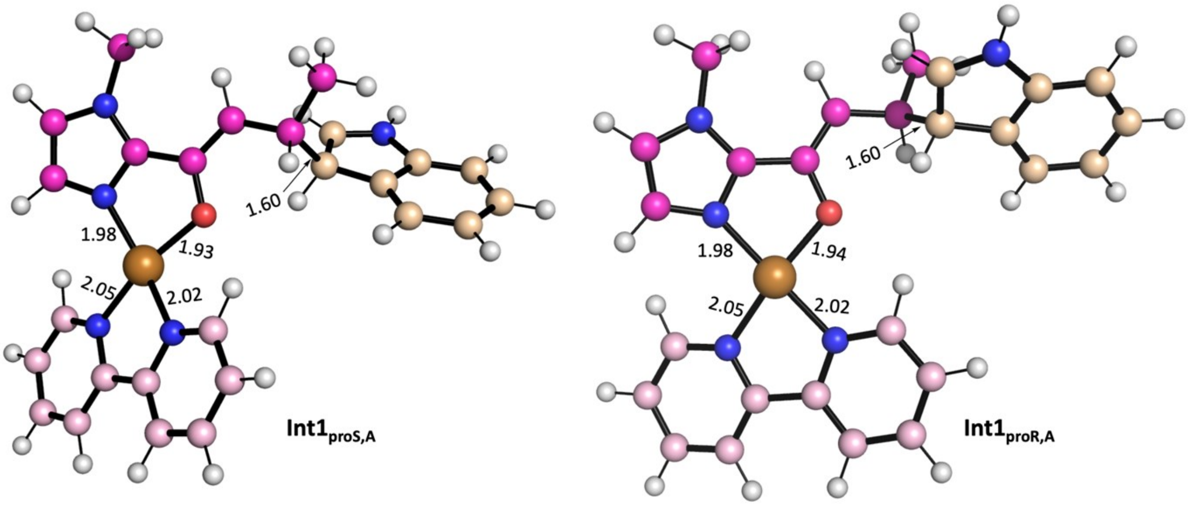

Figure S13: UB3LYP/BS1 optimized geometries of Int1$_{proS,A}$ (left side) and Int1$_{proR,A}$ (right side) structures as obtained in Gaussian 09. Bond lengths are in Angstroms.

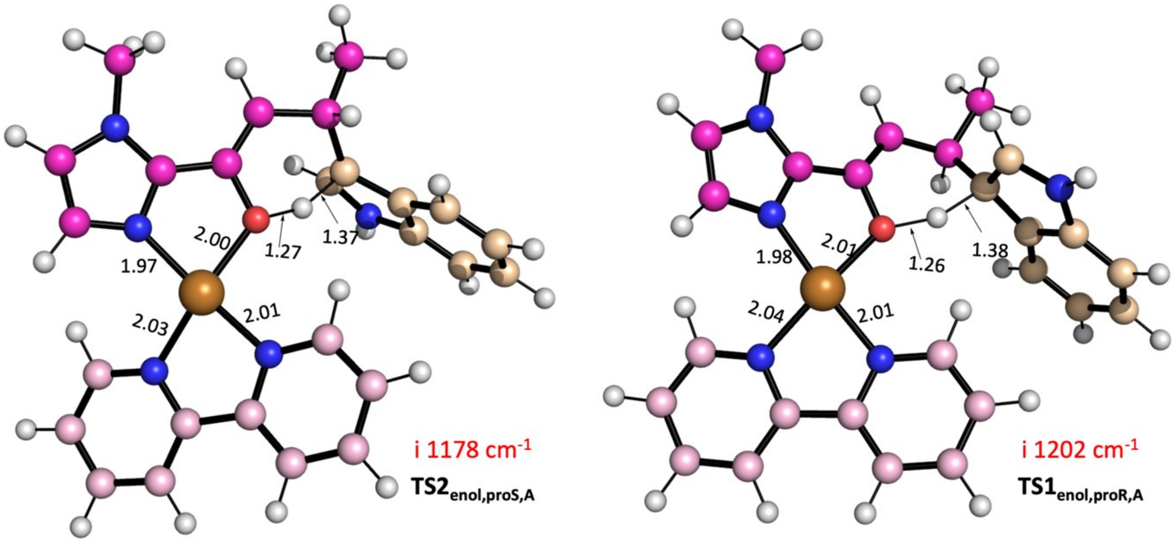

Figure S14: UB3LYP/BS1 optimized geometries of TS2$_{enol,proS,A}$ (left side) and TS2$_{enol,proR,A}$ (right side) structures as obtained in Gaussian 09. Bond lengths are in angstroms and the imaginary frequencies are in cm^{-1}.

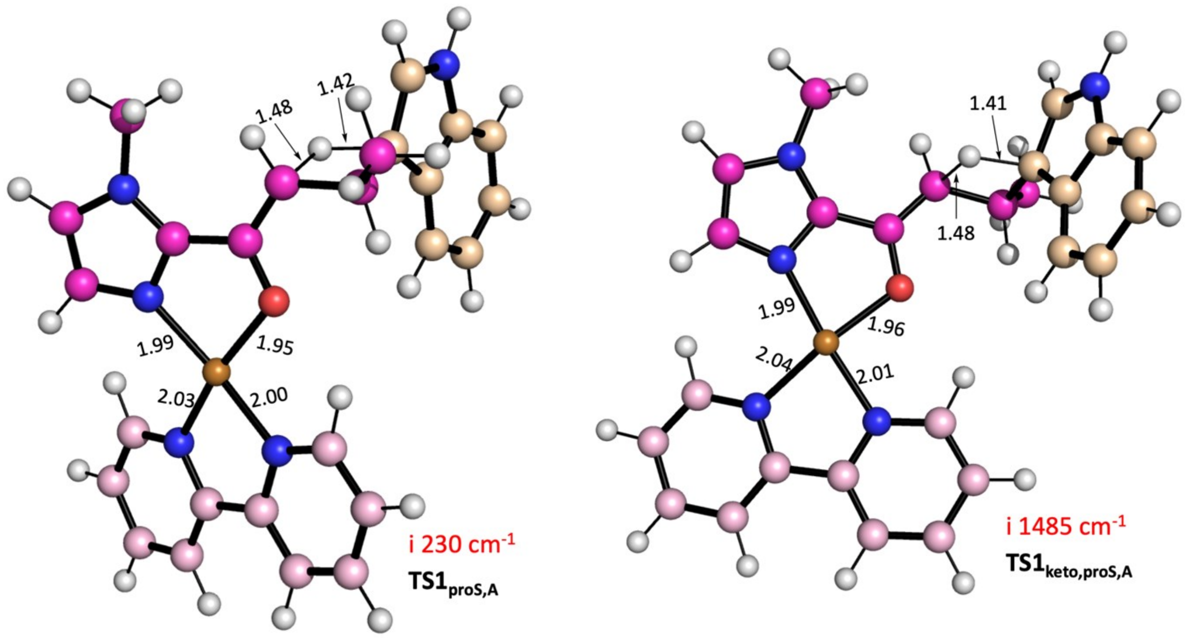

Figure S15: UB3LYP/BS1 optimized geometries of TS2$_{keto,proS,A}$ (left side) and TS2$_{keto,proR,A}$ (right side) structures as obtained in Gaussian 09. Bond lengths are in angstroms and the imaginary frequencies are in cm^{-1}.

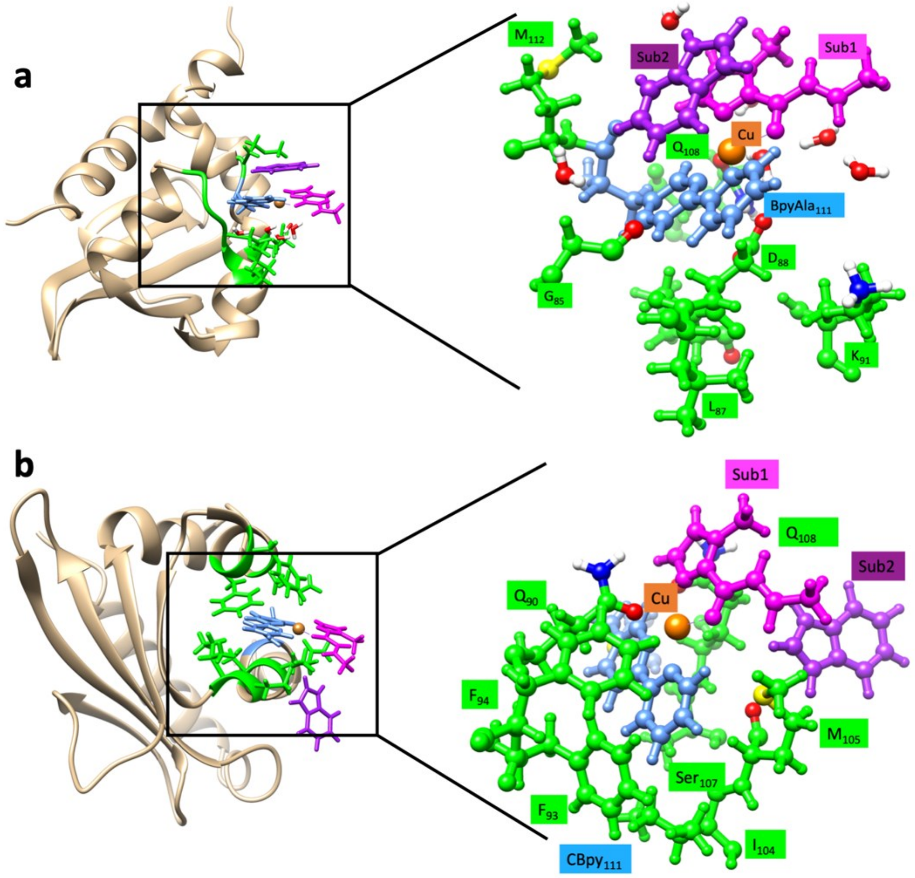

Figure S16: Extract from the MD equilibrated structure of SCP_Q111BpyAla complex (a) and SCP_Q111CBpy complex (b) with key active-site residues highlighted.

Table S1: Absolute (free) energies of the optimized geometries for the Friedel-Crafts alkylation by the Cu-Bpy model ^{2}**Re$_{A}$** calculates at the UB3LYP-D3/BS1//BS2 level of theory.

	E$_{BS1}$ (a.u.)	E$_{BS2}$ (a.u.)	E$_{ZPE}$ (a.u.)	E$_{G}$ (a.u.)	ΔE$_{total}$ + ZPE kcal mol^{-1}
**Re$_{A}$**	-1550.50833	-1551.05324	0.46760	0.40218	0.00
**TS1$_{proS,A}$**	-1550.49919	-1551.04190	0.46787	0.40450	7.28
**TS1$_{proR,A}$**	-1550.49776	-1551.04145	0.46793	0.40465	7.60
**Int1$_{proS,A}$**	-1550.51143	-1551.05332	0.46981	0.40699	1.34
**Int1$_{proR,A}$**	-1550.50958	-1551.05283	0.46991	0.40677	1.71
**TS2$_{keto,proS,A}$**	-1550.49299	-1551.04134	0.46507	0.40399	5.88
**TS2$_{keto,proR,A}$**	-1550.49025	-1551.04009	0.46486	0.40336	6.53
**TS2$_{enol,proS,A}$**	-1550.46388	-1551.00471	0.46442	0.40205	28.46
**TS2$_{enol,proR,A}$**	-1550.46246	-1551.00393	0.46446	0.40180	28.97
**Prod$_{keto,proS,A}$**	-1550.50119	-1551.05078	0.46851	0.40436	2.11
**Prod$_{keto,proR,A}$**	-1550.50210	-1551.04708	0.46780	0.40072	3.99
**Prod$_{enol,proS,A}$**	-1550.52695	-1551.05946	0.46808	0.40065	-3.60
**Prod$_{enol,proR,A}$**	-1550.52696	-1551.05972	0.46799	0.40014	-3.82

Table S2: Absolute (free) energies of the optimized geometries for the Friedel-Crafts alkylation by the SCP-Q111BpyAla model ^{2}**Re$_{B}$** calculates at the UB3LYP-D3/BS1//BS2 level of theory.

	E$_{BS1}$ (a.u.)	E$_{BS2}$ (a.u.)	E$_{ZPE}$ (a.u.)	E$_{G}$ (a.u.)	ΔE$_{total}$ + ZPE kcal mol^{-1}
**Re$_{proS,B}$**	-4440.32483	-4441.80462	1.50491	1.34108	0.00
**Re$_{proR,B}$**	-4440.31584	-4441.79328	1.50210	1.34095	5.35
**TS1$_{proS,B}$**	-4440.31810	-4441.78926	1.50560	1.34948	10.07
**TS1$_{proR,B}$**	-4440.29815	-4441.77774	1.50278	1.34375	15.53
**Int1$_{proS,B}$**	-4440.33133	-4441.80384	1.50675	1.34948	1.65
**Int1$_{proR,B}$**	-4440.30786	-4441.79052	1.50343	1.34176	7.92

Table S3: Absolute (free) energies of the optimized geometries for the Friedel-Crafts alkylation by the SCP-Q111CBpy model ^{2}**Re$_{C}$** calculates at the UB3LYP-D3/BS1//BS2 level of theory.

	E$_{BS1}$ (a.u.)	E$_{BS2}$ (a.u.)	E$_{ZPE}$ (a.u.)	E$_{G}$ (a.u.)	ΔE$_{total}$ + ZPE kcal mol^{-1}
**Re$_{proS,C}$**	-4843.43190	-4844.87956	1.54161	1.37942	5.64
**Re$_{proR,C}$**	-4843.44096	-4844.88643	1.53950	1.37691	0.00
**TS1$_{proS,C}$**	-4843.42329	-4844.86498	1.54262	1.37729	15.42
**TS1$_{proR,C}$**	-4843.42341	-4844.86779	1.54254	1.37457	13.61
**Int1$_{proS,C}$**	-4843.42564	-4844.87442	1.54317	1.37394	9.85
**Int1$_{proR,C}$**	-4843.43357	-4844.88063	1.54431	1.37872	6.66

Cartesian Coordinates.

Model A structures:

$^{2}Re_{A}$

H	-4.06274500	-3.62111800	2.92099700
H	-2.09985600	-2.30750100	2.18198400
C	-4.17523600	-2.86269800	2.15258300
C	-1.49945800	1.32353800	-1.32330100
C	-3.06948500	-2.12181800	1.73605400
C	-3.22963900	-1.14994400	0.74445000
C	-5.50696600	-1.63138900	0.59369900
C	-5.41829300	-2.62059900	1.57021400
C	-2.11293700	-0.33118700	0.20411000
C	-0.78479000	-0.46824000	0.61689200
C	0.20055800	0.32473700	0.03089900
C	-0.15831700	1.23539200	-0.96111100
N	-4.44522700	-0.91607100	0.19620400
N	-2.45012100	0.56789900	-0.75315100
H	-1.82289900	2.01157300	-2.09704500
H	-6.45010800	-1.40464400	0.10613400
H	-6.30008400	-3.18153900	1.86002700
H	-0.51333600	-1.18585200	1.38137700
H	1.23516500	0.22411100	0.34388300
Cu	-4.43261700	0.63291800	-1.19520200
O	-4.37212900	1.39840800	-3.19110500
N	-6.33490700	1.29967500	-1.42824400
C	-5.47707200	1.80216400	-3.63401000
C	-5.63765400	2.26866000	-5.00046000
C	-4.59095200	2.31365500	-5.85775000
C	-4.65947200	2.75830700	-7.27517900
H	-3.96357200	3.59103300	-7.44627900
H	-6.62025500	2.57076600	-5.34088900
H	-5.66382800	3.06707000	-7.57573600
H	-4.32478800	1.94742500	-7.93729800
H	-3.61947400	2.00186900	-5.47965800
C	-6.59363800	1.76145000	-2.66606600
N	-7.90927700	2.13646700	-2.74802000
C	-8.47399500	1.90381400	-1.52390400
C	-7.48763600	1.37841300	-0.71432900
H	-7.55256300	1.08305700	0.32254300
H	-9.51243800	2.12582600	-1.32635800
C	-8.65724100	2.69148300	-3.88633600
H	-8.22014200	3.64160100	-4.20029000
H	-8.66770400	1.98218800	-4.71653000
H	-9.68324000	2.86597200	-3.56254300
C	3.98954500	4.11848800	-3.70341400
C	4.45736600	2.87473300	-4.15930900
C	3.57722300	1.89058800	-4.63784300
C	2.20273100	2.11557000	-4.67585000
C	1.70063200	3.35490100	-4.22047400
C	2.61752400	4.33420700	-3.74359500
H	4.68005400	4.87495300	-3.34294800
H	5.52436600	2.67663100	-4.14534300
H	3.97747200	0.94569900	-4.99093700
H	1.52964500	1.35400200	-5.05942600
N	1.86882400	5.45011600	-3.37972800
H	2.24952300	6.31905000	-3.02933600
C	0.55003500	5.21404300	-3.61578300
C	0.39564300	3.92690000	-4.12645900
H	-0.53839900	3.47907800	-4.44125600
H	-0.19361700	5.97238100	-3.41391800
H	0.57591100	1.86359900	-1.45347500

$^{2}TS1_{ProS,A}$

H	-5.75359700	-3.36315700	3.48207800
H	-3.35691600	-2.90661900	3.04036000
C	-5.46571900	-2.71886800	2.65724700
C	-1.02219900	-0.21869900	-0.60715700
C	-4.11722500	-2.45860900	2.41183200
C	-3.76310500	-1.62890300	1.34675400
C	-6.01203700	-1.33003500	0.78622900
C	-6.43280300	-2.15235600	1.82772800
C	-2.36667300	-1.31177200	0.96754700
C	-1.23895800	-1.73440900	1.67238600
C	0.02770200	-1.37693200	1.20765900
C	0.14251900	-0.60906500	0.04937000
N	-4.71560800	-1.06790600	0.55903100
N	-2.23650500	-0.56195700	-0.15400200
H	-1.00993600	0.37987700	-1.51127100
H	-6.72253800	-0.87358100	0.10601500
H	-7.49011100	-2.34109200	1.97745200
H	-1.33696300	-2.32753700	2.57376700
H	0.91309400	-1.69554400	1.74893700
Cu	-3.96635100	0.08778000	-0.93885600
O	-3.18675900	0.71116200	-2.61896200
N	-5.47198400	1.26280400	-1.50091400
C	-3.91549300	1.54395300	-3.31706400
C	-3.51821300	2.02819900	-4.55520400
C	-2.24647400	1.73775100	-5.11877000
C	-1.70834500	2.59616900	-6.22764100
H	-1.28284000	3.50536400	-5.77971200
H	-4.20403700	2.63828500	-5.12990400
H	-2.48674700	2.90855900	-6.93014500
H	-0.90012100	2.10577500	-6.77577800
H	-1.50286900	1.35913900	-4.42311000
C	-5.18706000	1.88763000	-2.65744100
N	-6.17503000	2.78210200	-2.94325500
C	-7.10463900	2.70838700	-1.93001300
C	-6.65794300	1.76597800	-1.03887000
H	-7.10672700	1.45282800	-0.10869800
H	-7.98704200	3.33077500	-1.92381400
C	-6.27414100	3.71087200	-4.07442300
H	-5.36723800	4.31500600	-4.14321700
H	-6.43515400	3.16305300	-5.00618800
H	-7.12545000	4.36913800	-3.90072200
C	0.20554500	-0.77085400	-8.48641100
C	1.35588500	-1.14022800	-7.79119800
C	1.35343000	-1.26754100	-6.39041800
C	0.20325200	-1.02248600	-5.64223800
C	-0.96653700	-0.64828000	-6.31513200
C	-0.93639700	-0.53686500	-7.72370900
H	0.20427300	-0.68041300	-9.56826800
H	2.26854900	-1.33931900	-8.34341600
H	2.26655800	-1.56830000	-5.88653700
H	0.21350100	-1.13488900	-4.56167800
N	-2.23337700	-0.18364600	-8.13383900
H	-2.51157000	-0.02804700	-9.09567100
C	-3.04892500	-0.08454100	-7.07410200
C	-2.31374700	-0.29152700	-5.88291900
H	-2.76590500	-0.61940200	-4.95612400
H	-4.09527800	0.16074000	-7.19627000
H	1.11065300	-0.31181700	-0.33820500

^{2}TS1$_{ProR,A}$

H	-5.22277200	-3.86008300	2.97245200
H	-2.96700100	-2.84539900	2.80331500
C	-5.03203200	-3.07606500	2.24641300
C	-1.04805700	0.67119300	-0.34493700
C	-3.76400300	-2.50180100	2.15485600
C	-3.53418700	-1.49627000	1.21464600
C	-5.74750800	-1.62932200	0.47912200
C	-6.04178200	-2.64093600	1.38930000
C	-2.22045200	-0.84947400	0.99321900
C	-1.07236600	-1.11177500	1.74205800
C	0.11451400	-0.45105400	1.42246700
C	0.13191500	0.45341300	0.36142700
N	-4.53235200	-1.06455400	0.40233200
N	-2.18707600	0.03856100	-0.03016900
H	-1.10931200	1.35672200	-1.18239900
H	-6.49380600	-1.26441900	-0.21738000
H	-7.03608300	-3.07279900	1.41776900
H	-1.09517800	-1.81554300	2.56539600
H	1.01391100	-0.64392200	1.99881300
Cu	-3.96030000	0.39747100	-0.89989800
O	-3.22105100	1.39192600	-2.41570400
N	-5.66131700	1.20860500	-1.53479000
C	-4.09638100	1.96690700	-3.19907500
C	-3.75780500	2.52624600	-4.42314800
C	-2.45549000	2.41481300	-4.98126800
C	-2.26538900	2.60320100	-6.45967600
H	-1.22006200	2.77984000	-6.72355700
H	-4.51912800	3.03091900	-5.00471700
H	-2.88416500	3.41142200	-6.86100900
H	-2.56860900	1.67409400	-6.96262000
H	-1.81581600	1.66618900	-4.52228000
C	-5.46693800	1.92328000	-2.65726800
N	-6.64709400	2.48464300	-3.04456300
C	-7.60293700	2.11315800	-2.12516600
C	-6.98131400	1.32411600	-1.19161100
H	-7.40040900	0.86889500	-0.30752400
H	-8.62740100	2.44413400	-2.20757200
C	-6.92206700	3.34190300	-4.20230800
H	-6.26437400	4.21345200	-4.19020400
H	-6.79032100	2.78125800	-5.13096300
H	-7.95604800	3.68075900	-4.13702100
C	1.69713200	4.65244600	-6.13717600
C	2.62841400	3.65685700	-5.84586300
C	2.35298700	2.65446000	-4.89822000
C	1.13826900	2.61664500	-4.21610600
C	0.18597900	3.60703600	-4.48566200
C	0.49084100	4.60236400	-5.44138700
H	1.90972400	5.42808700	-6.86659800
H	3.58444600	3.65789200	-6.35937400
H	3.10509700	1.89835900	-4.69639300
H	0.94094500	1.83448200	-3.48806400
N	-0.61813800	5.46255600	-5.50521600
H	-0.68720900	6.26954700	-6.11411900
C	-1.56891800	5.06556200	-4.64745000
C	-1.17279300	3.86276100	-4.01572300
H	-1.55866100	3.55502300	-3.05307500
H	-2.49087700	5.62091600	-4.53995200
H	1.03691500	0.98311400	0.08578300

^{2}Int1$_{ProS,A}$

H	-5.22090000	-3.75098800	3.00712900
H	-2.96263800	-2.74061000	2.84721600
C	-5.02256100	-2.97558400	2.27388200
C	-1.00594700	0.72669800	-0.32750900
C	-3.75315900	-2.40355000	2.18744000
C	-3.51460800	-1.40903500	1.23732500
C	-5.71875400	-1.54803400	0.48338100
C	-6.02321000	-2.54912400	1.40228100
C	-2.19648000	-0.76801000	1.01953000
C	-1.05609500	-1.02654300	1.78239000
C	0.13678600	-0.37730700	1.46388500
C	0.16789200	0.51176500	0.38986800
N	-4.50323500	-0.98500100	0.41112000
N	-2.15093000	0.10593000	-0.01410100
H	-1.06098900	1.40048300	-1.17510200
H	-6.45782700	-1.18874500	-0.22364400
H	-7.01825100	-2.97950600	1.42638900
H	-1.08976400	-1.71900800	2.61485400
H	1.03039300	-0.56711700	2.05024200
Cu	-3.92068700	0.47100100	-0.91156800
O	-3.17538500	1.44441100	-2.41145600
N	-5.62584900	1.25955900	-1.54096200
C	-4.07409100	1.98551600	-3.23534100
C	-3.74416500	2.48217300	-4.45831300
C	-2.35425600	2.38081900	-5.01978400
C	-2.33551600	2.49843900	-6.54889600
H	-1.32149200	2.41928600	-6.95139000
H	-4.51113900	2.89775000	-5.09978600
H	-2.76805700	3.44784500	-6.88903400
H	-2.93088900	1.69255400	-6.98871600
H	-1.93134000	1.41296000	-4.72408400
C	-5.44026200	1.94529600	-2.68012000
N	-6.62336900	2.49028100	-3.07539400
C	-7.57846800	2.13588400	-2.14092800
C	-6.95023600	1.37666500	-1.19336400
H	-7.36020800	0.94301500	-0.29442500
H	-8.60423200	2.46038600	-2.23007000
C	-6.89761300	3.31382700	-4.25301400
H	-6.20737600	4.15968700	-4.28729600
H	-6.80542500	2.72046000	-5.16652500
H	-7.91713500	3.69269100	-4.17756200
C	1.76807900	4.91802600	-5.63791000
C	2.68613500	3.86573300	-5.63332100
C	2.31801100	2.58759600	-5.18712200
C	1.02323500	2.31534600	-4.72665300
C	0.09355700	3.34998100	-4.72066500
C	0.49244400	4.61476300	-5.17777200
H	2.04278500	5.90934000	-5.98455100
H	3.69775700	4.04079700	-5.98426500
H	3.05464800	1.79049400	-5.20350200
H	0.75851900	1.31638400	-4.39321300
N	-0.64233200	5.46601000	-5.07619500
H	-0.63911000	6.44611000	-5.35291300
C	-1.68544000	4.84001200	-4.60140800
C	-1.36168800	3.41988900	-4.31837100
H	-1.50490200	3.23274700	-3.24247700
H	-2.63707700	5.34045900	-4.46971300
H	1.07895200	1.03090500	0.11366400

^{2}Int1$_{ProR,A}$

H	-5.89957100	-3.46002800	3.30919900
H	-3.48621700	-3.04443400	2.92220400
C	-5.58253800	-2.81318200	2.49718600
C	-1.02556700	-0.35392100	-0.63848400
C	-4.22469400	-2.57620600	2.28243000
C	-3.83370800	-1.74193000	1.23356700
C	-6.06269900	-1.39379100	0.63101000
C	-6.52104800	-2.21921900	1.65444000
C	-2.42288100	-1.44563500	0.88846500
C	-1.31874500	-1.89627200	1.61386400
C	-0.03662300	-1.55293800	1.18344700
C	0.11768700	-0.77101700	0.03912800
N	-4.75766700	-1.15432000	0.43260200
N	-2.25463600	-0.68477800	-0.21946400
H	-0.98865400	0.25962800	-1.53224000
H	-6.74926700	-0.91333100	-0.05748100
H	-7.58465700	-2.38840400	1.78102200
H	-1.44661100	-2.49890400	2.50515200
H	0.83073600	-1.89229800	1.74116600
Cu	-3.95581500	0.01887500	-1.04004700
O	-3.14997800	0.64174300	-2.68349000
N	-5.42363300	1.24923000	-1.55377900
C	-3.85266900	1.55368900	-3.35846900
C	-3.43254100	2.05978500	-4.54903500
C	-2.11113400	1.69008800	-5.16215100
C	-1.68011000	2.67369300	-6.25663900
H	-1.58594500	3.67915500	-5.83495300
H	-4.05375200	2.76225100	-5.09006400
H	-2.41618200	2.72647100	-7.06872600
H	-0.71247200	2.40256700	-6.68840900
H	-1.35048800	1.67278100	-4.37156600
C	-5.11076600	1.91357500	-2.67847400
N	-6.06007500	2.85841000	-2.92258000
C	-6.99854600	2.77682500	-1.91128300
C	-6.59425900	1.77967300	-1.06753000
H	-7.05436300	1.44210800	-0.15169600
H	-7.85333200	3.43547300	-1.87903100
C	-6.10849300	3.84127200	-4.00633600
H	-5.17638900	4.40982600	-4.03832700
H	-6.27764400	3.34715100	-4.96674900
H	-6.93462100	4.52552500	-3.81181300
C	-0.13576800	-1.42942300	-8.36833000
C	1.16046100	-1.49848800	-7.85378900
C	1.45491200	-1.01398100	-6.57059700
C	0.46689800	-0.44671500	-5.75585000
C	-0.83142800	-0.36714200	-6.24853800
C	-1.09140200	-0.85845000	-7.53659900
H	-0.37310800	-1.80046500	-9.36045700
H	1.95049800	-1.93183100	-8.45812000
H	2.47407600	-1.07959100	-6.20288500
H	0.71829700	-0.07581200	-4.76705000
N	-2.47508600	-0.64688400	-7.78635800
H	-2.93680200	-0.90262500	-8.65722700
C	-3.07506600	-0.07722800	-6.77582400
C	-2.11827600	0.17467700	-5.67077400
H	-2.44449700	-0.39194800	-4.78218700
H	-4.12989900	0.16748600	-6.80548300
H	1.09964400	-0.48290700	-0.31925700

^{2}TS2$_{keto,ProS,A}$

H	-5.91846400	-3.84309600	2.47494600
H	-3.51259600	-3.65297200	1.91045100
C	-5.58804400	-3.11189800	1.74386000
C	-1.02311900	-0.73341900	-1.44627600
C	-4.23294600	-3.00165100	1.43006500
C	-3.82313400	-2.05675000	0.48800700
C	-6.03326900	-1.35791900	0.17706500
C	-6.50810100	-2.28349000	1.10262700
C	-2.42193100	-1.87552200	0.04345800
C	-1.33129600	-2.56686700	0.57209100
C	-0.05575800	-2.32374600	0.06030800
C	0.10407400	-1.39647500	-0.96831900
N	-4.72776100	-1.24224400	-0.11208600
N	-2.24813800	-0.97010300	-0.95256700
H	-0.95958200	0.00239900	-2.23976900
H	-6.70622000	-0.69552000	-0.35590400
H	-7.57074900	-2.34826600	1.30888400
H	-1.46470700	-3.28196300	1.37499000
H	0.80063800	-2.85411400	0.46489000
Cu	-3.90287000	0.05195000	-1.44784900
O	-3.18969500	0.86633200	-3.12547700
N	-5.24048400	1.49022300	-1.64491000
C	-3.90016400	1.95348600	-3.59883100
C	-3.52821300	2.54811000	-4.75070300
C	-2.30711300	2.09866000	-5.52414600
C	-2.30499600	2.62034500	-6.97207100
H	-2.33101300	3.71519200	-6.98592300
H	-4.09291700	3.38553500	-5.13933700
H	-3.17306600	2.25303100	-7.53037300
H	-1.39993400	2.29886500	-7.49524900
H	-1.43071400	2.52812600	-5.01504700
C	-5.01198200	2.27598900	-2.71209700
N	-5.89304000	3.31019000	-2.71284200
C	-6.70302600	3.16609600	-1.60236400
C	-6.28966800	2.04105900	-0.94520700
H	-6.66017300	1.62358700	-0.02202200
H	-7.48594900	3.87480700	-1.37843100
C	-5.99072400	4.41468600	-3.67146700
H	-5.03747200	4.94544600	-3.73011600
H	-6.27445300	4.03980000	-4.65814800
H	-6.75843700	5.10470400	-3.32125200
C	0.09270500	-2.30850500	-6.04412000
C	1.33863700	-1.89300300	-5.58113000
C	1.52509800	-0.60349700	-5.04722300
C	0.47031000	0.30352900	-4.95151900
C	-0.79340200	-0.09015300	-5.41212900
C	-0.94795100	-1.38408500	-5.95440400
H	-0.05132000	-3.29721400	-6.46906500
H	2.18377300	-2.57066800	-5.64780000
H	2.51714800	-0.30447100	-4.72242600
H	0.63894700	1.30133300	-4.55577300
N	-2.28429600	-1.49808600	-6.36579500
H	-2.68756400	-2.32500200	-6.79161900
C	-2.95293400	-0.36574000	-6.12176200
C	-2.11477900	0.56641800	-5.44429100
H	-2.62923200	0.44274300	-4.17581200
H	-3.99429900	-0.26289200	-6.40006100
H	1.07713300	-1.18302000	-1.39608700

^{2}TS2$_{keto,ProR,A}$

H	-4.43601200	-3.30407600	2.89971900
H	-2.28259400	-2.12493200	2.56378600
C	-4.34764900	-2.54224400	2.13154000
C	-0.79717800	1.36237600	-0.83280200
C	-3.13661200	-1.87473400	1.94599900
C	-3.03795600	-0.89840100	0.95330900
C	-5.26964000	-1.23929800	0.34754800
C	-5.43195600	-2.22910000	1.31345500
C	-1.79257100	-0.16808700	0.62582800
C	-0.58512200	-0.33617700	1.30556400
C	0.54108900	0.37213300	0.88654600
C	0.43819300	1.23251700	-0.20528000
N	-4.10969500	-0.58465300	0.18309300
N	-1.88223900	0.68528100	-0.42645900
H	-0.92654000	2.01631900	-1.68609600
H	-6.07801400	-0.96445600	-0.31996200
H	-6.38463500	-2.73732300	1.41384500
H	-0.51483500	-1.01080400	2.15023600
H	1.48563600	0.24679600	1.40660600
Cu	-3.73399600	0.86875400	-1.19560400
O	-3.24646700	1.76962300	-2.91970500
N	-5.54274200	1.44751900	-1.74018500
C	-4.32617300	2.09649300	-3.71645500
C	-4.11922700	2.47530400	-4.99507300
C	-2.72325900	2.53197900	-5.57741200
C	-2.66698300	3.29829900	-6.90983200
H	-1.64807200	3.30505800	-7.30721800
H	-4.95548500	2.71130000	-5.64059300
H	-2.99404600	4.33691900	-6.78847600
H	-3.31508900	2.82219800	-7.65344000
H	-2.41222900	1.49430500	-5.77192600
C	-5.57650700	1.96693500	-2.97963500
N	-6.85264300	2.30770400	-3.29726900
C	-7.64713900	1.99618800	-2.21044900
C	-6.82894700	1.46752800	-1.25068300
H	-7.08000200	1.14287900	-0.25277000
H	-8.70892000	2.19158000	-2.21224800
C	-7.34299000	2.91040700	-4.53999400
H	-6.78317700	3.82250400	-4.75959300
H	-7.25037100	2.20422300	-5.36922100
H	-8.39458300	3.16442800	-4.40633000
C	1.77780500	3.75273300	-3.63019400
C	2.46287100	2.59782500	-3.99979200
C	1.79259400	1.51061200	-4.59392800
C	0.41948800	1.54267200	-4.82825600
C	-0.29319800	2.69541800	-4.47143200
C	0.40568000	3.77404700	-3.88559900
H	2.29340900	4.59979100	-3.18767200
H	3.53515600	2.54138200	-3.84127400
H	2.36281400	0.63404300	-4.88543600
H	-0.07744300	0.69723900	-5.29516900
N	-0.53087600	4.79431000	-3.65825200
H	-0.31335700	5.69736000	-3.25190700
C	-1.74724400	4.41847800	-4.06848900
C	-1.71959700	3.07193400	-4.53175500
H	-2.32457400	2.42506800	-3.47923300
H	-2.59083800	5.09353100	-3.99863200
H	1.29079400	1.79210500	-0.57318300

^{2}TS2$_{enol,ProS,A}$

H	-5.26729500	-3.81083100	3.08914400
H	-2.95973300	-2.92097400	2.91545800
C	-5.01384200	-3.10602400	2.30346400
C	-0.77060400	0.22254100	-0.44742900
C	-3.71656300	-2.60195200	2.20889600
C	-3.40563600	-1.69709000	1.19259500
C	-5.59808600	-1.79386200	0.38934100
C	-5.97225500	-2.70376000	1.37405100
C	-2.05581900	-1.13074200	0.96740600
C	-0.94803800	-1.37441800	1.77996600
C	0.27789300	-0.79271900	1.45206600
C	0.37304300	0.01682400	0.32081300
N	-4.35432500	-1.29576200	0.30805500
N	-1.94616900	-0.33715200	-0.12606000
H	-0.77136200	0.84073400	-1.33826800
H	-6.30205100	-1.45838900	-0.36381200
H	-6.98735600	-3.08396200	1.40446000
H	-1.03108200	-2.00269100	2.65870700
H	1.14705500	-0.97200600	2.07720000
Cu	-3.67135600	0.02052400	-1.08553900
O	-2.82055300	0.86775000	-2.63077700
N	-5.29534400	0.89709500	-1.83779300
C	-3.59605000	1.57122400	-3.40529800
C	-3.12947300	2.17792000	-4.57784200
C	-1.64979600	2.12761700	-5.00697600
C	-1.02232300	3.48983900	-5.30540500
H	-0.97218600	4.09320800	-4.39312900
H	-3.71845400	2.98108100	-5.01237100
H	-1.60298700	4.05732800	-6.04219000
H	-0.00502300	3.37481000	-5.69280600
H	-1.06560500	1.60955300	-4.24234100
C	-4.99787900	1.58825100	-2.95342100
N	-6.11793900	2.21836900	-3.40716200
C	-7.14209700	1.91719100	-2.53809600
C	-6.62100600	1.10100800	-1.56633600
H	-7.11552800	0.68233700	-0.70346800
H	-8.13905000	2.30709000	-2.67945900
C	-6.27194800	3.08012900	-4.58335400
H	-5.69304800	3.99901800	-4.45994600
H	-5.95314300	2.54956200	-5.48268900
H	-7.32642900	3.33848300	-4.68098900
C	-1.23127500	-1.95509800	-8.02672800
C	-0.90412100	-2.87711400	-7.03622200
C	-0.84766300	-2.50181800	-5.68088700
C	-1.12418200	-1.19753900	-5.27526100
C	-1.44526000	-0.25073900	-6.25389100
C	-1.48879300	-0.65083100	-7.60668800
H	-1.27360400	-2.24042200	-9.07327300
H	-0.68522500	-3.90254700	-7.31595400
H	-0.57946100	-3.24769900	-4.93888000
H	-1.09203800	-0.92257800	-4.22503200
N	-1.81078700	0.48348700	-8.36318400
H	-1.92974000	0.48979200	-9.36979600
C	-1.96098800	1.55589800	-7.57498400
C	-1.81611500	1.17423600	-6.20924600
H	-3.13729500	1.24081500	-5.71372300
H	-2.18037300	2.52855500	-7.99464000
H	1.30924900	0.48430400	0.03645900

$^{2}TS2_{enol,ProR,A}$

H	-5.42202400	-2.98239400	3.59107600
H	-3.04738100	-2.77313100	2.91001800
C	-5.15288900	-2.36804200	2.73763000
C	-0.82985800	-0.37592600	-0.99886100
C	-3.81635200	-2.24682500	2.35741100
C	-3.48612700	-1.45506300	1.25639800
C	-5.73522200	-0.92286300	0.92130000
C	-6.13366300	-1.70421200	2.00223900
C	-2.11269000	-1.30507700	0.72427600
C	-0.98095300	-1.91004300	1.27241600
C	0.25544800	-1.73195400	0.65021600
C	0.33543100	-0.95877600	-0.50719100
N	-4.44773100	-0.78975700	0.56626000
N	-2.01365500	-0.54238900	-0.39183000
H	-0.84438600	0.23217100	-1.89592000
H	-6.46061900	-0.39838300	0.31073800
H	-7.18531500	-1.78719600	2.25324800
H	-1.05342700	-2.51600500	2.16756200
H	1.14316200	-2.19895900	1.06520000
Cu	-3.72217600	0.33254200	-0.97632800
O	-2.96685900	1.00245900	-2.66170100
N	-5.23002400	1.53082600	-1.49598300
C	-3.75920800	1.72879800	-3.39269300
C	-3.40862400	2.17639100	-4.67369200
C	-2.06991100	1.81765600	-5.34552500
C	-2.18127900	1.02856500	-6.65117800
H	-1.19708600	0.89064000	-7.11012700
H	-4.21594500	2.44592000	-5.35126700
H	-2.82785900	1.53129500	-7.37989600
H	-2.60844500	0.03891200	-6.45978200
H	-1.45542000	1.26290300	-4.63209800
C	-5.01682200	2.07341700	-2.70834300
N	-6.05544900	2.89589100	-3.02699700
C	-6.94178400	2.86801700	-1.97426500
C	-6.41985500	2.02132700	-1.02955300
H	-6.81888200	1.77323000	-0.05833000
H	-7.85022500	3.45165100	-1.98137100
C	-6.24469800	3.70294300	-4.23580700
H	-5.35766500	4.31214700	-4.41883500
H	-6.44500900	3.06006400	-5.09699200
H	-7.10057600	4.35925200	-4.07767700
C	0.99056800	5.86265200	-4.80665700
C	1.64942400	5.44247900	-3.65443400
C	1.30783800	4.23371400	-3.01986300
C	0.29385400	3.41563200	-3.51386800
C	-0.37867900	3.81199000	-4.67434900
C	-0.01140400	5.02530800	-5.29498300
H	1.25263100	6.79338900	-5.30022000
H	2.44262800	6.05694900	-3.24114200
H	1.85020500	3.93591100	-2.12784000
H	0.03490200	2.48934200	-3.00936200
N	-0.81986000	5.17050200	-6.43004400
H	-0.78711700	5.96198400	-7.06260100
C	-1.65617800	4.13283200	-6.55548900
C	-1.49962800	3.24854700	-5.44675600
H	-2.68343500	3.46587800	-4.71584300
H	-2.33497400	4.06359900	-7.39517100
H	1.27738200	-0.80891800	-1.02296300

$^{2}Prod_{keto,ProS,A}$

H	-6.02827000	-4.09887300	2.27812100
H	-3.59679400	-3.71685900	2.00368600
C	-5.67364100	-3.30727900	1.62548000
C	-0.93877400	-0.40988500	-0.81772800
C	-4.30357700	-3.08987400	1.47341800
C	-3.86246500	-2.06681800	0.63134200
C	-6.06879800	-1.50024200	0.10724800
C	-6.57581700	-2.50377300	0.92971100
C	-2.43300900	-1.75390600	0.38189400
C	-1.37025100	-2.39640100	1.02024100
C	-0.06104000	-2.02085000	0.71813600
C	0.16334600	-1.01085900	-0.21674800
N	-4.75221200	-1.28832100	-0.03172100
N	-2.19871000	-0.77265100	-0.52629700
H	-0.82504100	0.38386500	-1.54898900
H	-6.72483300	-0.84898200	-0.46145800
H	-7.64739100	-2.64604600	1.01680800
H	-1.55248800	-3.17455400	1.75152000
H	0.77175300	-2.51114600	1.21253800
Cu	-3.84728600	0.14175300	-1.20993000
O	-3.06366000	0.95152000	-3.04601700
N	-5.17507000	1.53927300	-1.62664900
C	-3.84896100	1.96272800	-3.62057800
C	-3.54212200	2.48132900	-4.82224100
C	-2.31047000	2.12433400	-5.63431100
C	-2.38288800	2.74302600	-7.04982200
H	-2.48766700	3.83175800	-6.98944200
H	-4.19567600	3.23810700	-5.23775200
H	-3.23315100	2.34139600	-7.61094700
H	-1.47144300	2.52090000	-7.61074800
H	-1.45232300	2.58574300	-5.12178600
C	-4.94999800	2.28755700	-2.72084400
N	-5.84399900	3.31276100	-2.76338900
C	-6.66075300	3.19652400	-1.65589900
C	-6.23598900	2.10081700	-0.95689500
H	-6.61065700	1.70178600	-0.02665800
H	-7.45776500	3.89944100	-1.46630900
C	-5.95189600	4.39093900	-3.75142800
H	-4.98669300	4.88857400	-3.86935800
H	-6.29547800	3.99910500	-4.71233500
H	-6.67910400	5.11619600	-3.38624000
C	0.13609100	-2.26117800	-6.17577600
C	1.42751300	-1.75838900	-5.99151000
C	1.64972700	-0.40258700	-5.69013700
C	0.58815600	0.48979600	-5.56232200
C	-0.72509600	0.01214500	-5.74369500
C	-0.91993000	-1.35977600	-6.05159400
H	-0.02853600	-3.30596700	-6.42154800
H	2.27534000	-2.42820000	-6.09444300
H	2.66741300	-0.04563700	-5.56824200
H	0.77746700	1.53722000	-5.34417100
N	-2.28874300	-1.55580900	-6.19739300
H	-2.72807000	-2.42838400	-6.45918500
C	-2.94701800	-0.37911700	-6.00737900
C	-2.02901200	0.63394800	-5.70762100
H	-2.60650700	0.47087900	-3.78955000
H	-4.02039600	-0.31784800	-6.12845500
H	1.16602800	-0.68814600	-0.47421300

^{2}Prod$_{keto,ProR,A}$

H	-3.15895100	-4.66661600	0.96621300
H	-1.81481000	-2.85148200	1.97402000
C	-3.30188400	-3.64084400	0.64100400
C	-1.65939100	2.16877900	1.24555400
C	-2.54512000	-2.61656500	1.20937800
C	-2.74222800	-1.29948800	0.78402800
C	-4.38220800	-1.99745400	-0.71920500
C	-4.23992700	-3.33073600	-0.34195100
C	-1.99324100	-0.13782500	1.33783500
C	-1.05508000	-0.24933500	2.36859900
C	-0.41097800	0.89466100	2.83768600
C	-0.71529500	2.13000300	2.26825300
N	-3.65478000	-1.01095700	-0.17442700
N	-2.27973800	1.06888900	0.79042400
H	-1.93738900	3.10674700	0.77477300
H	-5.10117300	-1.70262900	-1.47735800
H	-4.85104800	-4.09705600	-0.80609300
H	-0.82948100	-1.21110800	2.81281800
H	0.31470800	0.81875100	3.64146400
Cu	-3.77983400	1.00127000	-0.60260200
O	-3.22119600	1.43019200	-3.01735600
N	-5.17250600	2.15673900	-1.38554400
C	-4.13761800	2.26822300	-3.60322400
C	-4.02352500	2.68528600	-4.89397000
C	-2.96611800	2.21481000	-5.87399900
C	-3.45993900	2.31811200	-7.32839600
H	-2.68686100	1.97588900	-8.02194700
H	-4.72813000	3.41648300	-5.26717600
H	-3.72016800	3.34954300	-7.59155500
H	-4.34975100	1.69668100	-7.47183100
H	-2.73522900	1.16081500	-5.67249000
C	-5.21523900	2.56593800	-2.67223600
N	-6.39593300	3.22465500	-2.90275000
C	-7.09574000	3.23616400	-1.72004700
C	-6.33204600	2.57375000	-0.79242000
H	-6.55010400	2.37816500	0.24675300
H	-8.06421100	3.70702600	-1.64097900
C	-6.91465700	3.80117500	-4.14624900
H	-6.33776600	4.68352600	-4.43598200
H	-6.89486400	3.05628400	-4.94503500
H	-7.94830800	4.10217700	-3.97482500
C	1.68282100	3.58730800	-4.46394000
C	2.12476000	2.31695800	-4.06060900
C	1.29342800	1.18851400	-4.14670500
C	-0.00835400	1.29240000	-4.63535100
C	-0.48542000	2.55996600	-5.03444500
C	0.38320500	3.68177100	-4.93856200
H	2.34226800	4.44833600	-4.41444000
H	3.13855300	2.20635500	-3.68933000
H	1.67941600	0.21925900	-3.84840800
H	-0.62312700	0.40078800	-4.73833300
N	-0.32107300	4.78379600	-5.42764000
H	0.05461100	5.71923100	-5.52486100
C	-1.55748500	4.40445300	-5.81773500
C	-1.71950100	3.02138100	-5.60289000
H	-2.35855700	1.49843200	-3.46207600
H	-2.24978900	5.11973200	-6.24010300
H	-0.24189100	3.04449500	2.60843700

^{2}Prod$_{enol,ProS,A}$

H	-2.64880800	-4.89720200	2.15783400
H	-2.19056500	-2.72684900	3.24989700
C	-2.87430600	-3.97055400	1.63935100
C	-2.80500700	2.09138700	1.80242600
C	-2.61740600	-2.74649200	2.25473100
C	-2.91379200	-1.55902700	1.57914900
C	-3.68913200	-2.76892800	-0.25966000
C	-3.42078300	-3.98745100	0.35732300
C	-2.67480100	-0.20780000	2.15698000
C	-2.15900300	-0.00010200	3.44007300
C	-1.96635100	1.30283900	3.89789100
C	-2.29265500	2.37305900	3.06667400
N	-3.44650800	-1.58916400	0.33269800
N	-2.98961300	0.83995800	1.35974800
H	-3.07785600	2.88852700	1.11716700
H	-4.11288600	-2.72619700	-1.25786200
H	-3.63740800	-4.91788200	-0.15581700
H	-1.91025000	-0.83567900	4.08285300
H	-1.56759300	1.47578300	4.89259200
Cu	-3.80718600	0.23353600	-0.47300400
O	-2.90629300	0.15021100	-2.62857900
N	-4.88965000	1.55733600	-1.53001200
C	-3.43661200	0.97219500	-3.38085900
C	-2.96517200	1.14581800	-4.81712700
C	-1.65207100	0.38255700	-5.13422700
C	-0.43649100	1.05013500	-4.46653000
H	-0.55242500	1.05702100	-3.38007300
H	-2.83958700	2.21436700	-5.03342700
H	-0.31181800	2.08787300	-4.79889100
H	0.48340700	0.50537800	-4.69893000
H	-1.76066400	-0.62431400	-4.71262700
C	-4.55366000	1.75539300	-2.81775900
N	-5.38314900	2.70092700	-3.36851900
C	-6.25260000	3.09533400	-2.38942800
C	-5.93427800	2.37993000	-1.25257800
H	-6.40272900	2.41577600	-0.27994000
H	-7.01837600	3.83476700	-2.57278000
C	-5.40484100	3.24080300	-4.73388300
H	-4.49708800	3.81457500	-4.93429600
H	-5.50865700	2.43461500	-5.46207300
H	-6.26541900	3.90392400	-4.82181700
C	-2.00582500	-1.27996900	-9.88383200
C	-2.84745200	-2.38012400	-9.62022800
C	-3.28685600	-2.66967200	-8.32204800
C	-2.90629500	-1.87087900	-7.24176400
C	-2.07022000	-0.75798000	-7.47521700
C	-1.63746600	-0.49665200	-8.80339100
H	-1.66538500	-1.07019300	-10.89315200
H	-3.15300900	-3.01642700	-10.44452200
H	-3.92623700	-3.53027400	-8.15581600
H	-3.24183000	-2.11348700	-6.23757500
N	-0.81408500	0.63071800	-8.74681000
H	-0.33449700	1.04452000	-9.53715900
C	-0.71768600	1.06323500	-7.47251600
C	-1.48791400	0.23734800	-6.62561900
H	-3.77994900	0.80219400	-5.47169700
H	-0.10474800	1.91808800	-7.22343700
H	-2.15758000	3.40103500	3.38482500

$^{2}Prod_{enol,ProR,A}$

H	-5.81358800	-4.93452900	1.27103600
H	-3.56583200	-4.11343000	1.89492400
C	-5.48008000	-3.95781600	0.93466300
C	-1.04418900	0.18373000	0.92776100
C	-4.21252800	-3.49443300	1.28508700
C	-3.79666600	-2.23382100	0.84543500
C	-5.82993300	-1.90822400	-0.24548000
C	-6.30841300	-3.15401900	0.15330800
C	-2.46265400	-1.65443600	1.16357700
C	-1.48862700	-2.33044700	1.90432200
C	-0.26449300	-1.71299000	2.15616000
C	-0.03287100	-0.43119900	1.66019400
N	-4.61123200	-1.46162700	0.08847700
N	-2.22523000	-0.40712000	0.68807800
H	-0.91466000	1.18029200	0.51800200
H	-6.43616800	-1.24184200	-0.85200600
H	-7.30070600	-3.47797500	-0.14096300
H	-1.67333100	-3.32793200	2.28358800
H	0.49653700	-2.23009600	2.73216200
Cu	-3.75861600	0.40007500	-0.36112400
O	-2.77531700	1.29265500	-2.26290000
N	-4.99389300	1.89130000	-0.91211800
C	-3.46160700	2.18705800	-2.76642100
C	-3.03972000	2.86961100	-4.05910200
C	-1.85110800	2.16983000	-4.76921000
C	-2.27838100	0.83164700	-5.39793200
H	-1.43339200	0.34999400	-5.89882000
H	-3.89907500	2.93110400	-4.73857500
H	-3.07926600	0.96796800	-6.13480300
H	-2.64254300	0.14585200	-4.62907100
H	-1.10024900	1.95823000	-3.99799500
C	-4.68818200	2.56264200	-2.03756700
N	-5.64202100	3.52149500	-2.27387700
C	-6.56047300	3.43500100	-1.26471600
C	-6.14792900	2.41767700	-0.42721000
H	-6.61380700	2.05970400	0.47921800
H	-7.41648700	4.09209400	-1.21638800
C	-5.73487300	4.49358000	-3.37061400
H	-4.82483500	5.09308900	-3.42743600
H	-5.91073400	3.98405700	-4.32078300
H	-6.57678000	5.15491200	-3.16608400
C	0.94603200	5.80028900	-6.87781600
C	1.59019400	6.22940600	-5.69962500
C	1.32471700	5.62961800	-4.46178400
C	0.40540300	4.58407400	-4.35441100
C	-0.26085300	4.13709200	-5.51550300
C	0.03650800	4.76331500	-6.75602900
H	1.16545400	6.26764700	-7.83284800
H	2.31096700	7.03841600	-5.76029700
H	1.84625900	5.98019100	-3.57736600
H	0.21871000	4.11767300	-3.39129400
N	-0.73335000	4.12126500	-7.72879300
H	-0.71798100	4.33192000	-8.71931600
C	-1.47125100	3.14591800	-7.15802300
C	-1.22497600	3.10818800	-5.76935000
H	-2.78117700	3.90925200	-3.80855800
H	-2.11943900	2.52119900	-7.75645400
H	0.90484200	0.08468900	1.83483700

Model B structures:

$^{2}Re_{proS,B}$

C	22.08898400	29.09695000	9.69391200
H	21.80758000	28.79021000	10.70641300
H	23.02871900	29.66217300	9.70542300
C	20.97427300	29.90921000	9.09011100
O	21.02909900	30.98334500	8.56803700
C	18.67568300	28.10309900	13.56914000
H	18.11878700	28.62319400	12.78008000
C	17.72202200	27.24104700	14.43090800
H	17.37417600	26.40685500	13.80770000
H	18.31048700	26.79404700	15.24142800
C	16.49467800	27.95677800	15.02191100
H	16.80435200	28.78142600	15.67695000
C	15.49948500	28.47781300	13.97627200
H	15.15707500	27.67063600	13.31788200
H	14.61502800	28.90803100	14.45971000
H	15.93328200	29.25694700	13.33517200
C	19.46591000	29.08762400	14.41753400
O	20.53040600	28.79441600	14.95337700
N	18.89252500	30.33100300	14.55120700
H	17.92834100	30.43653700	14.26021300
C	19.35511800	31.29126900	15.55141800
H	20.39835500	31.05459800	15.74715300
C	19.21988300	32.70112300	14.98267000
H	19.75999000	32.75773300	14.02962200
H	18.16923900	32.89932100	14.74586600
C	19.74197500	33.80859900	15.89101100
O	19.52649500	35.02575800	15.46187600
O	20.33248900	33.53345500	16.94104200
C	18.47796800	31.11919200	16.80821000
O	17.27651000	31.44473100	16.73379400
N	19.00892600	30.53458200	17.89041300
C	20.43501600	30.18960900	18.09750200
H	21.06040900	31.06716900	17.91133500
H	20.73232800	29.39219100	17.40817600
C	20.48076800	29.73342000	19.56523400
H	20.66348000	30.59352300	20.22083000
H	21.27512500	29.00287900	19.74194300
C	19.07140000	29.17291700	19.81817900
H	18.81023300	29.13326800	20.87919000
H	18.99004400	28.15732700	19.41313400
C	18.16695500	30.13469400	19.03751700
H	17.24625400	29.67407000	18.66889400
N	16.24408000	32.11261400	21.31973400
H	16.43238600	33.03796800	21.70511300
C	14.98934400	32.14997800	20.55461700
H	14.14065000	32.57007200	21.12350800
C	15.17597200	32.94372300	19.25742400
H	15.54526800	33.95110900	19.50601400
H	15.95251700	32.45882900	18.65548000
C	13.87097400	33.08194900	18.46201500
H	13.46917000	32.08800600	18.21873500
H	13.11363800	33.56599300	19.09119800
C	14.01213200	33.90728700	17.17121700
H	13.01879900	34.24302100	16.83969000
H	14.59898900	34.81293700	17.38514000
C	14.67102200	33.12315000	16.03934800
H	15.64797200	32.70883100	16.30167600
H	14.03217200	32.30179300	15.70212400
N	14.88400600	34.02439000	14.84520600
H	15.60920900	34.75348000	15.02006400

H	15.26105000	33.54100700	13.99838800
H	14.01319400	34.49650200	14.58305400
C	24.84774100	33.90301200	20.50697900
H	24.24762000	33.26021200	19.84843800
C	23.99012500	34.95715300	21.22403700
H	23.18968400	34.43653300	21.76332800
H	24.59456600	35.47376600	21.97674500
C	23.39041100	36.03670500	20.28689800
H	22.68907200	36.65355900	20.86193800
H	24.19825100	36.68382400	19.93121100
C	22.70721000	35.47601500	19.04419800
O	23.32575500	35.33569300	17.98152800
N	21.37988900	35.16628600	19.12742400
H	20.95981400	35.05113300	20.04193800
H	20.97860700	34.61440500	18.35417500
C	26.00062200	34.48137500	19.68831700
O	26.49518400	34.00931600	18.71454000
C	26.38627900	33.08396800	15.33536900
C	26.65403400	34.53392500	14.93020500
H	20.43755800	34.77571100	8.27177800
H	22.10025200	33.70108300	9.76919600
O	25.89227200	35.44421000	15.28789300
C	24.90544200	32.84371600	15.68679900
C	20.43276800	34.99858100	9.33441800
C	22.92987700	34.12983800	14.78434100
C	21.35936000	34.38357200	10.17478500
C	21.33303300	34.68188200	11.54151800
C	19.55696300	36.16604800	11.22972500
C	19.51033800	35.90260000	9.86381900
C	22.25933400	34.09178300	12.53561300
C	23.23242300	33.13473300	12.24270700
C	24.07462000	32.69560800	13.26605900
C	23.96024500	33.20621900	14.56333600
N	20.43977400	35.56908200	12.04140900
N	22.12083300	34.54979000	13.80463900
H	26.71394700	32.39102500	14.55406300
H	24.78334100	31.78326100	15.94259000
H	24.64990600	33.42600900	16.57453800
H	22.76535400	34.54530700	15.77483000
H	18.88908600	36.87814100	11.70382600
H	18.78379000	36.40530400	9.23431000
H	23.35073000	32.74687000	11.23503400
H	24.83742700	31.95612200	13.03729500
N	27.74834600	34.79821000	14.17785500
H	27.82867700	35.76571600	13.88024700
C	28.72430900	33.86550000	13.61581500
H	28.79550100	32.99758900	14.27505100
C	28.41662200	33.40896400	12.17471400
H	28.95668900	32.46951700	11.99109000
H	27.34812600	33.17032700	12.09475400
C	28.83290800	34.38493900	11.06413300
H	29.91890600	34.53020300	11.08858100
H	28.59283300	33.96177100	10.08226500
S	28.15988200	36.09493600	11.13889600
C	26.39392600	35.78933300	10.78587200
H	25.91892000	36.77011500	10.69675500
H	26.27360700	35.25655000	9.83703800
H	25.90376700	35.23222700	11.59029200
Cu	20.74392700	35.99399900	14.05795200
O	19.55289400	37.61247800	14.10475100
N	22.01740500	37.35984700	14.97776000
C	20.02168800	38.64022300	14.68987300
C	19.23523900	39.85336500	14.77820500
C	18.05305900	39.95096300	14.13904200
C	17.17606900	41.15510500	14.13955000
H	17.00713500	41.50633100	13.11273600
H	19.60979300	40.68576400	15.35901900
H	17.59318300	41.97540000	14.72920900
H	16.18954900	40.90032300	14.54737700
H	17.71309300	39.09501500	13.55953600
C	21.37203100	38.52021900	15.20390000
N	22.20510000	39.37710100	15.88678800
C	23.37887200	38.71627500	16.08646200
C	23.25283200	37.46053800	15.50685200
H	23.99550500	36.67776500	15.46284700
H	24.20958400	39.12680300	16.64050400
C	21.95027400	40.74130500	16.36596400
H	22.85252600	41.09054700	16.86854600
H	21.73956400	41.40495300	15.52444600
H	21.12265200	40.74019900	17.07956500
C	16.47820100	37.93267200	16.94900000
C	16.12181900	39.25823000	17.28538500
C	16.98414900	40.06861300	18.01275600
C	18.22655600	39.54943000	18.42380500
C	18.55933400	38.20640100	18.07884400
C	17.69761300	37.38697900	17.33917200
C	19.32827700	40.06704600	19.18779700
C	20.25408700	39.05689500	19.28674400
N	19.80674300	37.93991000	18.60720000
H	15.77115100	37.31852800	16.39573600
H	15.14917200	39.64082900	16.98764000
H	16.69570300	41.08189100	18.27921300
H	17.98199200	36.37237600	17.07962700
H	19.40425800	41.04651900	19.64021300
H	21.20388600	39.03766100	19.80450200
H	20.27419900	37.03745600	18.60381100
O	23.54070500	32.19659200	9.08001900
H	22.75221000	31.80254400	8.65807500
H	24.16830400	32.35804300	8.35858000
O	16.50078800	33.55281300	12.69545900
H	16.89307500	34.41337200	12.95730600
H	16.34729500	33.58505400	11.73925600
O	17.15812300	35.62186300	14.43162400
H	17.22720900	36.58979000	14.49092500
H	18.03006800	35.31806800	14.83658500
O	25.47035800	37.20542300	17.59061200
H	26.08504200	36.79275700	16.95987900
H	24.81775100	36.49250700	17.73211400
H	22.20369100	28.18003400	9.10407200
H	26.98981500	32.86373300	16.22513600
H	25.32118800	33.25150400	21.25434500
H	16.13481900	31.49633400	22.12388700
H	14.71872200	31.11526100	20.30991400
H	19.40523800	27.44648800	13.08627600
H	29.70401500	34.35514700	13.65107300
H	17.88368800	31.00657200	19.63964500
H	15.97457900	27.24063100	15.67050400

$^{2}Re_{proR,B}$

C	22.08898400	29.09695000	9.69391200
H	21.77832200	28.81475700	10.70534200
H	23.02983400	29.65993200	9.72037700
C	20.99409200	29.90019800	9.04406800
O	21.06195400	30.97559900	8.52616500
C	18.62123200	28.09351100	13.50336300
H	18.02119100	28.58044200	12.72475800

C	17.72202200	27.24104700	14.43090800
H	17.38645100	26.36907700	13.85427100
H	18.34794200	26.84773900	15.24144700
C	16.48394500	27.93288300	15.02817700
H	16.77647800	28.78757000	15.65132800
C	15.44554800	28.38241400	13.99157600
H	15.12111100	27.54258700	13.36561000
H	14.55569900	28.79166100	14.48307800
H	15.83200800	29.15842700	13.31725200
C	19.42277400	29.11425300	14.29349100
O	20.53620300	28.87365200	14.75166000
N	18.79490600	30.32243400	14.48058400
H	17.81490400	30.38930200	14.23570200
C	19.27033900	31.29402300	15.46101100
H	20.32259300	31.07481700	15.62444800
C	19.09842400	32.70120400	14.90014500
H	19.59087400	32.76001000	13.92122000
H	18.03698000	32.89644700	14.71778000
C	19.67600600	33.80062600	15.78387300
O	19.42863100	35.01895700	15.38694700
O	20.35568800	33.50906700	16.77454700
C	18.43575600	31.11140500	16.74477400
O	17.22853500	31.42295200	16.71102000
N	19.00865900	30.53062900	17.80765400
C	20.44556100	30.20638300	17.96830700
H	21.05140700	31.09133600	17.75637800
H	20.73158800	29.40859000	17.27421500
C	20.54604000	29.75962200	19.43600100
H	20.73584500	30.62718100	20.07954100
H	21.35759100	29.04313500	19.59112500
C	19.15493200	29.17764100	19.73716500
H	18.92841200	29.13938000	20.80618400
H	19.07738800	28.15853200	19.34013600
C	18.20960000	30.11965400	18.98117700
H	17.28667600	29.64065300	18.64255900
N	16.25075400	32.03404600	21.28557100
H	16.44834900	32.94871200	21.69106100
C	14.99289500	32.09713600	20.52796600
H	14.15065600	32.51428900	21.10880600
C	15.18028000	32.91235600	19.24404500
H	15.56442800	33.91030500	19.50889600
H	15.94591500	32.42864300	18.62752200
C	13.87097400	33.08194900	18.46201500
H	13.45855900	32.09791200	18.19754900
H	13.12246400	33.55702500	19.10854700
C	14.00712700	33.93814400	17.19006500
H	13.01151300	34.28159600	16.87315700
H	14.59475400	34.83869300	17.42159300
C	14.65798900	33.18410400	16.03351500
H	15.64498200	32.77780100	16.27076100
H	14.02566500	32.35768300	15.69622600
N	14.83029100	34.10283500	14.84471500
H	15.54926600	34.83917500	15.00087900
H	15.19035200	33.62275700	13.98406400
H	13.94665900	34.56605200	14.61127500
C	25.02720900	34.04458300	20.54669300
H	24.57046100	33.37907200	19.80311500
C	23.99012500	34.95715300	21.22403700
H	23.23229900	34.31553400	21.68902600
H	24.47259700	35.51619200	22.03295800
C	23.30946600	35.97856100	20.28285200
H	22.52432700	36.50334300	20.83984400
H	24.04616400	36.72051900	19.95875000

C	22.74326800	35.34506300	19.01887100
O	23.46886400	35.16821500	18.01954800
N	21.45336200	34.96774100	19.03039800
H	20.88316700	35.09695800	19.85333200
H	21.04058600	34.51063400	18.20785600
C	26.16521500	34.80281200	19.87870900
O	26.75201200	34.51816100	18.88081000
C	26.39557100	33.20285900	15.35093900
C	26.61175000	34.62231600	14.82809400
H	20.52032000	34.84963000	8.26291800
H	22.14765800	33.73509600	9.77132100
O	25.78116300	35.51873400	15.05785600
C	24.92413500	32.90706100	15.70502900
C	20.50299000	35.05919200	9.32813200
C	22.91365800	34.11702300	14.79785400
C	21.40922700	34.42221500	10.17412700
C	21.36715100	34.70434100	11.54384800
C	19.61547600	36.21529800	11.22509400
C	19.58545600	35.96933400	9.85559500
C	22.27532600	34.09778100	12.54287900
C	23.25525400	33.14689900	12.25356200
C	24.09747000	32.71392300	13.28066300
C	23.96689300	33.22017300	14.57697200
N	20.47790800	35.59665800	12.04236500
N	22.11503400	34.54228800	13.81461900
H	26.76751400	32.47020800	14.62769200
H	24.84992100	31.84556700	15.97441200
H	24.63598300	33.48659800	16.58501100
H	22.72820000	34.50648700	15.79390500
H	18.95180400	36.93221900	11.69651100
H	18.87502700	36.48995600	9.22238100
H	23.38269000	32.76358400	11.24511400
H	24.87376000	31.98815400	13.05282400
N	27.73484600	34.88155100	14.12225800
H	27.76893700	35.80891900	13.70706000
C	28.73899100	33.94046000	13.62562100
H	28.81362800	33.10520000	14.32489500
C	28.46058400	33.42184900	12.20090400
H	29.05097900	32.50728800	12.04989400
H	27.40647100	33.12417300	12.12527000
C	28.83290800	34.38493900	11.06413300
H	29.90784300	34.59614700	11.10112800
H	28.64045500	33.91255900	10.09433000
S	28.05412100	36.05097900	11.05550200
C	26.34863300	35.63137700	10.55650200
H	25.80239800	36.57557100	10.48730400
H	26.34225000	35.14491900	9.57605100
H	25.85086100	34.99012100	11.29099800
Cu	20.74392700	35.99399900	14.05795200
O	19.55464900	37.61907400	14.07040500
N	21.89385100	37.26347000	15.23017600
C	19.99762100	38.61802300	14.72475000
C	19.19943300	39.82593100	14.83177100
C	18.05305900	39.95096300	14.13904200
C	17.15755700	41.14264700	14.16801700
H	17.05520700	41.56684600	13.16000000
H	19.53987800	40.62986800	15.47188700
H	17.52155700	41.92398800	14.84046400
H	16.14464000	40.85490400	14.48069300
H	17.75572400	39.12944200	13.48995300
C	21.29063500	38.46385400	15.34035500
N	22.09972300	39.31431600	16.06378800
C	23.20789500	38.61063000	16.40834300

C	23.07002100	37.33364900	15.87517700
H	23.77743600	36.52317700	15.92377300
H	24.02720000	39.02898800	16.97200000
C	21.93303600	40.74300000	16.34733800
H	22.84772600	41.09668200	16.82237800
H	21.79119200	41.29115600	15.41368700
H	21.09199800	40.90796900	17.02501900
C	23.14684900	38.80106000	11.62817900
C	23.07269400	40.20914800	11.70149600
C	23.85261000	40.92231100	12.60575700
C	24.72319100	40.22340800	13.46368000
C	24.78460400	38.80052700	13.37136600
C	24.00061900	38.08077500	12.46000800
C	25.63751300	40.60643900	14.50371600
C	26.19621300	39.44876900	14.98896000
N	25.69603500	38.35928400	14.30630200
H	22.54214100	38.27284300	10.89481100
H	22.41302900	40.74332300	11.02294300
H	23.80439600	42.00809400	12.63674900
H	24.06883600	36.99797900	12.40034600
H	25.87647700	41.61146400	14.82421400
H	26.92421400	39.30780700	15.77570000
H	25.85108800	37.38193400	14.56186900
O	23.55321800	32.20983200	9.09115300
H	22.76298200	31.80854600	8.67836500
H	24.17835100	32.35628000	8.36442600
O	16.37283000	33.50486500	12.67964600
H	16.81706800	34.34851000	12.92449300
H	16.17084800	33.54726500	11.73278500
O	17.14259000	35.61756000	14.26297500
H	17.30769900	36.56494900	14.12926500
H	17.99254300	35.31281100	14.72467600
O	25.42030300	37.24513700	17.91822800
H	26.32766200	36.91931600	18.01705800
H	24.88257200	36.42691900	17.88833200
H	22.21688800	28.16640900	9.12907200
H	27.00206100	33.07891800	16.25782100
H	25.50387800	33.41536600	21.31103700
H	16.14241000	31.39960600	22.07551300
H	14.71154400	31.06971000	20.26555400
H	19.34305700	27.43726100	13.00916100
H	29.70862600	34.45031900	13.65527300
H	17.92708800	30.98807900	19.58818700
H	16.00637200	27.21864300	15.71088700

^{2}TS1$_{ProS,B}$

C	22.74430000	28.28255900	11.37140400
H	22.09214400	28.40134300	12.24903800
H	23.26833700	29.22702400	11.17574500
C	21.84507200	27.91374900	10.22490200
O	21.38814600	28.60766800	9.36612200
C	18.53386200	28.46415100	11.96083300
H	17.49740900	28.77364500	11.78345400
C	18.62461500	26.94201700	12.18553300
H	18.28955900	26.44279200	11.26771200
H	19.68035200	26.68041500	12.31830000
C	17.81497900	26.41165600	13.38032800
H	18.15910400	26.90312400	14.30225300
C	16.29578300	26.57039200	13.24344900
H	15.92959400	26.08938000	12.32866900
H	15.77550200	26.10859000	14.08969900
H	15.98820400	27.62299200	13.20975700
C	19.14863400	29.25153700	13.11045500
O	20.29195400	29.04362200	13.52126900
N	18.35253600	30.22573900	13.65100900
H	17.38232800	30.28048800	13.36838400
C	18.74095600	31.02702900	14.81107500
H	19.77365500	30.75545900	15.02568300
C	18.65621900	32.52182200	14.46483200
H	19.28028200	32.70959500	13.59320200
H	17.62079900	32.76379800	14.18708200
C	19.03317000	33.47538200	15.59329200
O	19.56092500	34.61061900	15.29583100
O	18.74725100	33.16453400	16.77300500
C	17.78844500	30.61374000	15.94252300
O	16.58560100	30.95332000	15.87246200
N	18.24213900	29.79171700	16.89768900
C	19.64341700	29.34457900	17.09176500
H	20.30496400	30.20989000	17.19737200
H	19.97607900	28.75938100	16.22821800
C	19.57743700	28.49882400	18.37600700
H	19.77019900	29.12974000	19.25175700
H	20.31943500	27.69621700	18.37391200
C	18.12851500	27.98416800	18.40669300
H	17.80411100	27.67213500	19.40290200
H	18.01263400	27.12801600	17.73229600
C	17.32563600	29.17684000	17.87596400
H	16.38795100	28.90929800	17.38461000
N	19.48630600	34.65373800	21.44217400
H	19.52204100	35.66904000	21.34472100
C	18.08677700	34.23595000	21.66167800
H	17.59003600	34.83219600	22.44352600
C	17.26778500	34.31186200	20.36703000
H	17.37297100	35.31477300	19.92596500
H	17.70581700	33.60700200	19.64723300
C	15.77999200	34.01894700	20.61533600
H	15.67193200	33.02345700	21.06929800
H	15.41190500	34.72897600	21.36703400
C	14.84481500	34.10945000	19.39460700
H	13.81324700	34.10535400	19.76506800
H	14.97388300	35.07044100	18.87665700
C	14.94717100	32.96940200	18.37199400
H	15.18182600	32.01568200	18.85449400
H	14.00018500	32.84383500	17.84073200
N	15.97651400	33.20041600	17.31069300
H	16.93858500	33.30863300	17.65939100
H	16.05113800	32.37525300	16.66917500
H	15.77732600	34.06729700	16.74842100
C	24.13428800	31.85426900	18.75475200
H	23.38581300	31.62415300	17.98693000
C	23.53795600	32.62408000	19.93952300
H	22.71986400	32.02622500	20.35894400
H	24.29493500	32.71925800	20.72632300
C	23.02466600	34.04131500	19.59943200
H	22.57236600	34.47685500	20.49723000
H	23.86823300	34.67530900	19.30771800
C	22.03482700	34.04195600	18.44458400
O	22.45405600	33.98468200	17.26683400
N	20.73292400	34.06450200	18.75582900
H	20.39447800	34.14056900	19.72344400
H	20.04426900	33.92398100	18.01239700
C	25.28136100	32.57577000	18.06395300
O	25.72900800	32.36383600	16.98064800
C	26.28856300	33.92740000	13.53650800
C	25.71085800	34.98743400	14.47176500
H	18.00876300	33.74084900	8.93181800

H	20.18066300	32.72269700	9.59095500
O	24.58208900	35.46747200	14.24715700
C	25.49281800	32.58503200	13.52705300
C	18.49781800	34.05446400	9.84951400
C	23.04797100	33.17864900	13.70879600
C	19.71556900	33.48243300	10.21602400
C	20.32704300	33.89677400	11.40613900
C	18.58192500	35.39417700	11.83098400
C	17.91689900	35.02694600	10.66439100
C	21.63073600	33.35977500	11.88265700
C	22.58552200	32.78191400	11.03982300
C	23.84346600	32.48661500	11.57509300
C	24.11461900	32.71449300	12.92749900
N	19.74643800	34.83790300	12.19152500
N	21.86771300	33.52830200	13.20050000
H	26.28171500	34.35541800	12.52731900
H	26.08126300	31.86434100	12.94837000
H	25.43026900	32.20242700	14.55100800
H	23.16388000	33.31163500	14.77965700
H	18.20384900	36.16409900	12.49595300
H	16.97452000	35.49740700	10.40481400
H	22.37526900	32.58725800	9.99220500
H	24.62276600	32.09967500	10.92207900
N	26.45029000	35.40364500	15.51891800
H	25.98666000	36.07647200	16.12743100
C	27.75653200	34.90425900	15.96753500
H	28.46837900	34.94779800	15.13566400
C	28.32656900	35.73315700	17.12533500
H	29.33186400	35.33844700	17.32103600
H	28.46554500	36.77360600	16.80639600
C	27.53498900	35.67529200	18.44266100
H	27.16043400	34.66219600	18.62625000
H	28.18173100	35.93986400	19.28554500
S	26.04509400	36.75928900	18.53519100
C	26.83123200	38.38741300	18.80974400
H	26.02613800	39.12374100	18.87371100
H	27.38982400	38.39145500	19.75002500
H	27.49344200	38.66261000	17.98428100
Cu	20.77845300	35.44694600	13.89268800
O	19.56928800	37.67850000	14.05157500
N	21.79164400	36.79023800	12.69453000
C	20.27223400	38.57012500	13.45072800
C	20.19836700	39.94954100	13.65230000
C	19.28372500	40.58215300	14.54861500
C	18.97291000	42.04256600	14.28580800
H	18.38953300	42.10556700	13.35811600
H	20.93620800	40.58527800	13.17716300
H	19.88460800	42.63260000	14.14091800
H	18.37825600	42.49578800	15.08049700
H	18.40971400	39.98901600	14.81396000
C	21.31456000	38.02757400	12.53193200
N	21.97035600	38.63189100	11.49365600
C	22.90450100	37.73546900	11.01628100
C	22.78679800	36.60121800	11.77325800
H	23.36411200	35.69207500	11.73688300
H	23.55403300	37.98326400	10.19023500
C	21.72123200	39.93591700	10.87773000
H	21.99791400	39.87661700	9.82344900
H	20.66289300	40.18460100	10.95701400
H	22.31660600	40.71831700	11.35789100
C	17.08132900	41.60213400	19.19913000
C	17.73485400	42.70393300	18.62085900
C	18.76027800	42.53161500	17.69058400
C	19.13672000	41.23001000	17.33963000
C	18.48572200	40.14123000	17.95908600
C	17.45124500	40.29647700	18.87888000
C	20.10040300	40.66693600	16.38174200
C	20.08135300	39.26532900	16.63847300
N	19.10547200	38.96701100	17.49937900
H	16.28405400	41.77084600	19.91635200
H	17.43766900	43.70781200	18.90813600
H	19.25965600	43.39389400	17.25876000
H	16.96270400	39.44234400	19.33825500
H	21.00585100	41.18343500	16.08684800
H	20.64227200	38.47892700	16.15375600
H	18.71495200	38.01896800	17.52550700
O	21.39716100	31.35683300	8.42477700
H	21.40447800	30.43228000	8.73751900
H	21.41858100	31.29153000	7.45822400
O	15.76673500	35.51216400	15.83733100
H	16.59617700	36.04460100	15.96887100
H	15.03258200	36.14182800	15.79222600
O	18.17465700	36.70810500	16.23362500
H	18.47534500	37.22333700	15.44550600
H	18.74014600	35.89837800	16.14809200
O	22.05370700	35.88643100	15.40230900
H	22.98001800	35.89457600	15.05264800
H	22.04901700	35.17072400	16.09705400
H	23.45392300	27.47668100	11.57416700
H	27.32729400	33.69939100	13.78419700
H	24.55633000	30.90057400	19.10261100
H	20.03403200	34.44267900	22.27599300
H	18.10259400	33.20021300	22.01996600
H	19.09882400	28.72315800	11.05677200
H	27.66459800	33.85731100	16.27942300
H	17.09598100	29.89417100	18.67727900
H	18.05119300	25.34729000	13.50321600

^{2}TS1$_{ProR,B}$

C	20.13194100	27.84456200	10.88377000
H	19.37855200	28.06453300	11.65268600
H	21.07500600	27.52883800	11.34767100
C	20.31564900	29.08426100	10.05820600
O	21.26904700	29.79758500	9.94448200
C	15.61703600	28.69986500	12.80124300
H	14.69807900	29.19154900	13.14103200
C	15.58729300	27.19194000	13.11596000
H	14.76543900	26.73907300	12.54691600
H	16.51652400	26.75175300	12.73758900
C	15.42900200	26.84396800	14.60526800
H	16.26539700	27.27610800	15.17338700
C	14.09801300	27.28561300	15.22637100
H	13.24660700	26.87016700	14.67383400
H	14.01689700	26.94351200	16.26389200
H	13.98618600	28.37699200	15.23549000
C	16.85041000	29.38022600	13.38083900
O	17.98992100	28.96161400	13.16883500
N	16.61718200	30.49523200	14.13785800
H	15.66380500	30.75136000	14.35716300
C	17.68414200	31.25943800	14.78141800
H	18.61559400	30.82405100	14.42710900
C	17.61673300	32.73173400	14.37623400
H	17.61787900	32.80270500	13.28137100
H	16.67454600	33.17488400	14.71304500
C	18.80179800	33.54955900	14.88943900

O	18.77041100	34.81660800	14.58403900
O	19.73805500	33.00222100	15.48329700
C	17.52775400	31.10191700	16.30495000
O	16.66470300	31.76993800	16.90181000
N	18.27321200	30.16676800	16.92049000
C	19.41765100	29.42064600	16.34424400
H	20.13452400	30.11698900	15.90015500
H	19.07505300	28.73320600	15.56397300
C	20.00234600	28.67576700	17.55629100
H	20.75612200	29.30360600	18.04597300
H	20.48080600	27.73568500	17.26869500
C	18.79354300	28.48458200	18.48690200
H	19.07577200	28.28887700	19.52538000
H	18.17656200	27.64561900	18.14419400
C	18.02660400	29.80273200	18.32701700
H	16.95116600	29.72169600	18.50437300
N	19.10239800	32.52087900	20.85842700
H	19.60559100	33.33733900	21.20846100
C	17.65235500	32.80926000	20.90977400
H	17.34659500	33.21528500	21.88749200
C	17.24882400	33.77504900	19.79303700
H	17.93267300	34.63768400	19.79590600
H	17.37277200	33.26656800	18.83024700
C	15.81013400	34.28669100	19.95651600
H	15.11782400	33.43900900	20.05637000
H	15.73192200	34.85254900	20.89275600
C	15.34484200	35.19334600	18.80079000
H	14.50479900	35.81682000	19.13747600
H	16.15939900	35.88013200	18.52849800
C	14.90831800	34.39908200	17.57029000
H	15.66212200	33.69016100	17.21705600
H	13.99184600	33.83667900	17.77025200
N	14.60182600	35.32951600	16.42131700
H	15.45095800	35.81173800	16.05128900
H	14.24909400	34.83515500	15.56439900
H	13.91840300	36.04230500	16.69387100
C	24.98002300	31.75099900	19.33555200
H	24.78845200	31.72422400	18.25723000
C	23.68817400	31.63334200	20.15718600
H	23.20710300	30.67830300	19.91387300
H	23.94638100	31.59676800	21.22167300
C	22.67969100	32.78762600	19.95533000
H	21.84339700	32.64395500	20.64666900
H	23.16223400	33.74087400	20.20554800
C	22.18310600	32.91007600	18.51989100
O	22.95018300	33.29071000	17.61850800
N	20.89656300	32.57865600	18.28398000
H	20.23784500	32.40342200	19.04835200
H	20.54026600	32.67730000	17.33280400
C	25.75629500	33.02006100	19.65283500
O	26.44468900	33.66589300	18.92433200
C	27.32163700	35.28750500	15.05424100
C	27.13975500	36.67377900	14.44600900
H	20.44160100	32.50376400	9.20573300
H	22.44397200	32.42478700	10.65644700
O	26.00347100	37.15456500	14.27507100
C	26.04615100	34.70096500	15.68415400
C	20.48349500	33.12040100	10.09799100
C	23.67412100	34.98471200	14.87089500
C	21.61014100	33.07085500	10.91519600
C	21.64190100	33.85088000	12.07721800
C	19.50952400	34.68677800	11.62068500
C	19.41755700	33.95379800	10.44204700
C	22.80256300	33.92308500	12.99288800
C	23.99450500	33.22095400	12.80113800
C	25.05722700	33.45879200	13.67500400
C	24.92958200	34.37899400	14.71777700
N	20.58656500	34.62686800	12.41506100
N	22.65555700	34.76744300	14.03992900
H	27.71736800	34.62089300	14.27442200
H	26.33445200	33.78649300	16.21679800
H	25.66471000	35.38515600	16.44622700
H	23.49464400	35.66419100	15.69619900
H	18.70333400	35.32380600	11.96667700
H	18.53092400	34.02836400	9.82142900
H	24.09746000	32.49530200	11.99926400
H	25.98942700	32.91756000	13.53177300
N	28.25142700	37.34575000	14.08402900
H	28.10642100	38.29159500	13.73235300
C	29.64404900	36.94964900	14.31456800
H	29.77146500	35.90004200	14.02958300
C	30.63032800	37.80253200	13.50573800
H	31.62273500	37.35758900	13.65387200
H	30.40965800	37.70861900	12.43544300
C	30.72146100	39.28350000	13.90907700
H	30.74584900	39.38541100	14.99984300
H	31.65119600	39.71983600	13.53005100
S	29.34133100	40.37545700	13.35311200
C	29.76317700	40.57819200	11.58515300
H	29.00370300	41.23371700	11.15165700
H	30.74089500	41.05653200	11.47986900
H	29.75523700	39.62602600	11.04759200
Cu	20.69440300	35.56092700	14.19779900
O	20.64933700	37.62061700	13.54480600
N	20.91338300	36.50199200	15.91702900
C	21.07586300	38.42642400	14.45136600
C	21.48085000	39.74190700	14.24940200
C	21.40011900	40.43156400	12.99177400
C	21.16768600	41.93361200	13.07735400
H	21.14089300	42.41342400	12.09454600
H	21.90418000	40.28933600	15.08362000
H	21.93404200	42.43072700	13.68368100
H	20.20108000	42.11533200	13.56281500
H	20.76455900	39.92428500	12.26730600
C	21.10618600	37.82102100	15.81803200
N	21.25390200	38.33875100	17.07512200
C	21.15445200	37.29225200	17.97082500
C	20.93903600	36.15626200	17.23857100
H	20.81741800	35.13537400	17.56167700
H	21.28128100	37.44668600	19.03099400
C	21.45418800	39.72781600	17.48052000
H	21.27958400	39.79892500	18.55497000
H	22.47698800	40.05193000	17.26882600
H	20.74814100	40.37730800	16.95957300
C	23.36550400	36.46912700	10.20246900
C	22.46135200	37.32932700	9.55574000
C	22.24737000	38.63207900	10.00952800
C	22.96731800	39.07228100	11.12153800
C	23.87162500	38.19362200	11.74565400
C	24.08842800	36.88725200	11.31946800
C	23.00451700	40.33826000	11.87405400
C	24.09404300	40.16280900	12.79205600
N	24.51127900	38.90615000	12.77705800
H	23.51315700	35.46494400	9.81735200
H	21.92871000	36.97661700	8.67737200
H	21.55214300	39.28906600	9.49447600

H	24.79424800	36.23872900	11.82655300
H	22.88196400	41.29014300	11.36862600
H	24.48651900	40.87112700	13.51056000
H	25.19939800	38.45720700	13.41047400
O	23.93193900	30.81344400	10.49472400
H	23.13284800	30.25755800	10.43986400
H	24.34233800	30.72889300	9.62048400
O	14.44906800	34.42355700	13.86862600
H	15.17608400	35.06931400	13.72042400
H	13.80419700	34.54813500	13.15569400
O	16.50642700	36.13798100	14.54409200
H	16.73885100	37.03643400	14.26072500
H	17.37944200	35.62530800	14.54575400
O	24.14472800	35.74640500	18.38394300
H	25.02524900	35.54566600	18.73732200
H	23.81405300	34.87616600	18.07764700
H	19.73187300	27.04595600	10.25056700
H	28.10146200	35.33189800	15.82395500
H	25.65302800	30.92019700	19.59017700
H	19.31700000	31.77224400	21.51738600
H	17.11876100	31.86114700	20.77971600
H	15.66443600	28.83528200	11.71303200
H	29.88945900	37.02796000	15.38403700
H	18.42219200	30.58046500	18.99309200
H	15.52607600	25.75615900	14.71088500

^{2}Int1$_{ProS,B}$

C	20.02348100	27.96307000	10.59850400
H	19.34878700	28.10659600	11.45439500
H	21.01854300	27.65405500	10.94302800
C	20.08018400	29.26353900	9.85361200
O	20.98100700	30.04136400	9.73003700
C	15.75603400	28.66694100	12.75934800
H	14.83254000	29.15107500	13.09756700
C	15.73911100	27.15930200	13.07975500
H	14.92116500	26.69735300	12.51230300
H	16.67199500	26.72522600	12.70302300
C	15.58358500	26.81522200	14.57035800
H	16.41162700	27.26307500	15.13854100
C	14.24450100	27.23862600	15.18667700
H	13.40107000	26.80404100	14.63666800
H	14.16831900	26.90383400	16.22695700
H	14.11364800	28.32796000	15.18664800
C	16.98308100	29.35815500	13.33893000
O	18.12855400	28.97457400	13.09321100
N	16.73875600	30.43862300	14.13977500
H	15.78401300	30.67176100	14.37797600
C	17.80079800	31.19399700	14.80133100
H	18.73406800	30.75732300	14.45434200
C	17.75082600	32.67190900	14.41476400
H	17.81537700	32.76266100	13.32313200
H	16.78933900	33.10738500	14.70358100
C	18.90189100	33.47875800	15.01988100
O	18.85287300	34.76130500	14.80231200
O	19.81850900	32.90234300	15.61677700
C	17.62396800	31.02431400	16.32077700
O	16.74058100	31.67435600	16.90849200
N	18.37742900	30.10016000	16.94306100
C	19.55046900	29.38892600	16.38214100
H	20.24578800	30.10670200	15.93867400
H	19.23661400	28.68318900	15.60536100
C	20.14990400	28.67317700	17.60472200
H	20.88070700	29.32871700	18.09315900
H	20.65960500	27.74597200	17.32929600
C	18.94159200	28.45308200	18.52990500
H	19.22300500	28.27639300	19.57200100
H	18.35347200	27.59188300	18.19153400
C	18.13502200	29.74469000	18.35166000
H	17.06183900	29.63250100	18.52606000
N	19.11183300	32.53598300	20.90562400
H	19.57305800	33.36058100	21.29245500
C	17.65220700	32.77724500	20.90035100
H	17.29504800	33.16641400	21.86751900
C	17.26110200	33.73715900	19.77400400
H	17.92451100	34.61518400	19.79984100
H	17.42569600	33.23483000	18.81404800
C	15.80653400	34.21442600	19.89583700
H	15.13173600	33.35024400	19.97151900
H	15.68745600	34.77367400	20.83185500
C	15.35473200	35.11586400	18.73094800
H	14.49444800	35.72235300	19.04718400
H	16.16300900	35.81863700	18.48181300
C	14.96528500	34.31856600	17.48671100
H	15.74048800	33.62357700	17.15229500
H	14.05419500	33.73896800	17.66051200
N	14.67258700	35.24968200	16.33504700
H	15.52413400	35.74624700	15.98557700
H	14.34693600	34.75717800	15.46718800
H	13.97297400	35.95113300	16.59545200
C	25.04167700	31.97274900	19.49472000
H	24.85024000	31.89958300	18.41860500
C	23.75269600	31.86001500	20.32138000
H	23.28638400	30.89095600	20.10672700
H	24.01214700	31.85876200	21.38622200
C	22.72634300	32.99185300	20.08657000
H	21.88514700	32.84767900	20.77224000
H	23.19148800	33.95652400	20.32617600
C	22.24418800	33.08000800	18.64309900
O	23.01565600	33.46728000	17.74718100
N	20.97202900	32.71265900	18.39521100
H	20.31471700	32.50067000	19.15178200
H	20.62656600	32.77734600	17.43549200
C	25.79379500	33.26810000	19.75897900
O	26.48223800	33.88741400	19.00740700
C	27.28395900	35.04213500	15.01984900
C	27.12900000	36.42491500	14.39896100
H	20.16783400	32.81783600	9.22649100
H	22.19893600	32.60658000	10.62079800
O	25.99639400	36.89423700	14.16695000
C	25.99871400	34.48552800	15.65912600
C	20.26173800	33.40927600	10.13187500
C	23.62719400	34.90413800	14.91723000
C	21.40330900	33.28267700	10.91886500
C	21.50073300	34.02503900	12.10264700
C	19.40803600	34.98501200	11.71539900
C	19.24683700	34.28425900	10.52470700
C	22.67759900	33.99127000	13.00215400
C	23.82852700	33.24008500	12.75163200
C	24.91487800	33.37721900	13.61839800
C	24.84886900	34.24748500	14.70796500
N	20.49863700	34.85104200	12.48109300
N	22.58677400	34.78228600	14.09530400
H	27.65983500	34.36412000	14.23955500
H	26.26409200	33.54312700	16.15383600
H	25.66398400	35.15943100	16.45166200

H	23.49753600	35.55339100	15.77610900
H	18.64584100	35.65837900	12.09271000
H	18.34870800	34.41631300	9.93079300
H	23.88501000	32.55597800	11.90967500
H	25.81744700	32.80053400	13.42941600
N	28.24867600	37.10423200	14.08815400
H	28.11461100	38.04432100	13.71633700
C	29.63347000	36.71497500	14.37814400
H	29.77863400	35.66715700	14.09576200
C	30.64863200	37.57612900	13.61560600
H	31.63652400	37.14002200	13.81174800
H	30.47989500	37.47863200	12.53621000
C	30.70573300	39.05856600	14.01972900
H	30.66956700	39.16389000	15.10979700
H	31.65066500	39.50236400	13.69053400
S	29.34837400	40.13508100	13.38285900
C	29.87806500	40.35043500	11.64546400
H	29.13558000	40.99221900	11.16453800
H	30.85088400	40.84804900	11.60424900
H	29.92393300	39.39936900	11.10785000
Cu	20.66504100	35.73430500	14.29559600
O	20.58236000	37.69093500	13.62634900
N	20.88089600	36.66380200	16.02368500
C	20.97767400	38.55645900	14.52790600
C	21.39852900	39.83093600	14.25804800
C	21.41803700	40.41754900	12.86115700
C	21.11335000	41.92236500	12.87904300
H	21.09803600	42.34615700	11.86839600
H	21.69879300	40.48678100	15.06760800
H	21.85841800	42.47742400	13.46399500
H	20.13521500	42.10273000	13.33665400
H	20.66147700	39.90356000	12.25964300
C	20.98464200	37.99080700	15.90273100
N	21.05172100	38.54644100	17.14727500
C	20.99283900	37.51380000	18.06745900
C	20.88213100	36.35037900	17.35674500
H	20.81353700	35.33108400	17.70185000
H	21.06169700	37.70318200	19.12759400
C	21.13694400	39.95821800	17.50532700
H	20.86225100	40.06674500	18.55572700
H	22.15424600	40.33637500	17.36641700
H	20.44545900	40.53930700	16.89189500
C	23.43931500	36.60188700	9.81783300
C	22.46834400	37.49028200	9.33916100
C	22.16541300	38.67798900	10.02028700
C	22.85339800	38.95861800	11.19295500
C	23.82108900	38.05509700	11.64459700
C	24.14357600	36.87234800	10.99602500
C	22.80366300	40.13050800	12.15183200
C	23.84592900	39.71865600	13.15495500
N	24.38922000	38.58274800	12.83858300
H	23.65335100	35.69256100	9.26510400
H	21.94273600	37.25773000	8.41791100
H	21.41180100	39.35701400	9.63245000
H	24.89554300	36.19608500	11.38807600
H	23.15316800	41.04470600	11.64503000
H	24.17366800	40.27266600	14.02478500
H	25.12093000	38.06763100	13.38940300
O	23.65795500	30.97313600	10.31051900
H	22.83916300	30.44907700	10.23074200
H	24.05696600	30.92682300	9.42823400
O	14.60246800	34.38117800	13.76485900
H	15.33115900	35.03168700	13.65093100
H	13.98015400	34.51643000	13.03421000
O	16.63197500	36.08559500	14.54242800
H	16.91271900	36.98536700	14.31152700
H	17.49274900	35.54918100	14.62414400
O	24.22823200	35.95583600	18.29357400
H	25.09899300	35.76876700	18.67740400
H	23.86133500	35.06552400	18.10944600
H	19.58443400	27.19420100	9.95505900
H	28.06801200	35.07256200	15.78526800
H	25.73168300	31.16635100	19.78069000
H	19.32309200	31.78091100	21.55817700
H	17.15529300	31.81339800	20.74391400
H	15.80344400	28.79872300	11.67098800
H	29.82899100	36.79215900	15.45751100
H	18.50394800	30.54223500	19.01010100
H	15.69747100	25.72954100	14.68096500

^{2}Int1$_{ProR,B}$

C	22.69491100	28.27922900	11.34712600
H	22.02718100	28.39933500	12.21289300
H	23.22428200	29.22309700	11.16350400
C	21.81587900	27.91663200	10.18311600
O	21.36368200	28.61877400	9.32832400
C	18.49033700	28.61216800	11.88459400
H	17.48571700	28.99561000	11.67271800
C	18.46429100	27.08545700	12.09833700
H	18.12131100	26.61871800	11.16646100
H	19.49404200	26.74688200	12.25934600
C	17.58370600	26.60613100	13.26417300
H	17.94766000	27.04780600	14.20358200
C	16.08750700	26.90033500	13.09916200
H	15.70232600	26.47836500	12.16322000
H	15.50921700	26.46353100	13.92060500
H	15.87404800	27.97640800	13.08997000
C	19.11711700	29.34417600	13.06333400
O	20.24421100	29.07786400	13.48554300
N	18.35178000	30.33452700	13.61860900
H	17.38636200	30.42678400	13.32956800
C	18.75351000	31.08924800	14.80530700
H	19.79049400	30.81468600	14.99363400
C	18.65477100	32.59535700	14.51848700
H	19.24797500	32.81815400	13.63448600
H	17.60983000	32.84499500	14.28646400
C	19.07108600	33.50661300	15.66749500
O	19.61267600	34.64034000	15.39291400
O	18.80519800	33.16185500	16.84310700
C	17.82115300	30.62809800	15.93539800
O	16.61653700	30.96794700	15.90078500
N	18.29147900	29.77001300	16.84978400
C	19.69712100	29.32121100	17.00778600
H	20.35735900	30.18504900	17.13185500
H	20.01744400	28.76644000	16.12017300
C	19.65271400	28.43438900	18.26482700
H	19.85477000	29.03763900	19.15784000
H	20.39803800	27.63577100	18.22655500
C	18.20662300	27.91209300	18.29889700
H	17.89732500	27.56645300	19.28883000
H	18.08515300	27.07752700	17.59887600
C	17.39098400	29.11742900	17.81830500
H	16.44832300	28.86112500	17.33044100
N	19.60058400	34.43210100	21.58026200
H	19.63995400	35.45128100	21.54517000
C	18.20237200	34.00726200	21.79489500

H	17.71625400	34.56430300	22.61163500
C	17.36987500	34.15003800	20.51469200
H	17.47755600	35.17198900	20.12051500
H	17.79588700	33.47763500	19.75771900
C	15.88270500	33.85584700	20.76346300
H	15.77227200	32.84391700	21.17894800
H	15.52456000	34.53783800	21.54542100
C	14.93964900	33.99870200	19.55382800
H	13.91070000	33.98757000	19.93138500
H	15.07171300	34.97678600	19.06976400
C	15.02717300	32.89628300	18.48943200
H	15.26267400	31.92439600	18.93374600
H	14.07431700	32.79386700	17.96367600
N	16.04641400	33.16119000	17.42624700
H	17.01425800	33.24967000	17.76507900
H	16.10716700	32.36088800	16.75185300
H	15.85010900	34.05020400	16.90338800
C	24.18928000	31.77810900	18.78418900
H	23.44987300	31.59096700	17.99600400
C	23.59009600	32.52467600	19.98187100
H	22.75873000	31.92849700	20.37698400
H	24.33985400	32.58894700	20.77874300
C	23.10052600	33.95658700	19.67044900
H	22.65584800	34.38142500	20.57732700
H	23.95343800	34.58298100	19.38975900
C	22.10774600	33.99493100	18.51781200
O	22.52248800	33.98379900	17.33851700
N	20.80617900	33.99934400	18.83637500
H	20.47640800	34.02356500	19.80925600
H	20.11418600	33.87524600	18.09273100
C	25.36575500	32.49432300	18.13828500
O	25.83155000	32.30315800	17.05900200
C	26.30150300	33.88647300	13.62980400
C	25.74407200	34.95875500	14.56326100
H	18.03926600	33.81321900	8.99263700
H	20.19124000	32.76565100	9.66866500
O	24.61984400	35.45082700	14.34667600
C	25.48134200	32.55923600	13.62343800
C	18.52660800	34.12184700	9.91297900
C	23.04190800	33.18366800	13.79850800
C	19.73287800	33.53260900	10.28949500
C	20.34227200	33.94112600	11.48283000
C	18.61911900	35.46748900	11.89042700
C	17.95610300	35.10649800	10.72047900
C	21.63548000	33.38622200	11.96762900
C	22.58717800	32.79995900	11.12700800
C	23.83870000	32.48786500	11.66637100
C	24.10664800	32.70943100	13.02037300
N	19.77121600	34.89240200	12.26194700
N	21.86679900	33.54721900	13.28710500
H	26.30209300	34.31251400	12.61969100
H	26.05814500	31.82619100	13.04844600
H	25.41147500	32.18095300	14.64851800
H	23.15539000	33.31284700	14.87028800
H	18.25639800	36.25021700	12.54947900
H	17.02406900	35.59235900	10.45163700
H	22.37977700	32.61270500	10.07759500
H	24.61635500	32.09355800	11.01581400
N	26.49905300	35.37266000	15.60111700
H	26.05003300	36.05598300	16.20838200
C	27.80820100	34.86693300	16.03326200
H	28.51347800	34.91745800	15.19596700
C	28.38926800	35.68306700	17.19459500
H	29.39375200	35.28180700	17.38081400
H	28.53157100	36.72561100	16.88395500
C	27.60462600	35.61774500	18.51589800
H	27.22015700	34.60664300	18.68953600
H	28.25880400	35.86492800	19.35834000
S	26.12801400	36.71783500	18.63001800
C	26.93594100	38.33235700	18.92099300
H	26.14077200	39.07824300	18.99756400
H	27.49913800	38.31799800	19.85845400
H	27.59766700	38.60921900	18.09570800
Cu	20.80019700	35.51096800	13.97874100
O	19.58069600	37.68015700	14.08498900
N	21.84048200	36.80331200	12.77723700
C	20.26507800	38.57919500	13.42457100
C	20.15881200	39.94212400	13.55230600
C	19.18509300	40.63074800	14.44197800
C	18.80556200	42.02092000	13.91578800
H	18.38200900	41.91813300	12.91157200
H	20.87457500	40.57991800	13.04707600
H	19.68390900	42.67359600	13.84229700
H	18.05899900	42.51146800	14.54524300
H	18.27990000	40.02489000	14.55902000
C	21.32690400	38.01430700	12.54443100
N	21.96689400	38.57487200	11.47343300
C	22.93291300	37.68048600	11.04997200
C	22.84540800	36.58920000	11.86771600
H	23.44895200	35.69687700	11.88823400
H	23.58076400	37.90372700	10.21577800
C	21.67718100	39.83047100	10.78321800
H	21.94859300	39.71660200	9.73178800
H	20.61249400	40.05208300	10.85978200
H	22.25109300	40.65875700	11.21021000
C	16.92395900	41.70559700	18.99693300
C	17.38154200	42.79300300	18.24020900
C	18.33327200	42.62691900	17.22713100
C	18.82694000	41.34814700	16.97908300
C	18.36950400	40.28287400	17.77124600
C	17.42000700	40.41838800	18.77636900
C	19.79726000	40.78834900	15.96912500
C	19.94321300	39.39168000	16.42355400
N	19.08318100	39.12347300	17.37288500
H	16.18404100	41.86709300	19.77444900
H	16.99319900	43.78530800	18.44737000
H	18.68036100	43.48388200	16.65889900
H	17.08861800	39.57333500	19.37180600
H	20.73464900	41.33724200	15.85369300
H	20.52144300	38.60118100	15.96461000
H	18.81537400	38.14371800	17.54777300
O	21.40221600	31.39992500	8.48348600
H	21.39805100	30.46536200	8.76490700
H	21.43634700	31.36650400	7.51570500
O	15.87387000	35.55237000	16.08347000
H	16.70740800	36.08581800	16.16857600
H	15.15345100	36.17130700	15.89878400
O	18.30268400	36.76126500	16.33788400
H	18.56750300	37.22397300	15.50038800
H	18.84925100	35.93630100	16.26529300
O	22.09224500	35.92261300	15.49869700
H	23.01492500	35.92332500	15.14159800
H	22.08702500	35.19539200	16.17877800
H	23.39883400	27.47149000	11.56125800
H	27.33606900	33.64015000	13.87743500
H	24.58261700	30.80386600	19.10790900

H	20.16036500	34.16756200	22.39046300
H	18.21905700	32.95476300	22.10040300
H	19.10514900	28.83613100	11.00441700
H	27.71751500	33.81729200	16.33590800
H	17.16745400	29.80635000	18.64590500
H	17.72336300	25.52302900	13.36966600

Model C structures:

$^{2}Re_{proS,C}$

6	40.959654000	38.964864000	27.259661000
1	40.951149000	40.060654000	27.244493000
6	39.742969000	38.411903000	28.005047000
1	38.818357000	38.699433000	27.489340000
1	39.775039000	37.315025000	28.001272000
6	39.682892000	38.920151000	29.464538000
1	40.628613000	38.678872000	29.966344000
1	39.582762000	40.011296000	29.470953000
6	38.521863000	38.352449000	30.270996000
8	37.377810000	38.836755000	30.240950000
7	38.801043000	37.266234000	31.024503000
1	39.743889000	36.918307000	31.122667000
1	38.096523000	36.897611000	31.653832000
6	42.800581000	42.599487000	26.097860000
1	43.180539000	42.959970000	25.137200000
6	41.268025000	42.841079000	26.174000000
1	40.781729000	42.060344000	25.572447000
1	40.930644000	42.702210000	27.207927000
6	40.843164000	44.200982000	25.656204000
6	40.786774000	44.436363000	24.274222000
1	41.012824000	43.626900000	23.582475000
6	40.473717000	45.700160000	23.773455000
1	40.458537000	45.867362000	22.699757000
6	40.193853000	46.750296000	24.651579000
1	39.969630000	47.740588000	24.264659000
6	40.222030000	46.522511000	26.028457000
1	40.017949000	47.337199000	26.718624000
6	40.546761000	45.258184000	26.526325000
1	40.611250000	45.100652000	27.599351000
6	43.516256000	43.346300000	27.213897000
8	43.590997000	44.568479000	27.248910000
7	44.003363000	42.540805000	28.209416000
1	43.917778000	41.539491000	28.111758000
6	44.564999000	43.068349000	29.438437000
1	44.747255000	44.129878000	29.261085000
6	43.658017000	42.843020000	30.658980000
1	43.504524000	41.763621000	30.793455000
1	44.224583000	43.169427000	31.544923000
6	42.307738000	43.536343000	30.666609000
6	41.168626000	42.841305000	31.098589000
1	41.258971000	41.790934000	31.370851000
6	39.932088000	43.479874000	31.221113000
1	39.070076000	42.921352000	31.580139000
6	39.811782000	44.834155000	30.904145000
1	38.857874000	45.344275000	31.018486000
6	40.934778000	45.535531000	30.456132000
1	40.856484000	46.590936000	30.209105000
6	42.168408000	44.893256000	30.337099000
1	43.023744000	45.456074000	29.977704000
6	34.276007000	46.627979000	24.020020000
1	33.823822000	46.091764000	23.179666000
6	35.698399000	47.116696000	23.625494000
1	35.534154000	47.790447000	22.772611000
6	36.340063000	47.939341000	24.752953000
1	37.287562000	48.375345000	24.420944000
1	35.690775000	48.764738000	25.070973000
1	36.558910000	47.319484000	25.631733000
6	36.636519000	45.991863000	23.148356000
1	37.605674000	46.449284000	22.915906000
1	36.824961000	45.294984000	23.974762000
6	36.146301000	45.214552000	21.921807000
1	35.247644000	44.626609000	22.141677000
1	36.915532000	44.515752000	21.575600000
1	35.912358000	45.886340000	21.086990000
6	34.242948000	45.689733000	25.212136000
8	34.606609000	44.504118000	25.166685000
7	33.797204000	46.208774000	26.387386000
1	33.488206000	47.170172000	26.421594000
6	33.790579000	45.423764000	27.608110000
1	34.819399000	45.219039000	27.933862000
6	33.030282000	46.173530000	28.719964000
1	33.424541000	47.196890000	28.764725000
1	31.971507000	46.253969000	28.438868000
6	33.120218000	45.524014000	30.107529000
1	32.439340000	46.035327000	30.795451000
1	32.809824000	44.476609000	30.061175000
16	34.793394000	45.499077000	30.871202000
6	34.943985000	47.230527000	31.443116000
1	35.905467000	47.311483000	31.956453000
1	34.936970000	47.935937000	30.608166000
1	34.144955000	47.478919000	32.147295000
6	33.132804000	44.045600000	27.365359000
8	33.323068000	43.068525000	28.020187000
7	31.931584000	42.091601000	24.232651000
1	32.619757000	42.645704000	24.762164000
6	32.652992000	41.169005000	23.400002000
1	32.100331000	41.070247000	22.449335000
6	34.112867000	41.525898000	23.068728000
1	34.578635000	40.660767000	22.589410000
1	34.122607000	42.360964000	22.356082000
8	34.868531000	41.840154000	24.230003000
1	34.809865000	42.798321000	24.408560000
6	32.541948000	39.725893000	24.012507000
8	33.362875000	38.853833000	23.749015000
7	31.440388000	39.551146000	24.781696000
1	30.851691000	40.371885000	24.890988000
6	31.007572000	38.266151000	25.304513000
1	31.826861000	37.563229000	25.137371000
6	30.645966000	38.340667000	26.793531000
1	31.532754000	38.621068000	27.372118000
1	29.895523000	39.125725000	26.949816000
6	30.078864000	37.016072000	27.332067000
1	29.706842000	37.173089000	28.352505000
1	29.219179000	36.696817000	26.730969000
6	31.129043000	35.912684000	27.414308000
8	32.227687000	36.092154000	27.951689000
7	30.788231000	34.718828000	26.872103000
1	29.881188000	34.548769000	26.462721000
1	31.420809000	33.934931000	26.969437000
6	33.530717000	34.568349000	22.570844000
6	33.689998000	35.191004000	23.959008000
16	35.312590000	34.693958000	24.670133000
6	36.940813000	41.958160000	26.409080000
6	37.406802000	43.159008000	26.941131000
6	37.247007000	43.413582000	28.303507000
6	36.603253000	42.460542000	29.086772000

6	35.208004000	35.464758000	26.320387000
6	35.421284000	36.965651000	26.407542000
6	35.845800000	37.780322000	25.357130000
6	36.096354000	39.135378000	25.574192000
6	35.925067000	39.669017000	26.849036000
6	35.194699000	37.593433000	27.637793000
6	36.334718000	41.028049000	27.256638000
7	35.436262000	38.889871000	27.848465000
7	36.169422000	41.301706000	28.572432000
1	33.571875000	33.475069000	22.612869000
1	33.632690000	36.282765000	23.895954000
1	32.898927000	34.839592000	24.631720000
1	36.412407000	39.769599000	24.756465000
1	34.823166000	37.025695000	28.482746000
1	37.023825000	41.756437000	25.349745000
1	37.909595000	43.880010000	26.304124000
1	37.619368000	44.323688000	28.758951000
1	36.431254000	42.606812000	30.146100000
1	35.984393000	34.964205000	26.912475000
1	34.248654000	35.218460000	26.789859000
1	35.990373000	37.350839000	24.371797000
29	35.323992000	39.817010000	29.627944000
6	33.069232000	37.726594000	30.545945000
6	32.435725000	37.529824000	31.758617000
7	32.921792000	38.460466000	32.633193000
6	33.843317000	39.205744000	31.952900000
7	33.937815000	38.762650000	30.686091000
6	32.529715000	38.533486000	34.045927000
1	32.899721000	37.205876000	29.610334000
1	31.687247000	36.810708000	32.056553000
1	33.356534000	38.192227000	34.673674000
1	31.674193000	37.873800000	34.191757000
1	32.236676000	39.551611000	34.305403000
6	34.632501000	40.389184000	32.315195000
6	34.638620000	40.982666000	33.640486000
6	35.079427000	42.244978000	33.841435000
6	35.127111000	42.927673000	35.162990000
8	35.299917000	40.884390000	31.368945000
1	34.285959000	40.389164000	34.475123000
1	35.406498000	42.821797000	32.977229000
1	36.147165000	43.281009000	35.365712000
1	34.807960000	42.280984000	35.984412000
1	34.495621000	43.826438000	35.148072000
6	35.621317000	36.056329000	34.398895000
6	36.042467000	36.986166000	33.421343000
6	36.802562000	38.102645000	33.768009000
6	37.157316000	38.298129000	35.115978000
6	36.722391000	37.342601000	36.083728000
6	35.954341000	36.224055000	35.739331000
6	37.913600000	39.292087000	35.823129000
6	37.920802000	38.925101000	37.144874000
7	37.199841000	37.758551000	37.306464000
1	35.036620000	35.190914000	34.099649000
1	35.763161000	36.828327000	32.381405000
1	37.125609000	38.808194000	33.006221000
1	35.635660000	35.504642000	36.488731000
1	38.404006000	40.159859000	35.404179000
1	38.389757000	39.399513000	37.995625000
1	37.089943000	37.262597000	38.178659000
1	45.536290000	42.596824000	29.630954000
1	43.003924000	41.522609000	26.151740000
1	41.893887000	38.642059000	27.734547000
1	40.976110000	38.610757000	26.223686000
1	33.645186000	47.503279000	24.217518000
1	34.313266000	34.913789000	21.887569000
1	32.564034000	34.857097000	22.144399000
1	30.141162000	37.905712000	24.730300000

^{2}Re$_{proR,C}$

6	40.540016000	38.619760000	29.543907000
1	40.128966000	39.553973000	29.944932000
6	39.438797000	37.585088000	29.294491000
1	38.716103000	37.974025000	28.567098000
1	39.872677000	36.679566000	28.851786000
6	38.690092000	37.207801000	30.593585000
1	39.401403000	36.801115000	31.322744000
1	38.235833000	38.100766000	31.035397000
6	37.575325000	36.215009000	30.331665000
8	36.441712000	36.558127000	29.947460000
7	37.871174000	34.911268000	30.499861000
1	38.787185000	34.599709000	30.788854000
1	37.177153000	34.210875000	30.272051000
6	41.403317000	44.117814000	26.455322000
1	42.431570000	44.309778000	26.783450000
6	41.209723000	44.631246000	25.006993000
1	41.842190000	44.027091000	24.346752000
1	40.169691000	44.443851000	24.717372000
6	41.548351000	46.098471000	24.837259000
6	42.831753000	46.493498000	24.435439000
1	43.580201000	45.735582000	24.213044000
6	43.158773000	47.844468000	24.303297000
1	44.156927000	48.129494000	23.982502000
6	42.202973000	48.825837000	24.573125000
1	42.453967000	49.877205000	24.464998000
6	40.920186000	48.446313000	24.974373000
1	40.168857000	49.204683000	25.179878000
6	40.596617000	47.094284000	25.104728000
1	39.595042000	46.804705000	25.414276000
6	40.388367000	44.728622000	27.413730000
8	39.179683000	44.488420000	27.313257000
7	40.897900000	45.554500000	28.365812000
1	41.881411000	45.782625000	28.310608000
6	40.080427000	46.308096000	29.305536000
1	39.042103000	46.031550000	29.109517000
6	40.453186000	46.039269000	30.778263000
1	41.514030000	46.276095000	30.930138000
1	39.889150000	46.752241000	31.393125000
6	40.169119000	44.627016000	31.244277000
6	41.184552000	43.664148000	31.311223000
1	42.199735000	43.937835000	31.034699000
6	40.917085000	42.364637000	31.747758000
1	41.723578000	41.638745000	31.805568000
6	39.621900000	42.007015000	32.127562000
1	39.420862000	41.001050000	32.489495000
6	38.596967000	42.955964000	32.062304000
1	37.584666000	42.700111000	32.369111000
6	38.870617000	44.252303000	31.621896000
1	38.070211000	44.988098000	31.579447000
6	34.940619000	45.546132000	23.530818000
1	34.671309000	44.711647000	22.877856000
6	36.311154000	46.139349000	23.083047000
1	36.177223000	46.373516000	22.017648000
6	36.631966000	47.455638000	23.807764000
1	37.533592000	47.913240000	23.387532000
1	35.816676000	48.183507000	23.705900000
1	36.818368000	47.294318000	24.876936000

6	37.486150000	45.147910000	23.196694000
1	38.392276000	45.682225000	22.883288000
1	37.641472000	44.883707000	24.252322000
6	37.348655000	43.870276000	22.359390000
1	36.570554000	43.203311000	22.745024000
1	38.287664000	43.305640000	22.364139000
1	37.114281000	44.102400000	21.313221000
6	34.922779000	45.016500000	24.951814000
8	35.191724000	43.835811000	25.239079000
7	34.610878000	45.892966000	25.936064000
1	34.405035000	46.852074000	25.692991000
6	34.667367000	45.530472000	27.344398000
1	35.711952000	45.416207000	27.667692000
6	33.959475000	46.610742000	28.188391000
1	34.310973000	47.592520000	27.844980000
1	32.881272000	46.573039000	27.982555000
6	34.185140000	46.490516000	29.701027000
1	33.516051000	47.179449000	30.226457000
1	33.948370000	45.483336000	30.055364000
16	35.903266000	46.817507000	30.262778000
6	35.939979000	48.645468000	30.216426000
1	36.924914000	48.950488000	30.579002000
1	35.810902000	49.031645000	29.201580000
1	35.176398000	49.067808000	30.875859000
6	33.968262000	44.172384000	27.572297000
8	34.189155000	43.418618000	28.467741000
7	32.115213000	42.419053000	24.443431000
1	32.976675000	42.656837000	24.954922000
6	32.444592000	41.441423000	23.444449000
1	31.783852000	41.609188000	22.576325000
6	33.898257000	41.415745000	22.936648000
1	34.036835000	40.516841000	22.331271000
1	34.064736000	42.290318000	22.292660000
8	34.848722000	41.373329000	23.990519000
1	35.004559000	42.280962000	24.328058000
6	31.995269000	40.019843000	23.946783000
8	32.493899000	38.997618000	23.490447000
7	30.991080000	40.059000000	24.855569000
1	30.671273000	40.989622000	25.108436000
6	30.261489000	38.887929000	25.314542000
1	30.816728000	38.012239000	24.971402000
6	30.097686000	38.868598000	26.839949000
1	31.083446000	38.814553000	27.314446000
1	29.628273000	39.803999000	27.169814000
6	29.231384000	37.693675000	27.325434000
1	29.028038000	37.815408000	28.396849000
1	28.259159000	37.701418000	26.818319000
6	29.924395000	36.343573000	27.162979000
8	31.070742000	36.142977000	27.578150000
7	29.209691000	35.373421000	26.543284000
1	28.262444000	35.516366000	26.224974000
1	29.600521000	34.442671000	26.472232000
6	31.557423000	35.148994000	21.866676000
6	31.914440000	35.509995000	23.309264000
16	33.412115000	34.559112000	23.815889000
6	36.713144000	40.917363000	26.486235000
6	37.398504000	41.954069000	27.118382000
6	37.512297000	41.953845000	28.508776000
6	36.923006000	40.916359000	29.223366000
6	33.574932000	35.023031000	25.575493000
6	34.221620000	36.359633000	25.898596000
6	34.675813000	37.276297000	24.949123000
6	35.284225000	38.466223000	25.348864000
6	35.441130000	38.730541000	26.707561000
6	34.375601000	36.718096000	27.243753000
6	36.153913000	39.899586000	27.260751000
7	34.958860000	37.857618000	27.628764000
7	36.267653000	39.916124000	28.613097000
1	31.346862000	34.080050000	21.758487000
1	32.108448000	36.583109000	23.397342000
1	31.095215000	35.244292000	23.987966000
1	35.604094000	39.189460000	24.609639000
1	34.027325000	36.065648000	28.037212000
1	36.593612000	40.910110000	25.410322000
1	37.850505000	42.762916000	26.554708000
1	38.042565000	42.747805000	29.021617000
1	36.965793000	40.869292000	30.305126000
1	34.178077000	34.221902000	26.019801000
1	32.595524000	34.982824000	26.066411000
1	34.556841000	37.058612000	23.894064000
29	35.286612000	38.403775000	29.533711000
6	32.438329000	37.064775000	30.316738000
6	31.790328000	36.923627000	31.530760000
7	32.555122000	37.541335000	32.480216000
6	33.651458000	38.052281000	31.837278000
7	33.587279000	37.754501000	30.527767000
6	32.181648000	37.623666000	33.896963000
1	32.109792000	36.730863000	29.338762000
1	30.860662000	36.436457000	31.785053000
1	32.963987000	37.188596000	34.522004000
1	31.265340000	37.049425000	34.036320000
1	31.997400000	38.665184000	34.171861000
6	34.796157000	38.844603000	32.297727000
6	34.958868000	39.297384000	33.666098000
6	36.009396000	40.063423000	34.035309000
6	36.228264000	40.609232000	35.401614000
8	35.643912000	39.125699000	31.405714000
1	34.196658000	39.048286000	34.392448000
1	36.748729000	40.315518000	33.277419000
1	37.216446000	40.315862000	35.779803000
1	35.463235000	40.284832000	36.111819000
1	36.229630000	41.707121000	35.360541000
6	31.173822000	41.426836000	34.160734000
6	31.299440000	41.054674000	32.803840000
6	32.325604000	41.559595000	32.009836000
6	33.243628000	42.462999000	32.574781000
6	33.093998000	42.826385000	33.944849000
6	32.070730000	42.313863000	34.749414000
6	34.372558000	43.194561000	32.067257000
6	34.850029000	43.961658000	33.101177000
7	34.099922000	43.726816000	34.239595000
1	30.346202000	41.039347000	34.748626000
1	30.557976000	40.390587000	32.366190000
1	32.395813000	41.294471000	30.957556000
1	31.960912000	42.614019000	35.787975000
1	34.752742000	43.176324000	31.054880000
1	35.657915000	44.680103000	33.116463000
1	34.177750000	44.249901000	35.099270000
1	40.189034000	47.380652000	29.097424000
1	41.247711000	43.032600000	26.474256000
1	41.284135000	38.245030000	30.256544000
1	41.063975000	38.857962000	28.612608000
1	34.167756000	46.315431000	23.410280000
1	32.369070000	35.409155000	21.179559000
1	30.666443000	35.705540000	21.556195000
1	29.271518000	38.858948000	24.835457000

^{2}**TS1$_{ProS,C}$**

6	38.315498000	35.574296000	28.356545000
1	38.585693000	36.166512000	27.473886000
6	37.707273000	36.457104000	29.449821000
1	36.780799000	36.922949000	29.093429000
1	37.434132000	35.837167000	30.314072000
6	38.670707000	37.570751000	29.908977000
1	39.615156000	37.129757000	30.250645000
1	38.904943000	38.225877000	29.062090000
6	38.045428000	38.427639000	30.997993000
8	37.119183000	39.216543000	30.774076000
7	38.541878000	38.250892000	32.248793000
1	39.258982000	37.569212000	32.446969000
1	38.099523000	38.719613000	33.029885000
6	43.667036000	39.919002000	25.447042000
1	44.646891000	39.892839000	24.956764000
6	42.928597000	38.581053000	25.219908000
1	43.473651000	37.799215000	25.760693000
1	41.939236000	38.658403000	25.685365000
6	42.802459000	38.192127000	23.760112000
6	43.706739000	37.288067000	23.187050000
1	44.487180000	36.847783000	23.804206000
6	43.615551000	36.937742000	21.838685000
1	44.324669000	36.231766000	21.415276000
6	42.612337000	37.487695000	21.038705000
1	42.536742000	37.212595000	19.990564000
6	41.703839000	38.389937000	21.596410000
1	40.917231000	38.819784000	20.981940000
6	41.799718000	38.739293000	22.944785000
1	41.095384000	39.452403000	23.366666000
6	42.852585000	41.125279000	24.992793000
8	41.682468000	41.291521000	25.364480000
7	43.491809000	41.999634000	24.180143000
1	44.425092000	41.761535000	23.872288000
6	42.895033000	43.219937000	23.650876000
1	41.871224000	43.252189000	24.028154000
6	43.670971000	44.493822000	24.047942000
1	44.714382000	44.391069000	23.721531000
1	43.250203000	45.323843000	23.467631000
6	43.618770000	44.822918000	25.524692000
6	44.561200000	44.297650000	26.420328000
1	45.358872000	43.660675000	26.045965000
6	44.505508000	44.598971000	27.782304000
1	45.254355000	44.191781000	28.456309000
6	43.505910000	45.442529000	28.272864000
1	43.478995000	45.700897000	29.328232000
6	42.562543000	45.977395000	27.390643000
1	41.798653000	46.659863000	27.755611000
6	42.618402000	45.664397000	26.030694000
1	41.890150000	46.098545000	25.348962000
6	33.077446000	46.743619000	28.293246000
1	33.740352000	46.349887000	27.518379000
6	33.786520000	47.905881000	29.050864000
1	34.110902000	48.596574000	28.259773000
6	32.819096000	48.674197000	29.964218000
1	33.308942000	49.558485000	30.385254000
1	31.935915000	49.022029000	29.414003000
1	32.478283000	48.058414000	30.805800000
6	35.043608000	47.466269000	29.827416000
1	35.455604000	48.358528000	30.316271000
1	34.751349000	46.782610000	30.636904000
6	36.137901000	46.811164000	28.977321000
1	35.820186000	45.835169000	28.595848000
1	37.045293000	46.652574000	29.571694000
1	36.413727000	47.441945000	28.123250000
6	32.681678000	45.566871000	29.164997000
8	33.424307000	44.591551000	29.371834000
7	31.460529000	45.615214000	29.754795000
1	30.830707000	46.375676000	29.538265000
6	30.999790000	44.536648000	30.609278000
1	31.660639000	44.442594000	31.480762000
6	29.556762000	44.796745000	31.082009000
1	29.514603000	45.826874000	31.458054000
1	28.874038000	44.742814000	30.222729000
6	29.065920000	43.830390000	32.168196000
1	28.011420000	44.028106000	32.384380000
1	29.133909000	42.795723000	31.821508000
16	29.999419000	43.877918000	33.749878000
6	29.283202000	45.370223000	34.529098000
1	29.756233000	45.470215000	35.509238000
1	29.486291000	46.275349000	33.950194000
1	28.205370000	45.251523000	34.670804000
6	31.087440000	43.183942000	29.865264000
8	31.095042000	42.108161000	30.375614000
7	32.293446000	43.434498000	26.316603000
1	32.379185000	43.251812000	27.327130000
6	33.532088000	43.040233000	25.709839000
1	33.667429000	43.620364000	24.782474000
6	34.798388000	43.202489000	26.568738000
1	35.628167000	42.712054000	26.055601000
1	35.030773000	44.268343000	26.680801000
8	34.655509000	42.593924000	27.850367000
1	34.324681000	43.264278000	28.484675000
6	33.424015000	41.551477000	25.210980000
8	34.431395000	40.872551000	25.045608000
7	32.161740000	41.146111000	24.948619000
1	31.426368000	41.817017000	25.147821000
6	31.839731000	39.813006000	24.459542000
1	32.792580000	39.346581000	24.202580000
6	31.096028000	38.961572000	25.500176000
1	31.634924000	39.014260000	26.450698000
1	30.092129000	39.369278000	25.670418000
6	30.977358000	37.491719000	25.074382000
1	30.347124000	36.951244000	25.792958000
1	30.479178000	37.405156000	24.100677000
6	32.335159000	36.788396000	25.046636000
8	33.222693000	37.038446000	25.866596000
7	32.502473000	35.868032000	24.064481000
1	31.768081000	35.632812000	23.413045000
1	33.356621000	35.326323000	24.033500000
6	35.432966000	38.827278000	21.117136000
6	35.319502000	38.412426000	22.584320000
16	36.614401000	37.159013000	22.969154000
6	39.649481000	42.280523000	27.661409000
6	39.918640000	43.361835000	28.499556000
6	38.997885000	43.713447000	29.487530000
6	37.830340000	42.964806000	29.605899000
6	36.515748000	37.081617000	24.804050000
6	37.082201000	38.278202000	25.531301000
6	38.358159000	38.789326000	25.272459000
6	38.842236000	39.869798000	26.005458000
6	38.040192000	40.423595000	27.006979000
6	36.340068000	38.904082000	26.539640000
6	38.454437000	41.573488000	27.838945000
7	36.805741000	39.926624000	27.268276000
7	37.574110000	41.925142000	28.803331000

1	35.323870000	37.969051000	20.445248000
1	35.440619000	39.274397000	23.246413000
1	34.339190000	37.965457000	22.782807000
1	39.831260000	40.269534000	25.802260000
1	35.334553000	38.571258000	26.756506000
1	40.355193000	42.006645000	26.882852000
1	40.837998000	43.926073000	28.369858000
1	39.174053000	44.553657000	30.150773000
1	37.067631000	43.180530000	30.345317000
1	37.098053000	36.188610000	25.061196000
1	35.483942000	36.901127000	25.120798000
1	38.967159000	38.351602000	24.486301000
29	35.886061000	40.797567000	28.894591000
6	33.464830000	38.665187000	28.744414000
6	32.460336000	38.375714000	29.636301000
7	32.624618000	39.204020000	30.719216000
6	33.718748000	39.980387000	30.466799000
7	34.238280000	39.661359000	29.271682000
6	31.716270000	39.241593000	31.865470000
1	33.624433000	38.213134000	27.776065000
1	31.652444000	37.661752000	29.588057000
1	32.213634000	38.867456000	32.764377000
1	30.864224000	38.598469000	31.642738000
1	31.362470000	40.261142000	32.021816000
6	34.363015000	41.051130000	31.246225000
6	34.020514000	41.375830000	32.563492000
6	34.678913000	42.415184000	33.255735000
6	34.081813000	42.906801000	34.550653000
8	35.315958000	41.651915000	30.621127000
1	33.310320000	40.765537000	33.104728000
1	35.121443000	43.179685000	32.622736000
1	34.716680000	43.642043000	35.051207000
1	33.881485000	42.084418000	35.244725000
1	33.120133000	43.388379000	34.328017000
6	36.210721000	38.829402000	36.818292000
6	35.816166000	38.380035000	35.545198000
6	35.891461000	39.210702000	34.426670000
6	36.376257000	40.515185000	34.592832000
6	36.777217000	40.936448000	35.881076000
6	36.699665000	40.120397000	37.008737000
6	36.543305000	41.650606000	33.683715000
6	37.163898000	42.648004000	34.465987000
7	37.245582000	42.249759000	35.753042000
1	36.138693000	38.158278000	37.668210000
1	35.448484000	37.364149000	35.434828000
1	35.593982000	38.857641000	33.444016000
1	37.010618000	40.466311000	37.989949000
1	36.735849000	41.541069000	32.621427000
1	37.499491000	43.634376000	34.173897000
1	37.581935000	42.821458000	36.517442000
1	42.851678000	43.149369000	22.556808000
1	43.846583000	40.050740000	26.522186000
1	39.223340000	35.070529000	28.708094000
1	37.608418000	34.799827000	28.040674000
1	32.191961000	47.144547000	27.784782000
1	36.398774000	39.299261000	20.910921000
1	34.643704000	39.546882000	20.872350000
1	31.246275000	39.898998000	23.540482000

^{2}TS1$_{ProR,C}$

6	38.643256000	43.243216000	27.628862000
1	37.883490000	43.705400000	28.271121000
6	38.573417000	41.715531000	27.699448000
1	37.594514000	41.370380000	27.346898000
1	39.320994000	41.278846000	27.025079000
6	38.808619000	41.182912000	29.129816000
1	39.793521000	41.503290000	29.488713000
1	38.057968000	41.595253000	29.813323000
6	38.681269000	39.673646000	29.183809000
8	37.581249000	39.093530000	29.130611000
7	39.826647000	38.967909000	29.253112000
1	40.738907000	39.407455000	29.285702000
1	39.778035000	37.957182000	29.234322000
6	39.782551000	45.401179000	31.084474000
1	40.272388000	45.259924000	30.113714000
6	40.462570000	46.574957000	31.841118000
1	40.279625000	47.489598000	31.264525000
1	39.962804000	46.703958000	32.807873000
6	41.951610000	46.381911000	32.046943000
6	42.843991000	46.529912000	30.973858000
1	42.466027000	46.827344000	29.997337000
6	44.214743000	46.326696000	31.147903000
1	44.892422000	46.465282000	30.309814000
6	44.717005000	45.971802000	32.403379000
1	45.784208000	45.826491000	32.543618000
6	43.840029000	45.827518000	33.480747000
1	44.223725000	45.570887000	34.464392000
6	42.469327000	46.030628000	33.300713000
1	41.794375000	45.926295000	34.147388000
6	39.806107000	44.112811000	31.894039000
8	38.928250000	43.846764000	32.728177000
7	40.863800000	43.295014000	31.676771000
1	41.584236000	43.606626000	31.038099000
6	41.164800000	42.141989000	32.521652000
1	40.569360000	41.276064000	32.200868000
6	42.664010000	41.794856000	32.475544000
1	43.237292000	42.666824000	32.811812000
1	42.838375000	40.997237000	33.207298000
6	43.140183000	41.350399000	31.106629000
6	43.773785000	42.244647000	30.232652000
1	43.979535000	43.262095000	30.560887000
6	44.179637000	41.837882000	28.957353000
1	44.676672000	42.544997000	28.299227000
6	43.970539000	40.523915000	28.539992000
1	44.303982000	40.201081000	27.558084000
6	43.357872000	39.614499000	29.409311000
1	43.239675000	38.575346000	29.110249000
6	42.942224000	40.027064000	30.678064000
1	42.492840000	39.306966000	31.359659000
6	28.379187016	45.845778093	25.792331375
1	28.191480685	44.878315815	25.316629135
6	29.883702867	46.218408525	25.644949987
1	30.058707951	46.246673734	24.560086086
6	30.171610694	47.620796518	26.201569284
1	31.205972495	47.913571167	25.992491511
1	29.518554889	48.377634824	25.749355006
1	30.033295827	47.659657221	27.289521672
6	30.850283792	45.175245102	26.240536737
1	31.870563595	45.535771297	26.053600163
1	30.728586918	45.144363807	27.330684946
6	30.707512596	43.759000631	25.671986405
1	29.760655140	43.298007330	25.972648284

1	31.514441979	43.113723184	26.036374490
1	30.756262706	43.758353347	24.576018985
6	27.908283008	45.729998625	27.227747290
8	28.252003008	44.881840625	28.064643290
7	27.755979281	46.770491930	28.088358008
1	27.534794839	47.690836551	27.734765216
6	27.894870971	46.572284998	29.519438314
1	28.926097799	46.282885726	29.763196085
6	27.523760355	47.854919119	30.287545959
1	28.075743811	48.685670490	29.829336690
1	26.455067898	48.067806516	30.148500065
6	27.827382463	47.800587130	31.791694949
1	27.464804854	48.716332206	32.269616719
1	27.301638385	46.964178609	32.261122520
16	29.593736473	47.566488116	32.235996402
6	30.258234018	49.238620463	31.908641791
1	31.314680926	49.215241869	32.187607275
1	30.185233498	49.512020785	30.852391986
1	29.748726866	49.986104035	32.523533429
6	26.996826560	45.405543433	29.979061160
8	27.159151634	44.731359575	30.949462583
7	29.843390000	41.234909000	25.065750000
1	30.262478000	42.169093000	24.969945000
6	30.288541000	40.407233000	23.977205000
1	29.391682000	40.148474000	23.380614000
6	31.303539000	41.021098000	23.010413000
1	31.648244000	40.247913000	22.313638000
1	30.801905000	41.813327000	22.439026000
8	32.391071000	41.573723000	23.748187000
1	32.783760000	42.286988000	23.204942000
6	30.739988000	39.010360000	24.514819000
8	31.468626000	38.279582000	23.849958000
7	30.203364000	38.690519000	25.716884000
1	29.617271000	39.407162000	26.134674000
6	30.286908000	37.367110000	26.311683000
1	31.021469000	36.805335000	25.729743000
6	30.679167000	37.415897000	27.794362000
1	31.675767000	37.858392000	27.898715000
1	29.977660000	38.062649000	28.336768000
6	30.667672000	36.025609000	28.456232000
1	30.808240000	36.143032000	29.537385000
1	29.693199000	35.543467000	28.311676000
6	31.804734000	35.134858000	27.964203000
8	32.987118000	35.474481000	28.065537000
7	31.445085000	33.951596000	27.407577000
1	30.483980000	33.650375000	27.344481000
1	32.167911000	33.306429000	27.116185000
6	32.856023000	34.556695000	22.333099000
6	33.204732000	35.114931000	23.713085000
16	35.031713000	35.349600000	23.824713000
6	33.543503000	42.248454000	26.752862000
6	33.426153000	43.482912000	27.389571000
6	34.001531000	43.666524000	28.648707000
6	34.660507000	42.592274000	29.239005000
6	35.241003000	35.840802000	25.573216000
6	34.954939000	37.286627000	25.937031000
6	34.418198000	38.232410000	25.064405000
6	34.156031000	39.532072000	25.501693000
6	34.442772000	39.873712000	26.821027000
6	35.218146000	37.713406000	27.246465000
6	34.238033000	41.219635000	27.397815000
7	34.971735000	38.955824000	27.669474000
7	34.772836000	41.403663000	28.629444000

1	33.344868000	33.593849000	22.152361000
1	32.693708000	36.068890000	23.875395000
1	32.901355000	34.416020000	24.501331000
1	33.720308000	40.253137000	24.815894000
1	35.642132000	37.035569000	27.980001000
1	33.081145000	42.082849000	25.784209000
1	32.878404000	44.283182000	26.901513000
1	33.929141000	44.614636000	29.170761000
1	35.111630000	42.656438000	30.223502000
1	36.285218000	35.606931000	25.812109000
1	34.623197000	35.199244000	26.213121000
1	34.195214000	37.958134000	24.040147000
29	35.467969000	39.661733000	29.497568000
6	34.341851000	36.873544000	30.685153000
6	34.314629000	36.379507000	31.966596000
7	34.865435000	37.339288000	32.788628000
6	35.213597000	38.396750000	31.994533000
7	34.904566000	38.119003000	30.721501000
6	35.011941000	37.183936000	34.235517000
1	33.974156000	36.420626000	29.772057000
1	33.953153000	35.442218000	32.362323000
1	36.066671000	37.213532000	34.519576000
1	34.597990000	36.215380000	34.517707000
1	34.464717000	37.969138000	34.763134000
6	35.828090000	39.708111000	32.285704000
6	36.273695000	40.089369000	33.546524000
6	36.963776000	41.312855000	33.773701000
6	37.724114000	41.471963000	35.067051000
8	35.917286000	40.485941000	31.246198000
1	36.074329000	39.455582000	34.400064000
1	37.446147000	41.757956000	32.908165000
1	38.462906000	40.663154000	35.142436000
1	37.070838000	41.395437000	35.942897000
1	38.264747000	42.419557000	35.096542000
6	33.166703000	42.626235000	37.282516000
6	32.833892000	41.683950000	36.293661000
6	33.517874000	41.636302000	35.078466000
6	34.552419000	42.551304000	34.855007000
6	34.858111000	43.493485000	35.861104000
6	34.189380000	43.552490000	37.081422000
6	35.492730000	42.771644000	33.755024000
6	36.235917000	43.922029000	34.121832000
7	35.895971000	44.299580000	35.364429000
1	32.613233000	42.639771000	38.216187000
1	32.021341000	40.987934000	36.479805000
1	33.242382000	40.914291000	34.315387000
1	34.441740000	44.286953000	37.840356000
1	35.275814000	42.536503000	32.720178000
1	37.050624000	44.389917000	33.580445000
1	36.349538000	45.040073000	35.884498000
1	40.861979000	42.381742000	33.545691000
1	38.733629000	45.650745000	30.896718000
1	39.624737000	43.612604000	27.949355000
1	38.475083000	43.594717000	26.605570000
1	27.778371447	46.591872935	25.257853697
1	33.155702000	35.248081000	21.538820000
1	31.773122000	34.410545000	22.256266000
1	29.320233000	36.852340000	26.204093000

^{2}Int1$_{ProS,C}$

6	38.253290000	35.464338000	28.326479000
1	38.504404000	36.057499000	27.438959000
6	37.677996000	36.348647000	29.436497000
1	36.746557000	36.820671000	29.101727000
1	37.420054000	35.728614000	30.305372000
6	38.661831000	37.454747000	29.873698000
1	39.608796000	37.003024000	30.194871000
1	38.881561000	38.110066000	29.024434000
6	38.072538000	38.314924000	30.979842000
8	37.254517000	39.213969000	30.766724000
7	38.478794000	38.008319000	32.242025000
1	39.101980000	37.237312000	32.430493000
1	38.033059000	38.466402000	33.026057000
6	43.598980000	39.899382000	25.571747000
1	44.601337000	39.890409000	25.128422000
6	42.884173000	38.559856000	25.283316000
1	43.415150000	37.770516000	25.827268000
1	41.875570000	38.617044000	25.708213000
6	42.822248000	38.206862000	23.810356000
6	43.780542000	37.355361000	23.243843000
1	44.555306000	36.925680000	23.875678000
6	43.749463000	37.043027000	21.883454000
1	44.499152000	36.376871000	21.465433000
6	42.753781000	37.578959000	21.064702000
1	42.724875000	37.333046000	20.007028000
6	41.792087000	38.428357000	21.615732000
1	41.010254000	38.845901000	20.986861000
6	41.827274000	38.739462000	22.976313000
1	41.079787000	39.409890000	23.394194000
6	42.794369000	41.105583000	25.100465000
8	41.613856000	41.265397000	25.439669000
7	43.452867000	41.985496000	24.308885000
1	44.392945000	41.748855000	24.021396000
6	42.862464000	43.199945000	23.759575000
1	41.832103000	43.232614000	24.118727000
6	43.628605000	44.478669000	24.160034000
1	44.677257000	44.376803000	23.850550000
1	43.214716000	45.303948000	23.567952000
6	43.552800000	44.817525000	25.633546000
6	44.480627000	44.298151000	26.547614000
1	45.283849000	43.658658000	26.190026000
6	44.402818000	44.607875000	27.906416000
1	45.140602000	44.204779000	28.594902000
6	43.395395000	45.453807000	28.375541000
1	43.350642000	45.717945000	29.428863000
6	42.466607000	45.983546000	27.475213000
1	41.697031000	46.668412000	27.823476000
6	42.544408000	45.662400000	26.118073000
1	41.827253000	46.092361000	25.421931000
6	33.318168000	46.896238000	28.374013000
1	34.058766000	46.553133000	27.646811000
6	33.921461000	48.050251000	29.230700000
1	34.272468000	48.788941000	28.496309000
6	32.859192000	48.735730000	30.103030000
1	33.281325000	49.611880000	30.606466000
1	32.008466000	49.082955000	29.503568000
1	32.477522000	48.063005000	30.880893000
6	35.137992000	47.624222000	30.075986000
1	35.480640000	48.509874000	30.626705000
1	34.817504000	46.896839000	30.834870000
6	36.311153000	47.043558000	29.278361000
1	36.056614000	46.072485000	28.841454000

1	37.183320000	46.897888000	29.926996000
1	36.617354000	47.716606000	28.468092000
6	32.890394000	45.677123000	29.171608000
8	33.654936000	44.732261000	29.425066000
7	31.614991000	45.667625000	29.639318000
1	30.983791000	46.412013000	29.376208000
6	31.108278000	44.570451000	30.440904000
1	31.719810000	44.458992000	31.345936000
6	29.641473000	44.826225000	30.839432000
1	29.580583000	45.854436000	31.217988000
1	29.004057000	44.778346000	29.945650000
6	29.092359000	43.855739000	31.893651000
1	28.030187000	44.060616000	32.060823000
1	29.169199000	42.823480000	31.542327000
16	29.950903000	43.884158000	33.517320000
6	29.211601000	45.376105000	34.275236000
1	29.636338000	45.461089000	35.278636000
1	29.452542000	46.284957000	33.717043000
1	28.126844000	45.267776000	34.362025000
6	31.239943000	43.226471000	29.686862000
8	31.134631000	42.145654000	30.172907000
7	32.188824000	43.394826000	26.294910000
1	32.236159000	43.242248000	27.314635000
6	33.451813000	43.000212000	25.741788000
1	33.628078000	43.584911000	24.823792000
6	34.679641000	43.154111000	26.654545000
1	35.537407000	42.692535000	26.159599000
1	34.888330000	44.218637000	26.810774000
8	34.484434000	42.505983000	27.909936000
1	34.251908000	43.181674000	28.578465000
6	33.367567000	41.515941000	25.226489000
8	34.385882000	40.852418000	25.064234000
7	32.113381000	41.096803000	24.947297000
1	31.368795000	41.758710000	25.142364000
6	31.812643000	39.763138000	24.447413000
1	32.773498000	39.309955000	24.196182000
6	31.070341000	38.896489000	25.476329000
1	31.607482000	38.938935000	26.428317000
1	30.064866000	39.299284000	25.649830000
6	30.955696000	37.431639000	25.031851000
1	30.327578000	36.880875000	25.744447000
1	30.457464000	37.356188000	24.057203000
6	32.315292000	36.732691000	24.995938000
8	33.193578000	36.959131000	25.831962000
7	32.495595000	35.844284000	23.986304000
1	31.768546000	35.627628000	23.320472000
1	33.353133000	35.308577000	23.945007000
6	35.442449000	38.808431000	21.153085000
6	35.307071000	38.375196000	22.613155000
16	36.588232000	37.107602000	22.998426000
6	39.514717000	42.274785000	27.679822000
6	39.745075000	43.388830000	28.485454000
6	38.799145000	43.751673000	29.445864000
6	37.651165000	42.974891000	29.575427000
6	36.477127000	37.016883000	24.832580000
6	37.028643000	38.214203000	25.569854000
6	38.303209000	38.735259000	25.324155000
6	38.768678000	39.822745000	26.058834000
6	37.948944000	40.372513000	27.048512000
6	36.272086000	38.832959000	26.572067000
6	38.333905000	41.545005000	27.863739000
7	36.719886000	39.861116000	27.303135000
7	37.435329000	41.901299000	28.807346000

1	35.337145000	37.959763000	20.468497000
1	35.425475000	39.228033000	23.287663000
1	34.321141000	37.933256000	22.792958000
1	39.755363000	40.232043000	25.863501000
1	35.267725000	38.490452000	26.780398000
1	40.237241000	41.994834000	26.919307000
1	40.651954000	43.971719000	28.349876000
1	38.940121000	44.623791000	30.075733000
1	36.867971000	43.194182000	30.292715000
1	37.064316000	36.126115000	25.086401000
1	35.444437000	36.827099000	25.140289000
1	38.923405000	38.301560000	24.544482000
29	35.786202000	40.711526000	28.943346000
6	33.375001000	38.570342000	28.758949000
6	32.329803000	38.321445000	29.609313000
7	32.479153000	39.160342000	30.693451000
6	33.605452000	39.896669000	30.479277000
7	34.160886000	39.551697000	29.309892000
6	31.527630000	39.258537000	31.796687000
1	33.561387000	38.105026000	27.802053000
1	31.501696000	37.632876000	29.538529000
1	31.958589000	38.860784000	32.720059000
1	30.645393000	38.670635000	31.540112000
1	31.234126000	40.299930000	31.937640000
6	34.251524000	40.948294000	31.283324000
6	33.921969000	41.273219000	32.574243000
6	34.687905000	42.318678000	33.288954000
6	34.030956000	42.777676000	34.590249000
8	35.244567000	41.541270000	30.662418000
1	33.160673000	40.731703000	33.119266000
1	34.858434000	43.171730000	32.622813000
1	34.603416000	43.569841000	35.086823000
1	33.909898000	41.949789000	35.297213000
1	33.034456000	43.175266000	34.369839000
6	36.975246000	38.988506000	36.689686000
6	36.205190000	38.513530000	35.616008000
6	35.907513000	39.322902000	34.512990000
6	36.389440000	40.632218000	34.499513000
6	37.163231000	41.073511000	35.585878000
6	37.475081000	40.291491000	36.692927000
6	36.243292000	41.763107000	33.524741000
6	37.067868000	42.803727000	34.139376000
7	37.538276000	42.410141000	35.307125000
1	37.186819000	38.334837000	37.529731000
1	35.831339000	37.494438000	35.644804000
1	35.313636000	38.947216000	33.687157000
1	38.075140000	40.665752000	37.516682000
1	36.528272000	41.534171000	32.484134000
1	37.250458000	43.807912000	33.776052000
1	38.082227000	42.992749000	35.936630000
1	42.837722000	43.121422000	22.665351000
1	43.724691000	40.011700000	26.656668000
1	39.166970000	34.955025000	28.654338000
1	37.533876000	34.694898000	28.026752000
1	32.465969000	47.290125000	27.806664000
1	36.413748000	39.277093000	20.965752000
1	34.660882000	39.536145000	20.907501000
1	31.226308000	39.847146000	23.523379000

^{2}Int1$_{ProR,C}$

6	39.099076000	43.266350000	29.070648000
1	38.415095000	43.604666000	29.858665000
6	38.776573000	41.835192000	28.631728000
1	37.786732000	41.795197000	28.162071000
1	39.499828000	41.513060000	27.871756000
6	38.809304000	40.850097000	29.819890000
1	39.776190000	40.924228000	30.331136000
1	38.020125000	41.106894000	30.534016000
6	38.554245000	39.426281000	29.367537000
8	37.421137000	39.005606000	29.076070000
7	39.636427000	38.630088000	29.254828000
1	40.569686000	38.960555000	29.468264000
1	39.518499000	37.688014000	28.905500000
6	40.021787000	45.908321000	31.839431000
1	40.051494000	45.719101000	30.758223000
6	41.103636000	46.948991000	32.218836000
1	40.773907000	47.911020000	31.805759000
1	41.118543000	47.077910000	33.307598000
6	42.511800000	46.661524000	31.723628000
6	42.780488000	46.529720000	30.351081000
1	41.973198000	46.640284000	29.630111000
6	44.075672000	46.281099000	29.895868000
1	44.265540000	46.195685000	28.829464000
6	45.130554000	46.158368000	30.805902000
1	46.139335000	45.970456000	30.450370000
6	44.880449000	46.292124000	32.171579000
1	45.694669000	46.213216000	32.886346000
6	43.580877000	46.542045000	32.624756000
1	43.397855000	46.663272000	33.690481000
6	40.056784000	44.550472000	32.541394000
8	39.005570000	43.973103000	32.867905000
7	41.269508000	43.994412000	32.743651000
1	42.088552000	44.549550000	32.522392000
6	41.477490000	42.730490000	33.446172000
1	40.640877000	42.069379000	33.206039000
6	42.824271000	42.081873000	33.071631000
1	43.615452000	42.840586000	33.149285000
1	43.048652000	41.335488000	33.841643000
6	42.874003000	41.417307000	31.709211000
6	43.034636000	42.162493000	30.529143000
1	43.135448000	43.243622000	30.580096000
6	43.102503000	41.531297000	29.285874000
1	43.242957000	42.125307000	28.387072000
6	43.029864000	40.137696000	29.197917000
1	43.124160000	39.645662000	28.233533000
6	42.879161000	39.383134000	30.365162000
1	42.869074000	38.296425000	30.318222000
6	42.791974000	40.021895000	31.606459000
1	42.694094000	39.423927000	32.509709000
6	28.030872095	45.316004881	26.211431705
1	27.660597617	44.302057893	26.031773639
6	28.371516308	45.774759791	24.762679207
1	27.456825559	45.578074866	24.185513071
6	28.647191532	47.284701315	24.702141709
1	28.779704872	47.610910337	23.665103411
1	27.817505003	47.864754691	25.125197699
1	29.560717999	47.550697588	25.248933436
6	29.513756597	44.975219423	24.104679850
1	29.650747118	45.379082913	23.092724495
1	30.450964422	45.166530157	24.642732771
6	29.278725361	43.463427654	24.013138638
1	29.288896016	42.993232884	25.002095788

1	30.064704024	42.986285634	23.417435608
1	28.318716531	43.233889748	23.534309024
6	28.705623968	45.303576490	27.567871895
8	29.137345432	44.551263428	28.454151889
7	27.922600375	45.405371993	28.673660379
1	27.063374276	45.935924146	28.638451130
6	28.300802503	44.745165853	29.910159702
1	29.216778196	45.195067948	30.316561940
6	27.169376718	44.846890465	30.950436272
1	26.855578518	45.897632509	30.998381214
1	26.301101192	44.271366009	30.601760297
6	27.558027893	44.360277270	32.353830059
1	26.677021889	44.379299501	33.003386064
1	27.909315705	43.324960876	32.319008201
16	28.910033138	45.298107409	33.169257948
6	28.025894537	46.821548395	33.661545934
1	28.747396573	47.441577598	34.199619483
1	27.662380205	47.385082430	32.797772393
1	27.193342620	46.588871290	34.331784798
6	28.626564329	43.262450054	29.634968643
8	29.336056738	42.562974150	30.290289454
7	30.018108000	41.287529000	24.700568000
1	30.476370000	42.204842000	24.621765000
6	30.479854000	40.442632000	23.632394000
1	29.600882000	40.211750000	22.999126000
6	31.555247000	41.021771000	22.710305000
1	31.903230000	40.237258000	22.027798000
1	31.105881000	41.831585000	22.119909000
8	32.625846000	41.534691000	23.498090000
1	33.082168000	42.221867000	22.971384000
6	30.863137000	39.031984000	24.187928000
8	31.586796000	38.274464000	23.548659000
7	30.273722000	38.735122000	25.371066000
1	29.699415000	39.473518000	25.766539000
6	30.281545000	37.409766000	25.968082000
1	31.020324000	36.819745000	25.420505000
6	30.605614000	37.445306000	27.467456000
1	31.614508000	37.843439000	27.619477000
1	29.907894000	38.123317000	27.975673000
6	30.502137000	36.058590000	28.128410000
1	30.596901000	36.172543000	29.214887000
1	29.515706000	35.618655000	27.937672000
6	31.622056000	35.118636000	27.691166000
8	32.811083000	35.411440000	27.839178000
7	31.237853000	33.944905000	27.128918000
1	30.269013000	33.680464000	27.030343000
1	31.946181000	33.268908000	26.874339000
6	32.973795000	34.471404000	22.185735000
6	33.266738000	35.059908000	23.566219000
16	35.091421000	35.244484000	23.769823000
6	33.616527000	42.199739000	26.564135000
6	33.492650000	43.441625000	27.183900000
6	33.986920000	43.617184000	28.478161000
6	34.572459000	42.528192000	29.116398000
6	35.223263000	35.747852000	25.522515000
6	34.943329000	37.200647000	25.862027000
6	34.487584000	38.154463000	24.953486000
6	34.231285000	39.461918000	25.370135000
6	34.438209000	39.800377000	26.705239000
6	35.127998000	37.626502000	27.185434000
6	34.234772000	41.155410000	27.260918000
7	34.882292000	38.874172000	27.590913000
7	34.690082000	41.332420000	28.524248000

1	33.437780000	33.487649000	22.060524000
1	32.777120000	36.032550000	23.674577000
1	32.900724000	34.393983000	24.356398000
1	33.862315000	40.191059000	24.654967000
1	35.487979000	36.941768000	27.945723000
1	33.218523000	42.041586000	25.566331000
1	33.004068000	44.254242000	26.654880000
1	33.908737000	44.571628000	28.987934000
1	34.960338000	42.583732000	30.127906000
1	36.251371000	35.499874000	25.812025000
1	34.565404000	35.120070000	26.135358000
1	34.325632000	37.881669000	23.917172000
29	35.282726000	39.578878000	29.457393000
6	34.146926000	36.758143000	30.536891000
6	34.109064000	36.225214000	31.797807000
7	34.637535000	37.171099000	32.658004000
6	34.981665000	38.256189000	31.903253000
7	34.695320000	38.013382000	30.619793000
6	34.780363000	36.975364000	34.097210000
1	33.795883000	36.331680000	29.605578000
1	33.755637000	35.272215000	32.161661000
1	35.834801000	36.989776000	34.385758000
1	34.357449000	36.003652000	34.354986000
1	34.240981000	37.751466000	34.646356000
6	35.568818000	39.568320000	32.248340000
6	35.998775000	39.920482000	33.495172000
6	36.649125000	41.240652000	33.762935000
6	37.369450000	41.281320000	35.114640000
8	35.641888000	40.393797000	31.213233000
1	35.890608000	39.241038000	34.329752000
1	37.372471000	41.460448000	32.968792000
1	38.077807000	40.448711000	35.178221000
1	36.670422000	41.187114000	35.953287000
1	37.940126000	42.208707000	35.235227000
6	32.641147000	43.248065000	36.611965000
6	32.528944000	42.012028000	35.959643000
6	33.425628000	41.630784000	34.954386000
6	34.452346000	42.506565000	34.611128000
6	34.532497000	43.737223000	35.279065000
6	33.657527000	44.145794000	36.277261000
6	35.576396000	42.435832000	33.608304000
6	36.232090000	43.751683000	33.777894000
7	35.641481000	44.435354000	34.726780000
1	31.928005000	43.514939000	37.385164000
1	31.724893000	41.338271000	36.239458000
1	33.325691000	40.674867000	34.452324000
1	33.749572000	45.107604000	36.772411000
1	35.229898000	42.305494000	32.571620000
1	37.136087000	44.105902000	33.277262000
1	35.953765000	45.347233000	35.049017000
1	41.453702000	42.905811000	34.530718000
1	39.031961000	46.324294000	32.049852000
1	40.118849000	43.327538000	29.469148000
1	39.021840000	43.962166000	28.227836000
1	27.158993448	45.981487332	26.230065348
1	33.340422000	35.127191000	21.389222000
1	31.892112000	34.359719000	22.053072000
1	29.302082000	36.931363000	25.815386000
